# Other science opportunities at the FCC-ee

**DOI:** 10.1140/epjp/s13360-026-07399-w

**Published:** 2026-03-11

**Authors:** I. Agapov, E. E. Alp, K. Andre, S. Antipov, A. Apyan, G. Arduini, L. Bandiera, W. Bartmann, H. Bartosik, M. Benedikt, S. Bettoni, J. M. Byrd, M. Calviani, A. Camper, C. Carli, S. Casalbuoni, A. Chance, P. Craievich, P. Crivelli, B. Dalena, M. Dickmann, M. Doser, I. Drebot, C. Duchemin, K. Dupraz, A. Frasca, S. J. Freeman, F. Gunsing, J. Jäckel, B. King, M. W. Krasny, A. Lechner, C. C. Lindstrøm, A. Mazzolari, C. Milardi, E. Musa, R. Negrello, F. Nguyen, K. Oide, Y. Papaphilippou, G. Paternò, V. Petrillo, K. Piotrzkowski, B. Rienäcker, G. Schnell, C. Schroer, I. Schulthess, L. Serafini, V. Shiltsev, M. Stampanoni, A. Variola, T. Watson, H.-U. Wienands, M. Wing, F. Zimmermann

**Affiliations:** 1 Deutsches Elektronen-Synchrotron DESY, Hamburg, Germany; 2 Argonne National Laboratory, Lemont, IL USA; 3 European Organization for Nuclear Research (CERN), Geneva, Switzerland; 4 A.I. Alikhanyan National Laboratory, Yerevan, Armenia; 5 INFN Ferrara, Ferrara, Italy; 6 University of Ferrara, Ferrara, Italy; 7 PSI, Villigen, Switzerland; 8 University of Oslo, Oslo, Norway; 9 European XFEL, Hamburg, Germany; 10 CEA Irfu, University Paris-Saclay, Gif-sur-Yvette, France; 11 ETH Zürich, Zurich, Switzerland; 12 University of the Bundeswehr Munich, Neubiberg, Germany; 13 INFN Milano, Milan, Italy; 14 IJCLab, Orsay, France; 15 University Liverpool, Liverpool, UK; 16 University of Manchester, Manchester, UK; 17 University of Heidelberg, Heidelberg, Germany; 18 University of Plymouth, Plymouth, UK; 19 LPNHE, Paris, France; 20 INFN LNF, Frascati, Italy; 21 ENEA, Frascati, Italy; 22 University of Geneva, Geneva, Switzerland; 23 University Milano, Milan, Italy; 24 AGH University, Cracow, Poland; 25 EHU Quantum Center and Department of Physics, University of the Basque Country UPV/EHU, Bilbao, Spain; 26 IKERBASQUE, Bilbao, Spain; 27 Northern Illinois University, DeKalb, USA; 28 INFN Roma1, Rome, Italy; 29 UCL, London, UK

## Abstract

The Future Circular Collider (FCC) *integrated programme* begins with the FCC-ee, an electron-positron collider, followed by the FCC-hh, a proton–proton collider installed in the same 91 km circumference tunnel near CERN. Spanning 15 years from the mid-to-late 2040s through the early 2060s, the FCC-ee will operate at centre-of-mass energies between approximately 90 and 365 GeV, consistently delivering the highest possible luminosities to four experiments in a sustainable and energy-efficient manner. A key element of its design is top-up injection from a full-energy booster housed in the same 91 km tunnel, along with the world’s most intense positron source and 20 GeV injector linacs. The FCC-ee injector complex, comprising a high intensity positron source, a damping ring, and a linac accelerating electrons and positrons up to 20 GeV, is expected to start operation several years earlier than the booster and the collider. The primary objective of the FCC-ee is its rich High Energy Physics programme based on electron-positron collisions at various centre-of-mass energies (Benedikt et al. in Eur Phys J C 85:1468 10.1140/epjc/s10052-025-15077-x, 2025). In addition, thanks to its large circumference, high beam energy, abundant positron production, and low-emittance beams, the FCC-ee also offers unique opportunities for various fields of physics and science. These include the potential production of true muonium, the creation of a Bose-Einstein condensate of positronium, Compton imaging with high-energy photons, the generation of spatially coherent photon beams, possibly down to 0.1 Åwavelengths—achieving several orders of magnitude higher average and peak brightness than any existing or planned light source—radioactive isotope production, and an electron- or photon-beam-driven neutron source. We present these and other science exploitations of the FCC-ee accelerator complex.

## Introduction

The FCC-ee collider [2, 3] is a key component of a proposed integrated research programme probing energy scales up to 100 TeV, potentially spanning the entire 21st century (see, e.g. [1, 4]). Its primary goal is to enable precision measurements at the W, Z, Higgs, and $\textrm{t}\bar{\textrm{t}}$ energy scales, while establishing the bulk of the civil infrastructure for a possible future $\sim$100 TeV collider, FCC-hh.

Beyond its collider programme, from the 2040s through the 2060s, the unique capabilities of FCC-ee should be leveraged to explore new scientific opportunities. The key features include:

- An extremely low-emittance storage ring for both the FCC-ee booster and the collider, operating at the world’s highest beam energies with an exceptionally large bending radius, resulting in minimal dispersion.
- Electron beams at the highest energies, ranging from 20 GeV to $\sim$183 GeV.
- The world’s most intense positron source, which could be combined, e.g. with antiprotons from the CERN PS/AD complex to create an ultimate antimatter factory and enable positronium production.
- High-energy positron beams up to about 183 GeV. A $\sim$44 GeV positron beam extracted from the booster could be used for producing true muonium, and also provide a pathway towards a muon beam and, perhaps, even a future muon collider.
- The generation of high-energy $\gamma$ beams via various mechanisms, such as Compton backscattering off a laser, or by using advanced undulators.
- Unprecedented high-power beamstrahlung photons, reaching up to 0.5 MW per beam and per collision point, which could be exploited for applications, such as radioactive isotope production.
- A photon or electron beam-based short-pulse neutron source.

## FCC accelerator capabilities

### Collider

**Fig. 1:**
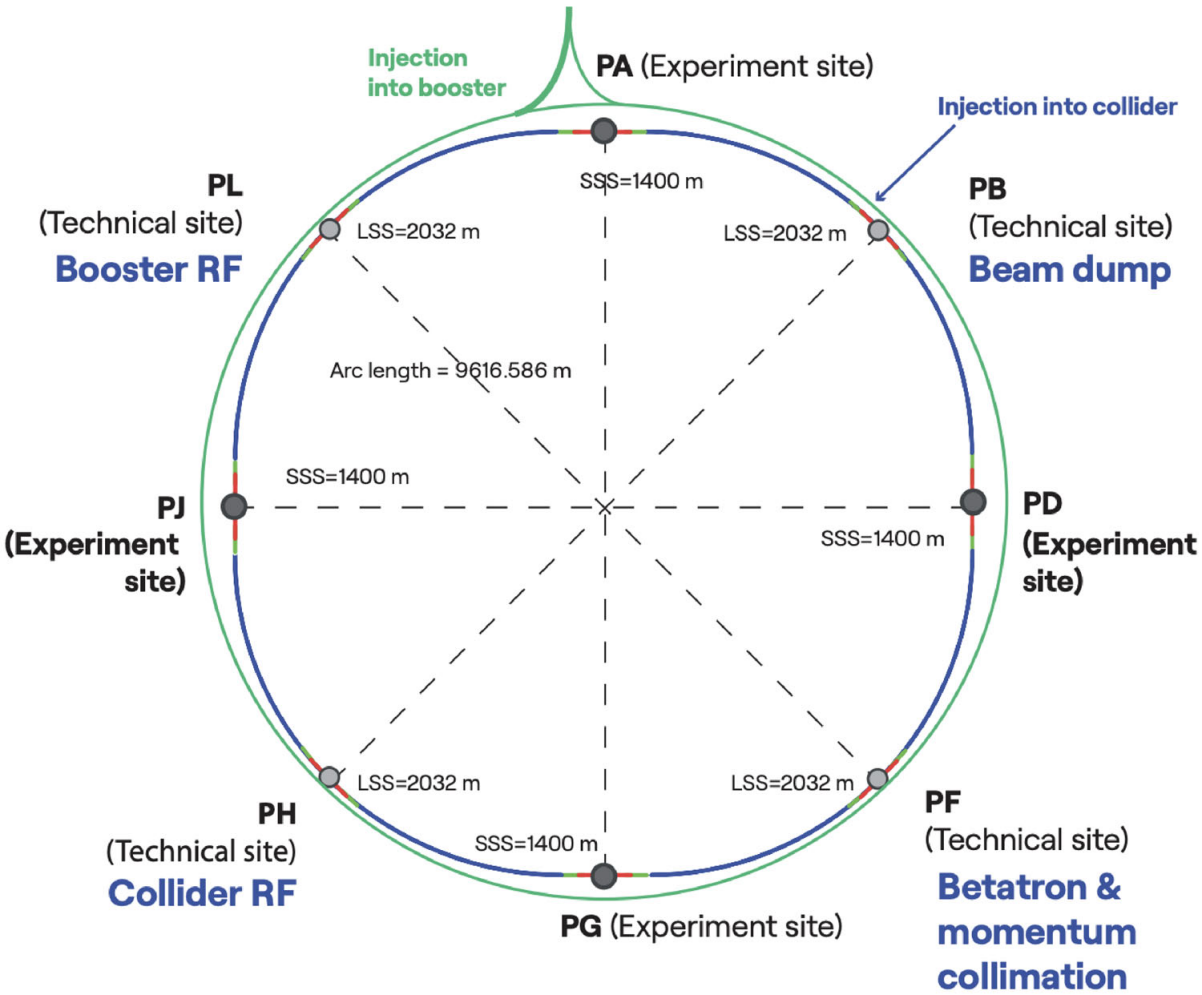
Layout of the FCC-ee illustrating the four collision points and the four technical insertions [3]

The FCC-ee is conceived as a double-ring collider with separate beam pipes for electrons and positrons. Its luminosity is maximised by regular top-up injection from a full-energy booster synchrotron, which must also be located in the collider tunnel (sketched schematically by the green circle in Fig. 1). Transfer lines connecting from the FCC-ee injector on the CERN Prévessin site to the booster are indicated schematically around Point A (PA).

For the present operation baseline, the time between injections varies between 3.8 s and 10 s, as is illustrated in Fig. 5. This is because the booster is assumed, on each cycle, to accelerate only 10% of the total number of bunches circulating in the collider. If instead the booster accelerated the full number of bunches, i.e. the same number as in the collider, the injection cycle period could be on the order of 1 min.

The FCC-ee will operate at four main working points, corresponding to the Z pole (91 GeV centre of mass), the WW threshold (120 GeV), the ZH production peak (240 GeV), and the $\textrm{t}\bar{\textrm{t}}$ threshold (up to 365 GeV). The latest set of parameters is presented in Table 1. The bunch population is held approximately constant for all modes of operation (within a factor of 1.6, i.e. varying between $1.38\times 10^{11}$ and $2.18\times 10^{11}$ electrons or positrons per bunch), while the number of bunches is widely changed, by a factor of about 200, to adjust the beam current. The beam current is limited by the synchrotron radiation power, and strongly decreases at higher beam energies.

For the Z operating point a large number of about 11,000 bunches will be stored in each of the two collider rings. In this case, the bunches would be separated into 40 bunch trains of 280 bunches with a bunch-to-bunch separation of 25 ns and a train-to-train separation of roughly 0.6 $\upmu$s. Similarly, at the W with 1780 bunches, bunches might be separated into 20 trains of 89 bunches having a bunch-to-bunch separation of roughly 150 ns and a train-to-train separation of roughly 2 $\upmu$s. At the ZH and $\textrm{t}{\bar{\textrm{t}}}$ it is likely that the bunches will be uniformly distributed around the ring. In Table 1, two contributions to the beam lifetime are indicated separately: (1) the effect of lattice dynamic aperture and beamstrahlung plus quantum fluctuation (“q+BS+lattice”), and (2) the unavoidable luminosity-related radiative Bhabha scattering (“lum.”). The total beam lifetime is the inverse of the sum of the individual inverse lifetimes. Using top up injection, the two beam currents are held constant to within a few percent.

**Table 1:** Parameters of FCC-ee [3]

Running mode	Z	W	ZH	$\textrm{t}{\bar{\textrm{t}}}$
Number of IPs	4	4	4	4
Beam energy (GeV)	45.6	80	120	182.5
Bunches/beam	11200	1780	440	60
Beam current [mA]	1283	135	26.8	5.1
Bunch population [$10^{11}$]	2.18	1.38	1.69	1.58
Luminosity/IP [10$^{34}$ cm$^{-2}$ s$^{-1}$]	145	20	7.5	1.41
Energy loss / turn [GeV]	0.039	0.369	1.86	9.94
Synchrotron Radiation Power [MW]	100	100	100	100
RF Voltage 400/800 MHz [GV]	0.08/0	1.0/0	2.1/0	2.1/9.2
RMS bunch length (SR) [mm]	5.53	3.46	3.26	1.91
RMS bunch length (+BS) [mm]	15.7	5.28	5.59	2.33
RMS horizontal emittance $\varepsilon _{x}$ [nm rad]	0.71	2.16	0.66	1.51
RMS vertical emittance $\varepsilon _{y}$ [pm rad]	2.3	2.0	1.0	1.4
Longitudinal damping time [turns]	1171	218	65.4	19.6
Horizontal IP beta $\beta _x^{*}$ [mm]	110	220	240	900
Vertical IP beta $\beta _y^{*}$ [mm]	0.7	1.0	1.0	1.4
Hor. IP beam size $\sigma _x^{*}$ [µm]	9	22	13	37
Vert. IP beam size $\sigma _y^{*}$ [nm]	40	45	32	44
Beam lifetime (q+BS+lattice) [min.]	220	75	100	105
Beam lifetime (lum.) [min.]	22	16	10	11
Total beam lifetime [min.]	21	13	9	10
Total int. annual luminosity [ab$^{-1}$/yr]	68$^{\dagger }$	9.6	3.6	0.67$^{\ddagger }$

Peak luminosity values are given per interaction point (IP), for a total of 4 IPs. Integrated luminosities refer to the sum over four IPs. Both natural bunch lengths due to synchrotron radiation (SR) and collision values including beamstrahlung (BS) are shown. The FCC-ee collider rings feature a combination of 400 MHz RF systems (at the first three energies) and 800 MHz (additional cavities for $\textrm{t}\bar{\textrm{t}}$ operation), with voltage strengths, respectively, indicated. For the integrated luminosity, 185 days of operation per year, and luminosity production at 75% efficiency with respect to the ideal top-up running is assumed, as in the report [5]

$^{\dagger }$The integrated luminosity in the first two years is assumed to be half this value to account for the machine commissioning and beam tuning;

${^\ddagger }$ The integrated luminosity in the first year, at the slightly lower beam energy of 170 to 175GeV, is assumed to be about 65% of this value to account for the machine commissioning and beam tuning. The smaller time for commissioning compared with the lower energy running reflects the LEP/LEP-2 experience

**Fig. 2:**
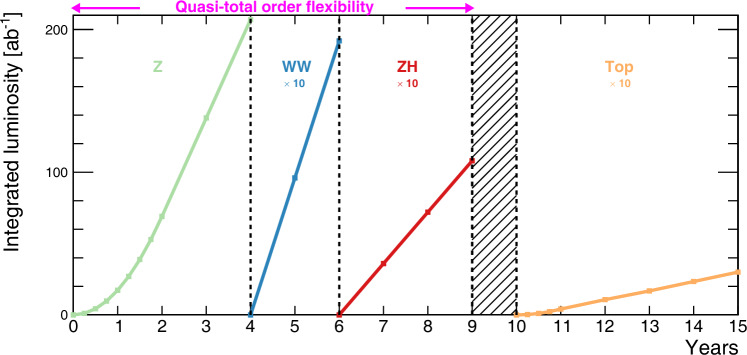
Operation sequence for FCC-ee with four interaction points, showing the integrated luminosity at the Z pole (green), the WW threshold (blue), the Higgs factory (red), and the top-pair threshold (orange) as a function of time [3]. In this baseline model, the sequence of events goes with increasing centre-of-mass energy, but there is quasi-total flexibility in the sequence all the way to 240 GeV. The integrated luminosity delivered during the first two years at the Z pole and the first year at the tŧ threshold is half the annual design value. The hatched area indicates the shutdown time needed to prepare the collider for the higher energy runs at the top-pair production threshold and above

Figure 2 displays the baseline sequence of events [6], corresponding to the operation model of Table 2. However, other mode sequences would be possible and might be preferred. For example, one can consider scheduling a Z pole run after the ZH run or the WW threshold run, ideally with a first Z pole run during the initial period of FCC-ee operation. The versatile RF system of the FCC-ee enables a near-total flexibility for choosing the running sequence or changing the collision energy between the first three running modes. For example, it would allow for short initial Z pole and WW threshold runs, to commission the collider and the detectors, in order to establish the resonant depolarisation procedures, etc. The ZH run could then proceed, before going back to the Z pole and the WW threshold, both now at full luminosity, with fully functional resonant depolarisation, and complete understanding of the collider.

**Table 2:** The baseline FCC-ee operation model with four interaction points (IPs), showing the centre-of-mass energies, design instantaneous luminosities for each IP, integrated luminosity per year summed over 4 IPs [6]

Working point	Z pole	WW thresh.	ZH	$\textrm{t}{\bar{\textrm{t}}}$	
$\sqrt{s}$ (GeV)	88, 91, 94	157, 163	240	340–350	365
Lumi/IP ($10^{34}\,\textrm{cm}^{-2}\textrm{s}^{-1}$)	140	20	7.5	1.8	1.4
Lumi/year ($\textrm{ab}^{-1}$)	68	9.6	3.6	0.83	0.67
Run time (year)	4	2	3	1	4
Integrated Lumi ($\textrm{ab}^{-1}$)	205	19.2	10.8	0.42	2.70
			$2.2\times 10^6$ HZ	$2\times 10^6\, \textrm{t}{\bar{\textrm{t}}}$	
Number of events	$6\times 10^{12}$ Z	$2.4\times 10^8$ WW	+	$+370$kHZ	
			65k WW $\rightarrow$ H	$+92$k $\textrm{WW}\rightarrow \textrm{H}$	

The integrated luminosity values correspond to 185 days of physics per year and 75% operational efficiency (i.e. $1.2\times 10^7$ seconds per year) [5], in the Z, WW, ZH, $\mathrm{t}{\bar{\mathrm{t}}}$ baseline sequence. The last two rows indicate the total integrated luminosity and the total number of events expected to be produced in the four detectors

### Subsurface infrastructure: service caverns and bypass tunnels

**Fig. 3:**
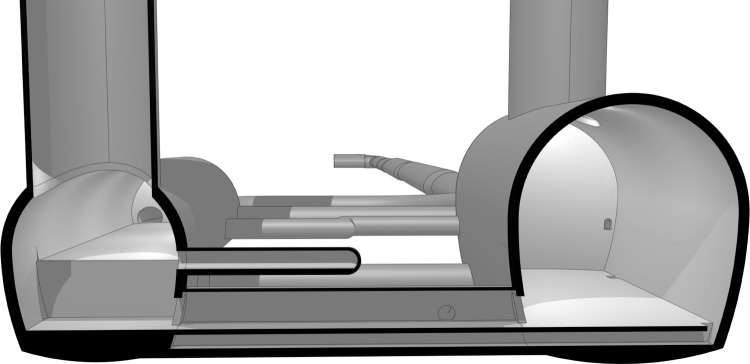
Cross section through the 3D model at PA, showing the service cavern (left) and experiment cavern (right) [7]

Large-span caverns at experimental points PA and PG, illustrated in Fig. 3, are designed to accommodate the main FCC-hh detectors and associated infrastructure. The proposed cavern dimensions are 66 m $\times$ 35 m $\times$ 35 m (L$\times$W$\times$H) and the caverns will be constructed at a depth of up to 226 m in the molasse rock. A service cavern at the same elevation as the accelerator tunnel with dimensions of 100 m $\times$ 25 m (length $\times$ width) is required adjacent to each of the four FCC-ee/hh experiment caverns.

**Fig. 4:**
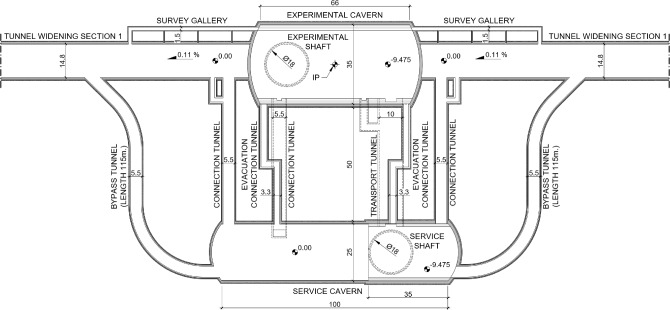
Top view of PA showing the layout of bypass and connection tunnels between the service cavern and experiment cavern [7]

Figure 4 presents a typical layout of underground infrastructure around a collision point, consisting of experiment and service caverns, bypass tunnels, connections and survey galleries. Below the service shaft, the height of the service caverns is 22.4 m, thereby providing direct access to the experiment cavern floor for large equipment and detector components. The remainder of the 100 m long service cavern has a height of 15 m. The service caverns will house infrastructure equipment such as electrical, cooling, ventilation and cryogenics. The spacing between the experimental cavern and the service caverns is approximately 50 m, to mitigate electromagnetic effects from the detector on the nearby electrical components. The service caverns will contain three floor levels. This greatly increases the usable space for technical infrastructure and services. The dimensions of the service caverns are determined by the needs of the FCC-hh collider, and a large portion of this cavern is empty during the FCC-ee era, when this space could be devoted to photon-science applications.

Bypass tunnels at each of the four experiment areas allow access for transport, personnel, and services directly from the service cavern to the accelerator tunnel, therefore bypassing the experiment cavern and detector areas. These tunnels have an internal diameter of 5.5 m, similar to the cross section of the accelerator tunnel. The length of the bypass tunnels varies between 110 and 115 m. The bypass tunnels connect the service cavern to the accelerator tunnel within the first section of the tunnel widening, approximately 74 m from the experiment cavern. The junction between the accelerator tunnel and bypass tunnel is shown at an angle of 45 degrees. However, transport needs may require a shallower angle to be specified. In the next design phase, the details of the junctions between the two tunnels will be reassessed, and if required, a junction cavern or other more effective means of accommodating the connection will be implemented. In particular, special requirements for photon-science applications could be taken into account in the civil engineering design during the next study phase.

Connection tunnels between the service caverns and experiment caverns provide access for personnel and transport of materials/equipment. These tunnels also house the service ducts, cables, and pipes linking the service caverns to the detectors and accelerator tunnels.

### Booster

The full-energy booster receives electron or positron beams (alternating each cycle) at 20 GeV and accelerates them to the FCC-ee collision energy. Depending on the operation mode, the booster cycle time ranges from a few seconds to 10 s, with a final beam energy of up to 182.5 GeV. The number of bunches in the booster also varies based on the collider’s operation mode, which is maintained at constant synchrotron radiation power.

**Fig. 5:**
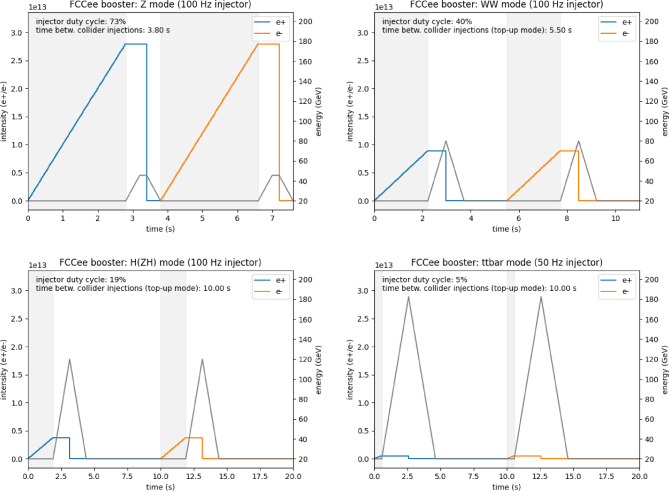
Schematic view of the booster cycles (indicated by black lines) for positron and electron top-up (intensity indicated by blue and orange lines) of the FCC-ee collider for the Z mode (top left), the WW-mode (top right), the ZH mode (bottom left) and for the $\textrm{t}\bar{\textrm{t}}$ mode (bottom right) operation. The black lines correspond to the right vertical axis (energy) and the coloured lines to the left one (intensity). The grey shaded areas indicate the time that the injector is needed to provide the beam for the booster

A schematic of the booster cycles for different operation modes is shown in Fig. 5. During Z-pole operation, the booster will be fully occupied with continuous top-up injections. In the other three modes, there will be intervals between booster cycles that could be used to deliver beams for additional physics experiments.

### Injector

**Fig. 6:**
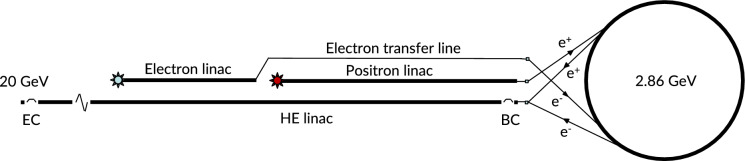
Schematic representation of the proposed FCC-ee injector complex with a damping ring on the right [3]

The FCC-ee requires a dedicated injector complex for delivering positron and electron beams to the booster for the top-up operation of the collider. The presently studied injector complex, as shown in Fig. 6, consists of a low energy electron linac, a positron linac, and a damping ring (DR) at 2.86 GeV. For positron production, the electron beam from the low energy linac is sent onto a positron target, and a further linac accelerates the resulting positron beam to the damping ring injection energy of 2.86 GeV. A high energy (HE) linac accelerates either positrons or electrons to the final energy of 20 GeV for injection into the booster. This injector complex is planned to be placed on the CERN Prevéssin site, with the beam exiting from the high energy linac close to the experimental facilities in the CERN North Area, which could provide additional science opportunities beyond collider physics, by exploiting the intense high-energy electron and positron beams, with energies up to 20 GeV or above, at existing or future fixed-target experiments, or driving a Free Electron Laser (FEL).

The FCC-ee injector complex will provide pulses of 4 bunches with a bunch spacing of 25 ns, with $2.5\times 10^{10}$ particles per bunch (corresponding to 4 nC). A summary of relevant beam parameters is given in Table 3. The duty cycle of the injector complex required for providing beam to the booster in top-up mode varies significantly depending on the operation mode of the collider. The injector complex will be used with a duty cycle of 73 % for the Z pole, 40 % in *WW*, 19 % for ZH and 5 % for $\textrm{t}\bar{\textrm{t}}$.

**Table 3:** Beam parameters of the FCC-ee injector complex

	FCC injection	other users
Beam energy [GeV]	20	$\le 20$
Max. bunch charge [nC]	4	4
Max. bunch population [$10^{10}$]	0.1–2.5	2.5
Bunches per pulse	2–4	1–4
Linac repetition rate [Hz]	50–100	100
Norm. emittance *x*/*y* [mm mrad]	$\le 20/2$	$\le 20/2$
Physical emittance *x*/*y* [nm rad]	$\le 0.5/0.05$	$\le 0.5/0.05$
RMS bunch length [mm]	4	1–4
RMS energy spread [%]	0.1	0.1–0.75
Bunch spacing [ns]	25	25 or 50
injector duty cycle [%]	5–73	27–95

## Photon science and extreme photon science

### Injector linac as XFEL driver

The injector linac offers promising potential to serve as a photon light source. While detailed simulations are mandatory for more realistic performance evaluation, preliminary considerations can be made based on conservative electron beam parameters inspired by running FEL facilities. The parameters of the electron bunch train at the exit of the high-energy (HE) linac, comprising four bunches separated by 25 ns and operated at a repetition rate of 100 Hz, are listed in Table 4.

**Table 4:** Electron beam parameters at the exit of the HE linac for the XFEL beamline

Beam energy [GeV]	25
Bunch charge [pC]	250
RMS bunch length [fs]	50
Peak current [kA]	5
Normalised emittance [mm$\cdot$mrad]	0.4
RMS energy spread [‰]	0.2
Beta function x,y [m]	30

The bunch train consists of four bunches separated by 25 ns at 100 Hz repetition rate

The photocathode RF gun is capable of generating electron bunches with an RMS normalised emittance of 0.4 mm$\cdot$mrad at a bunch charge of 250 pC in both transverse planes *x* and *y*, and an RMS bunch length of approximately 3 ps. Assuming these parameters, the bunches are assumed to undergo a total compression factor of about 60 distributed over multiple stages to reach the target current of 5 kA.

The layout of the FCC-ee injector allows introducing the first compression stage in multiple ways. The first possibility is using the bunch compressor downstream of the DR already foreseen in the FCC-ee design. In this case, assuming a four-bend chicane, the required off-crest RF operating phase will be used both to generate the bunch energy chirp and to counteract the short-range wakefield-induced chirp. Alternatively, the first stage bunch compression can be achieved using the DR arc operated as a bunch compressor. In this case the energy-time correlation term, $R_{56}$, can be tuned to have a sign opposite to that of a four dipole chicane, so that the short-range wakefields in the upstream linac can be utilised to increase the energy chirp for the bunch compression. This would improve the beam stability [8] and mitigate effects of coherent synchrotron radiation (CSR) effects. A third option combines both methods: tuning the DR optics for partial compression ($R_{56}$ with the same sign as that of a four-bend chicane) and applying additional compression using the chicane downstream of DR. Another possibility is based on the use of the DR as a usual damping ring to stabilise and improve the bunch transverse quality, but so as to increase the energy spread, at the expense of a larger bunch length, which must be further shortened in the downstream compression stages.

If simulations indicate that the microbunching instability will be detrimental to the beam quality, the installation of an additional bunch compressor in the low-energy section may be considered, e.g. at the beginning of the electron linac, where the beam energy is on the order of a few hundred MeV. This is not necessary for the FCC-ee injector when used as the driver to the collider, but it would be beneficial also here, leading to a reduction of the bunch transverse jitter at the positron production target. The transverse wake fields, which increase with longer bunches, presently limit the minimum apertures of RF structures in the electron linac.

The FCC-ee injector linac layout includes an Energy Compressor (EC) located at the end of the high-HE linac. This system could be employed as a second-stage bunch compressor. In the case of FCC-ee the necessity of removing the bunch energy chirp residual from the compression does not represent a show-stopper for the limited amount of RF structures downstream of the EC chicane (energy variation of about 650 MeV) because of the relatively large value of $R_{56}$ (about 0.5 m in the present design). Alternatively, the EC may be used to vary the energy spread after compressing the beam to a higher peak current than the target value in the upstream sections, prior to injection into the undulator lines.

At present, conservative assumptions have been made for the bunch charge and duration, based on parameters from existing free-electron laser (FEL) facilities. However, given that the RF structures in the linacs are designed to transport multi-nC bunches, future optimisation of the injector should also explore the possibility of increasing the bunch charge. This approach could allow for a reduction in the overall compression factor, thereby helping to mitigate emittance growth and, eventually, to reduce the final energy spread.

Currently, an electron beam energy of 20 GeV is foreseen at the exit of the FCC-ee injector. In order to push the photon beam energy from a possible XFEL to even harder X-rays, an electron beam energy of 25 GeV has been considered. This could be achieved by doubling the number of klystrons in the HE linac, using two RF structures per klystron instead of the four structures foreseen for the FCC-ee injector baseline. The required additional space for high-voltage klystron modulators must be considered during civil infrastructure planning, as this scheme would likely necessitate a larger klystron gallery.

Assuming the beam parameters from Table 4, and combining them with superconducting undulators (SCUs), the injector could drive an X-ray Free Electron Laser capable of producing hard to very hard X-rays. The expected photon pulse energies are in the milli-Joule range, as summarised in Table 5 [9].

**Table 5:** SCU and photon pulse parameters for potential very hard X-ray XFEL beamlines driven by the FCC-ee HE linac [9]

SCU Technology	NbTi@4K	HTS@4K
Beam stay clear [mm]	5	5
Period length [mm]	18	13
Magnetic field *B* [T]	1.83	2.2
Saturation length [m]	85	100
Minimum photon energy [keV]	60	100
Photon pulse energy [mJ]	0.95	0.4

Two SCU technologies are considered: NbTi-based SCUs, currently employed at several storage rings and proposed as an afterburner solution at the European XFEL; and High-Temperature Superconductor (HTS)-based SCUs, which still remain under development.

As illustrated in Fig. 5, the injector linac is compatible with simultaneous XFEL operation in the WW, ZH, and $\textrm{t}\bar{\textrm{t}}$ FCC-ee collider modes. However, in the Z mode, only a limited number of bunches may be available for XFEL use.

Although further studies are needed to investigate the implementation of this option, a very hard X-ray FEL is a very appealing application of the FCC-ee injector, offering unique capabilities to reach mJ class photon pulse energy above 50 keV.

### High-energy photons from the FCC-ee booster

The large FCC-ee rings are outstanding: they combine an extremely low emittance and a high beam current (up to about 1.5 A in the collider, and of order 10 mA average current in the full-energy booster, to be compared with 30 $\mu$A at the European XFEL, a difference by three orders of magnitude or more). Photon energies from an undulator or wiggler increase as $\gamma ^2$. Radiation remains spatially coherent for photon wavelengths $\lambda > 4 \pi \varepsilon$. For constant cell-length the horizontal emittance $\varepsilon$ in a storage ring scales as $\gamma ^2/\rho ^3$ [10]. With the extremely large radius $\rho$ of the FCC, one can achieve such a small emittance that high brilliance and coherent radiation could be reached also at high photon energies, inaccessible with the presently existing machines or at other planned future light sources. At injection energy the FCC-ee booster offers the lowest emittance. The emittance can be further lowered by introducing damping wigglers or undulators, which could be the same as those generating light for the photon science.

**Fig. 7:**
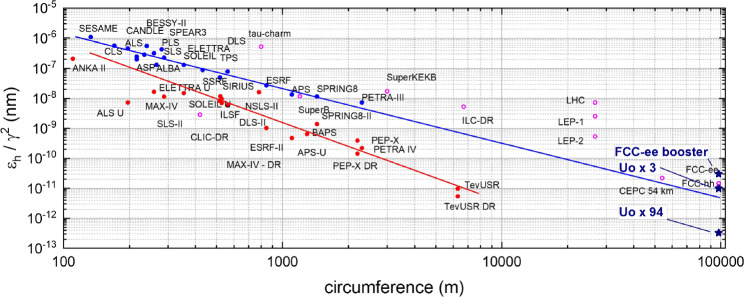
Horizontal emittance divided by $\gamma ^2$ as a function of ring circumference, with the FCC-ee booster on the far right (courtesy R. Bartolini, adapted)

Many storage ring photon sources around the world are undergoing upgrades to decrease the horizontal emittance. The FCC-ee booster naturally has a small horizontal emittance, even at high energy, as a result of the large circumference. As stated, adding damping wigglers or undulators further reduces this emittance. For the FCC-ee collider baseline, it is foreseen to add wigglers in order to increase the energy loss per turn $U_0$ by a factor 3, in order to prevent the microwave instability [11]. For photon science applications, we can add further wigglers and undulators, up to an ultimate value close to $100\times U_0$ (in the following we consider 94 $U_0$). Figure 7 compares the resulting emittance normalised to $\gamma ^2$ with other existing or proposed future storage ring light sources. The FCC-ee arc optics is based on variants of a FODO lattice, since the latter maximises the dipole filling factor, thereby minimising the energy loss per turn and the radiofrequency (RF) voltage required. By contrast, modern light-source lattices are optimised for low emittance. For FCC-ee, the low emittance arises as a byproduct of the large bending radius, and the implied low dispersion function. As illustrated in Fig. 7, the $U_0 \times 94$ option brings the FCC-ee result closer to the line of the 4th generation light sources.

**Fig. 8:**
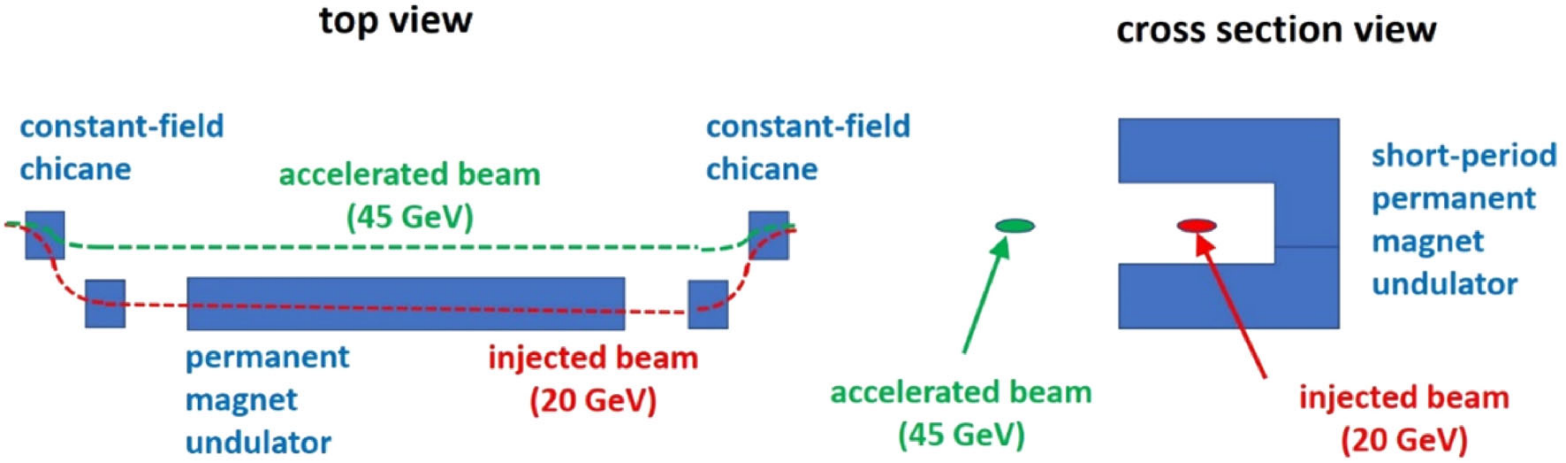
Sketch of permanent-magnet undulator and fixed-field chicane for the FCC-ee booster with beam orbit at 20 GeV injection and at higher energy [12]

Without any wigglers, at the injection energy of 20 GeV the electron or positron beams in the booster experience an energy loss per turn of $U_0 = 1.33$ MeV, and feature an RMS equilibrium emittance of 46 pm horizontally, and less than 5 pm vertically. Table 6 compares a few scenarios with additional wigglers at injection energy and at 45.6 GeV (extraction energy for collider Z pole operation). The additional wigglers and/or undulators will also have a damping and stabilising effect for the operation as collider injector. They could be realised as permanent-field C-shape magnets surrounded by a fixed-field chicane, as indicated in Fig. 8, where the beam naturally and automatically moves out of the wiggler during acceleration. It also is possible to separate the functions and use strong wigglers for shrinking the emittance and use an undulator for generating lower wavelength radiation. With these parameters, the peak and average brilliance can be roughly 1000 times higher than at proposed future storage ring light sources like PETRA IV in the very hard X ray regime, while coherent wavelengths extend downward by a factor 100, opening up new areas of science.

**Table 6:** Parameters used for the study of FCC-ee booster as a photon source, where $U_0$ is the energy loss per turn without any wigglers or undulators; $U_0=1.33$ MeV at 20 GeV beam energy [12]

Energy loss/turn	$3\times U_0$	$94\times U_0$	$94\times U_0$
Beam energy [GeV]	20	20	45.6
Average beam current [mA]	6	6	15
Average number of bunches	500	500	1120
RMS bunch length [mm]	4	9.5	4.4
RMS relative energy spread [$10^{-3}$]	0.4	2.2	0.4
Beta at wiggler / undulator [m]	1.6	1.6	1.6
Wiggler field [T]	1	1	1
Wiggler period [mm]	40	40	40
Magnetic gap [mm]	10	10	10
Total length wiggler [m]	6.4	264	264
Horizontal emittance [pm]	15	0.5	100
Vertical emittance [pm]	1.5	0.05	0.2
Time for users (s) over a cycle of 4 s	2.5	2.5	0.4

**Fig. 9:**
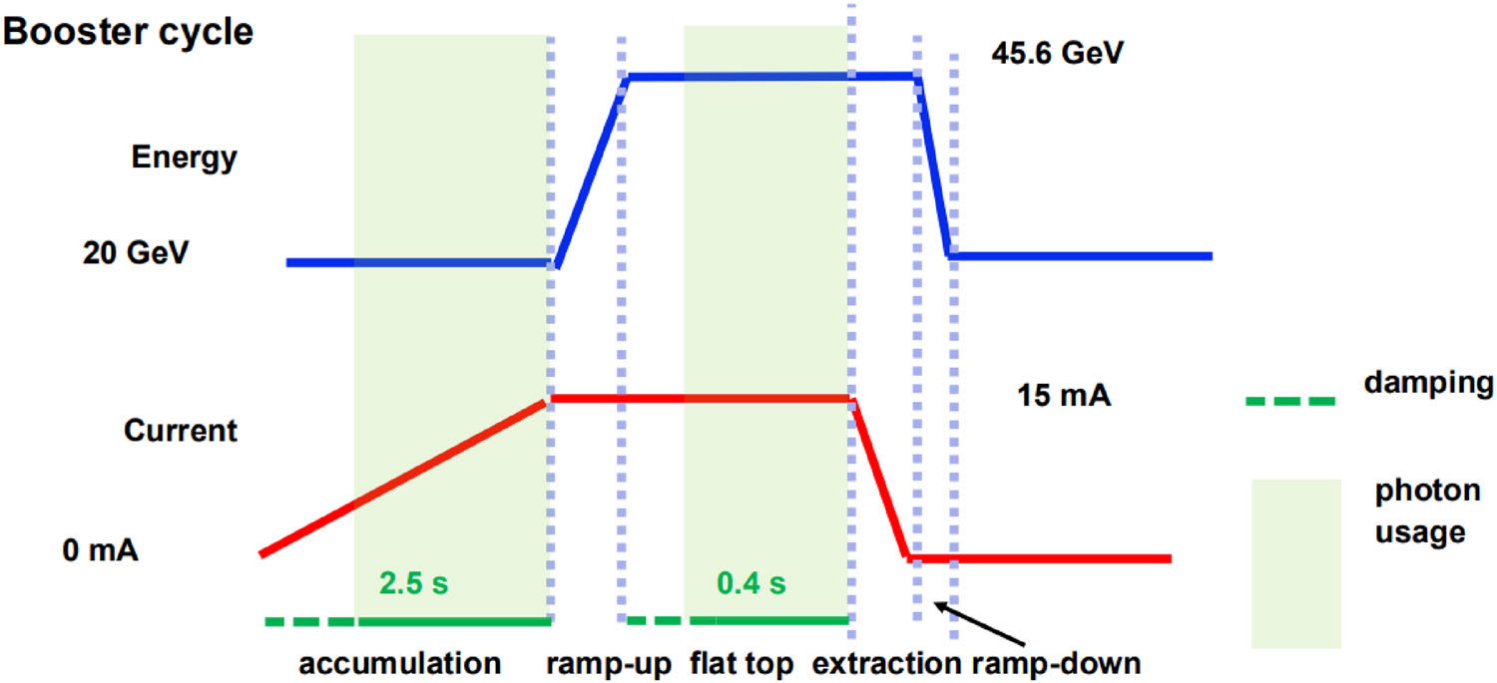
Schematic booster cycle with two operation periods for light-source applications [9]

Different from other light-source storage rings, the parasitic use of photons from the FCC-ee booster would not be continuous. Figure 9 presents a schematic booster cycle for Z-pole operation, including, after an initial radiation damping time, periods for photon usage at 20 GeV and at top energy, on the order of seconds. For this mode of operation, photons are provided to users roughly 50% of the time.

Parameters for wigglers and undulators based on permanent magnet technology are compiled in Table 7.

**Table 7:** Permanent magnet technology for undulator (photon generation) and damping wigglers [9, 12]

Magnetic gap [mm]	10
Max. undulator field [T]	0.71
Undulator period [mm]	28
Undulator unit length [m]	5
Wiggler field [T]	1
Wiggler period [mm]	40
Wiggler unit length [m]	6.4 or 5

In a diffraction-limited storage ring, if the transverse beam emittances are much smaller than the photon emittance, that is $\varepsilon _{x,y}\ll \varepsilon _\gamma$, the maximum brilliance is

1 $$\begin{aligned} B \approx \frac{\textrm{flux}}{4 \pi ^2 \varepsilon _{\gamma }^2} \approx \frac{4 {\textrm{flux}}}{\lambda ^2} \; . \end{aligned}$$

On the other hand, if $\varepsilon _{x,y}\approx \varepsilon _\gamma$, the brilliance is maximised if the electron and photon beam phase spaces are matched, which is achieved for a beta function at the centre of the undulator of length *L* equal to [13]

2 $$\begin{aligned} \beta _{x,y} = \frac{\sigma _{\gamma }}{\sigma _{\gamma }'} \approx \frac{L}{\pi } \; , \end{aligned}$$

in which case the brilliance is smaller by a factor of four,

3 $$\begin{aligned} B \approx \frac{\textrm{flux}}{4 \pi ^2 (\varepsilon _{\gamma }+\varepsilon _x) (\varepsilon _{\gamma }+\varepsilon _y) } \approx \frac{\textrm{flux}}{\lambda ^2} \; . \end{aligned}$$

**Fig. 10:**
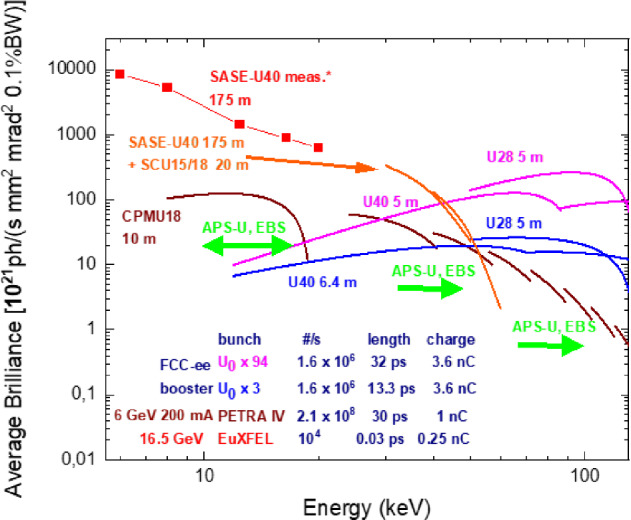
Average brilliance as a function of photon energy, comparing the FCC booster at injection energy (blue and purple), with the EuXFEL, the proposed EuXFEL upgrade, and the planned PETRA IV. Simulations were performed with SPECTRA [14]. The data points for the EuXFEL (red squares) are inferred from unpublished measurements of the pulse energy [9]

**Fig. 11:**
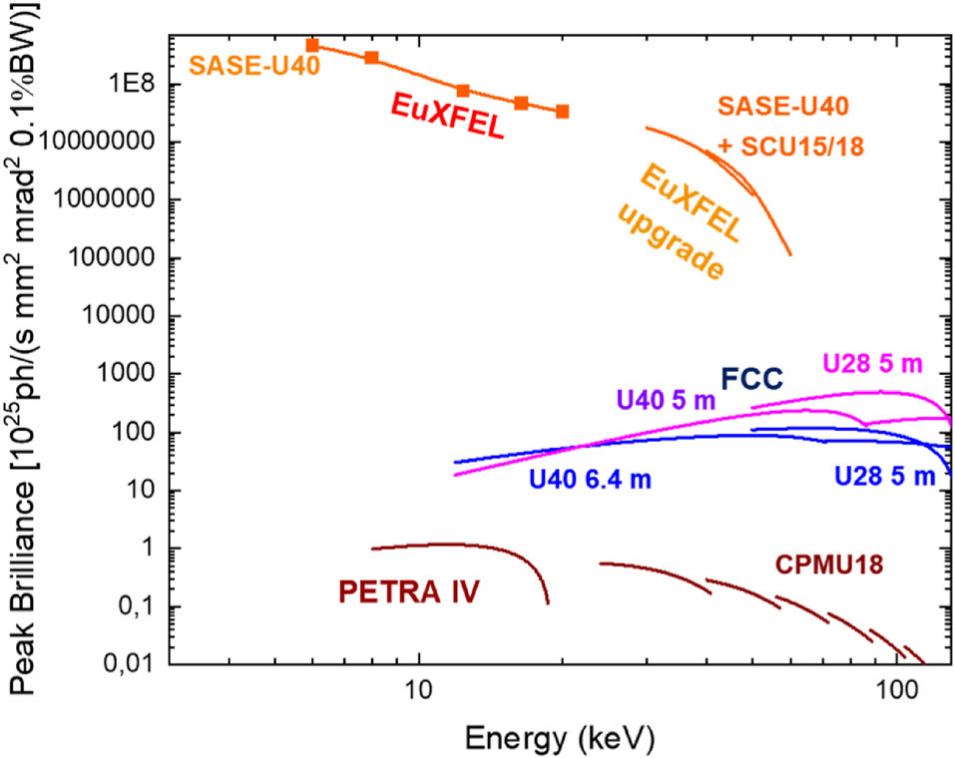
Peak brilliance as a function of photon energy, comparing the FCC booster at injection energy (blue and purple), with the EuXFEL, the proposed EuXFEL upgrade, and the planned PETRA IV. Simulations performed with SPECTRA [14]. The data points for the EuXFEL (red squares) are inferred from unpublished measurements of the pulse energy [9]

Figures 10 and 11 show the average and peak brilliance that can be achieved at the booster injection energy in the $3\times U_0$ or $94\times U_0$ damping scenarios, compared with other existing or proposed future light sources. In the photon energy range above about 50 keV the FCC-ee booster light source produces an average and peak brightness which is orders of magnitude higher than any existing or proposed light source. Efficient application of these highly would require beyond-state-of-the art imaging beamlines and likely an intensive collaboration with other leading actors such as ESRF, DESY, or PSI.

**Fig. 12:**
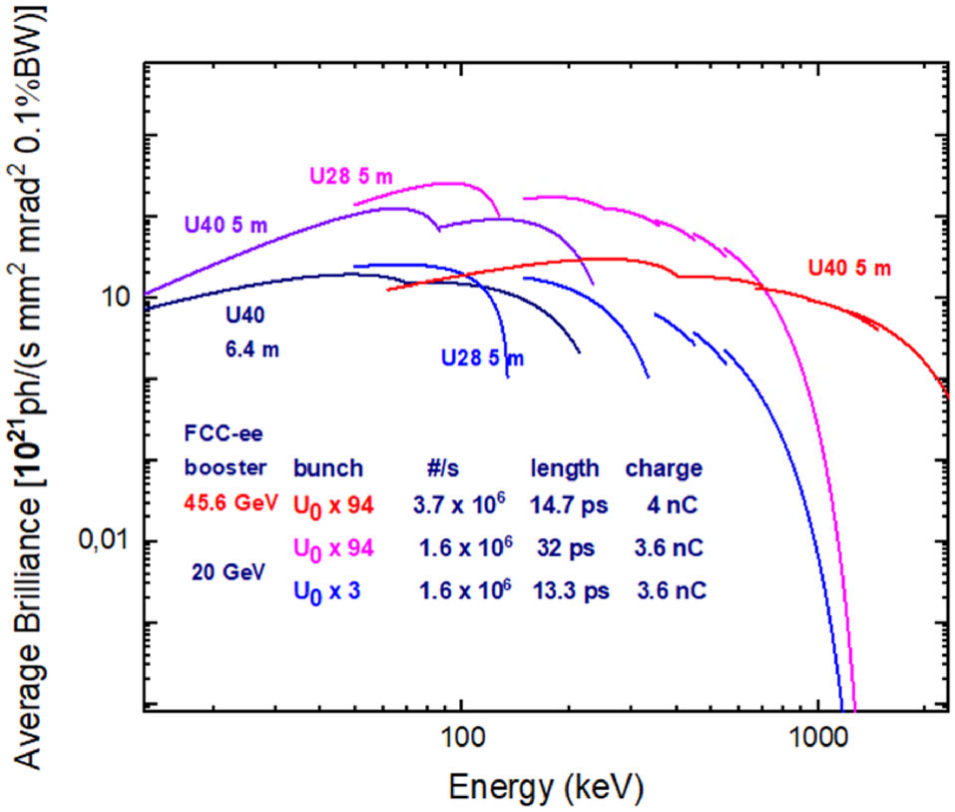
Average brilliance as a function of photon energy from 10 keV to 2 MeV, for a booster beam energy of 20 GeV and 45.6 GeV [9]. Simulations were performed with SPECTRA [14]

**Fig. 13:**
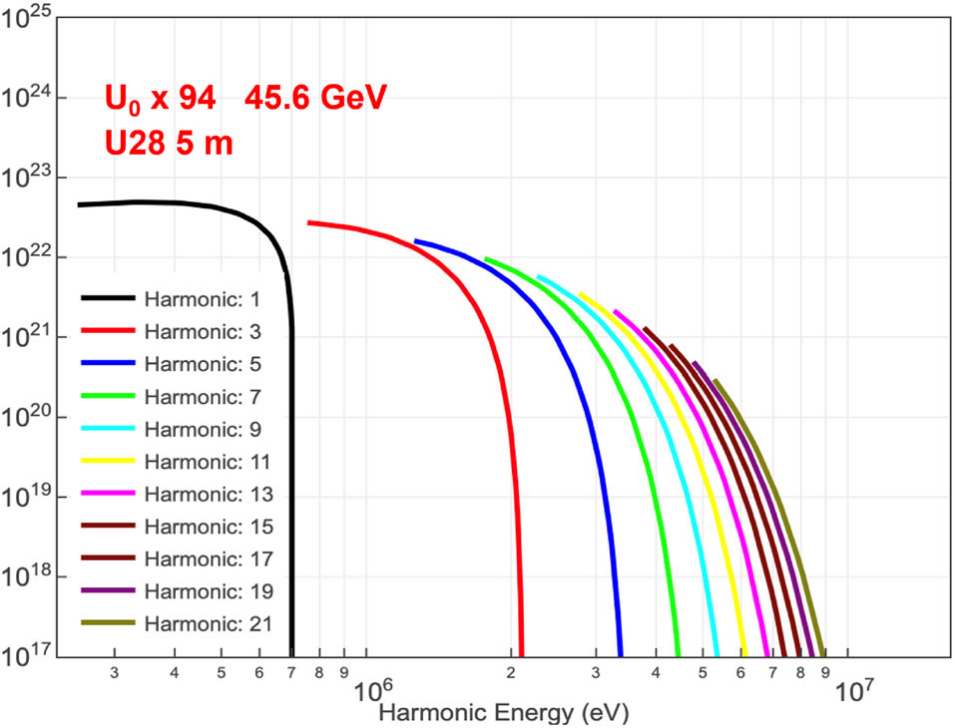
Average brilliance in units of photons per second per mm$^2$, per mrad$^2$ and per 0.1% BW, including higher harmonics as a function of photon energy from 200 keV to 20 MeV, for a booster beam energy of 45.6 GeV [9]. Simulations were performed with SPECTRA [14]

Figures 12 and 13 illustrate the brightness that can be achieved up to photon energies of 2 or 20 MeV, with a beam energy in the booster of up to 45.6 GeV, including higher harmonic radiation from the undulator.

The photons from a diffraction-limited storage ring are also characterised by a high transverse coherence. The coherent flux is a fraction $f_{c}$ of the total flux defined as

4 $$\begin{aligned} \mathrm{coherent\; flux} = f_{c} \; \textrm{flux}, \end{aligned}$$

where, for a round beam, with $\varepsilon _x = \varepsilon _y \equiv \varepsilon$,

5 $$\begin{aligned} f_{c} = \frac{\left( \lambda /(4 \pi ) \right) ^2}{\left( \varepsilon \frac{L}{\pi } + \frac{\lambda L}{4 \pi ^2}\right) \left( \varepsilon \frac{\pi }{L} + \frac{\lambda }{4 L}\right) }\; . \end{aligned}$$

For a flat beam as in the FCC-ee booster, and ignoring the effect of a momentum spread, the coherence fraction is determined by the horizontal coherence $f_{ch}$ as

6 $$\begin{aligned} f_{ch} = \frac{\lambda /(4 \pi ) }{\sqrt{\varepsilon _{x}\frac{L}{\pi } + \frac{\lambda L}{4 \pi ^2}} \sqrt{\varepsilon _{x}\frac{\pi }{L} + \frac{\lambda }{4 L}} }. \end{aligned}$$

**Fig. 14:**
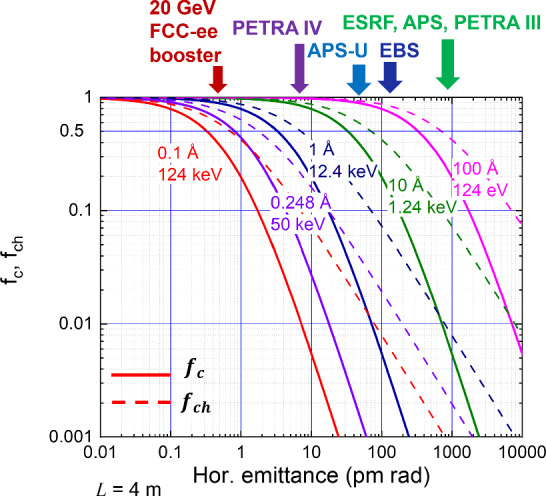
Coherence factors $f_c$ and $f_ch$, defined in Eqs. (5) and (6), for different wavelengths or photon energies as a function of the horizontal emittance with $L=$4 m; emittances of different light sources are indicated by the arrows at the top, including the FCC-ee booster on the left [9]

Figure 14 illustrates the exquisite coherence of photons from the FCC-ee booster down to wavelengths of 0.1 Å, or up to photon energies of 100 keV.

#### Space-charge limit

The case for a storage-ring light source with beam energies of 20 GeV has been made in Ref. [15], where I. Agapov and S. Antipov discuss limitations from space charge and intrabeam scattering (IBS). The authors conclude that “achieving further significant emittance reduction and increase in radiation brightness is only possible by increasing the beam energy”.

The transverse emittances in the booster, especially with strong additional damping wigglers, can shrink to values so low that, also for the FCC-ee, the space-charge force becomes significant. The vertical space-charge tune shift is [16]

7 $$\begin{aligned} \Delta Q _{\textrm{SC}, y} \approx \frac{N_b r_e C}{(2 \pi )^{3/2} \gamma ^3 \sigma _z} \; \left\langle \frac{{\beta _{y}}}{ \sigma _y \sigma _x }\right\rangle \; \approx \frac{N_b r_e C}{(2 \pi )^{3/2} \gamma ^3 \sigma _z \varepsilon _x \kappa ^{1/2} \left( 1+\kappa ^{1/2}\right) } \; , \end{aligned}$$

where $\kappa = \varepsilon _y/\varepsilon _x$. Table 8 shows that the space charge tune shift is about a quarter of an integer already for the equilibrium emittance of the “$3\,U_0$”case, where wigglers increase the natural damping rate by a factor three. For the more extreme $94\,U_0$ case, the space charge effects would be prohibitive in this ring configuration, so that mitigation measures are needed.

Therefore, to support photon science applications with beams of ultra-low emittance, obtained by large additional damping, the booster ring may need to operate with full coupling, $\kappa \approx 1$, at least in the arcs, similar to what had been proposed for the TESLA damping rings [17], if not over the entire ring, in which case the horizontal beam emittance at the undulators could be halved. In addition, harmonic cavities would further lower the space-charge tune shift, typically by a factor of two [16]. A final mitigation could be reducing the bunch charge.

**Table 8:** Estimated space-charge tune shifts in PETRA IV, SOLEIL II, and the FCC-ee booster in equilibrium at injection energy (see Table 6)

	PETRA IV	SOLEIL II	FCC-ee booster ($3\times U_0$)
Beam energy [GeV]	6.0	2.75	20
Circumference [km]	2.305	0.354	90.7
Max. bunch charge [nC]	8	7.4	4
RMS bunch length [mm]	20	15	4
RMS horizontal emittance $\varepsilon _x$ [pm]	20	83	15
RMS vertical emittance $\varepsilon _y$ [pm]	2	8	1.5
Emittance ratio $\kappa$	0.1	0.1	0.1
$\Delta Q_{\textrm{SC}, y}$	0.057	0.036	0.27

The implications of intrabeam scattering and Touschek effect for the ultralow-emittance beams at 20 GeV will also need to be examined.

### Imaging opportunities at 50–100 keV

Hard X-rays with photon energies in the 50–100 keV range will enable novel ways of exploring materials properties and new scientific investigations, as explored at a recent workshop [18].

High-energy X-rays are perfect for imaging thicker samples or objects with high atomic numbers because of their deep penetration capabilities. They also minimize radiation damage, preserving delicate materials and biological samples. Furthermore, high-energy X-rays offer superior contrast for dense materials, resulting in clearer and more detailed images. Using phase-contrast imaging at high energies is essential as it enhances the visualisation of subtle density variations that traditional absorption techniques miss. Coherent sources are vital because they provide the spatial coherence needed for high-resolution phase-contrast images. Physically, coherent sources allow for the observation of weak wavefront perturbations, improving contrast and sensitivity to internal structures. In addition, coherent X-ray sources enable the study of dynamic processes and fine interference structures, which are crucial for understanding the detailed behaviour of complex materials.

**Fig. 15:**
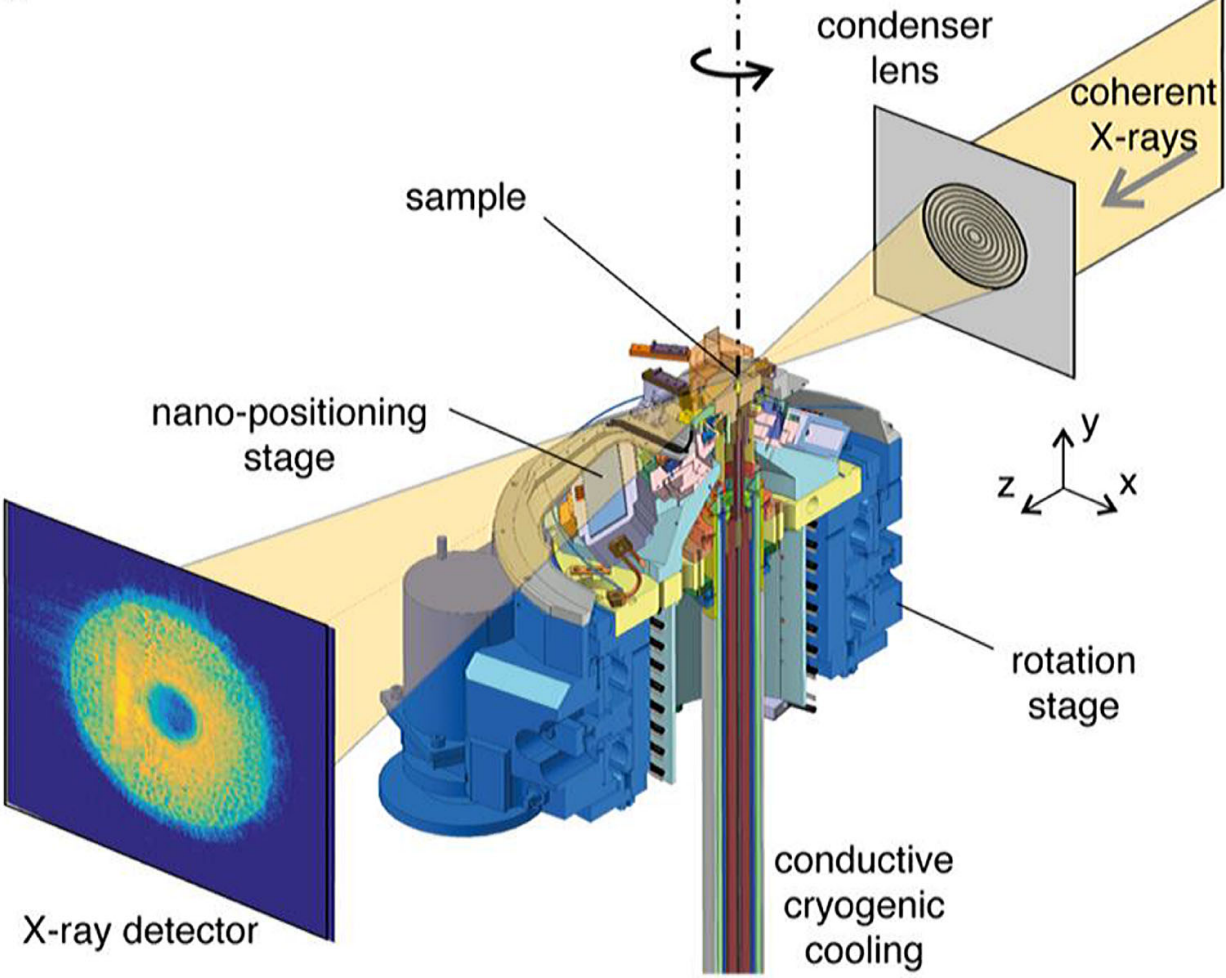
Ptychography setup; top right: incoming coherent beam is focused by a lens to define the illumination on the sample; center: cryogenic stage for scanning with 10 nm accuracy with rotation capability; bottom left: example diffraction pattern recorded by the X-ray detector [19]. Picture taken from Ref. [20]

The high intensity and short wavelength available at an FCC-based light source are expected to significantly extend the range of possible imaging and achievable resolution. The increased brilliance, approximately two orders of magnitude beyond fourth-generation light sources, would be a key factor for time-resolved imaging. Figure 15 shows a ptychographic imaging scheme currently typically implemented at the latest-generation synchrotron facilities.

The resolution for images of soft matter and biological materials is ultimately limited by structural modifications induced by the high energy of short-wavelength radiation. Imaging inelastically scattered X-rays at a photon energy of 60 keV provides a higher signal per energy transferred to the sample than coherent-scattering techniques like ptychography and projection holography, thus potentially offering a complementary approach.

**Fig. 16:**
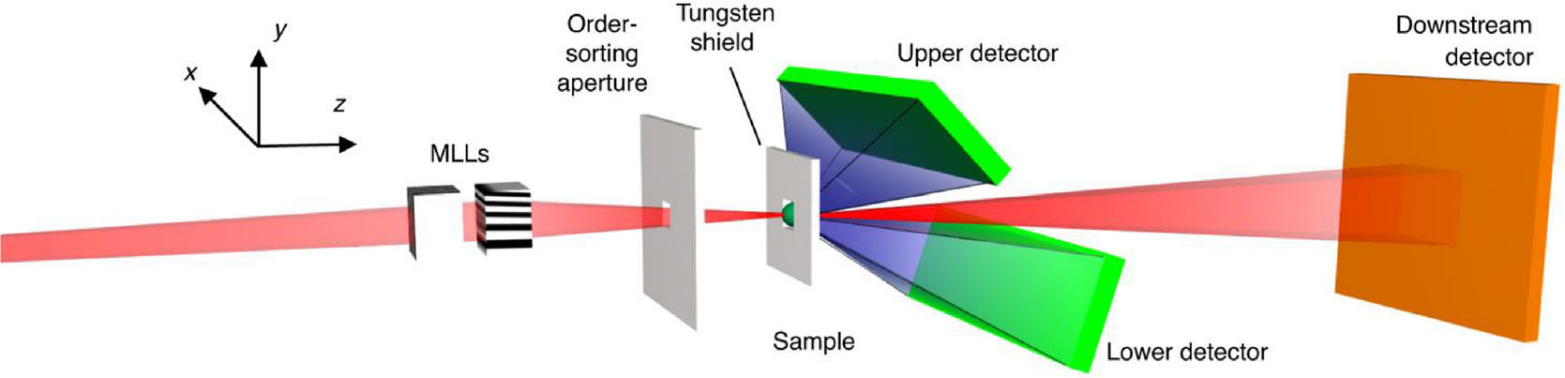
Scanning-Compton Imaging setup as described in [21], taken from Ref. [20]

Compton scattering requires high-energy photon sources with and without circular polarisation. Scanning Compton Imaging (SCI) has recently been demonstrated at PETRA III with 60 keV photon energy, achieving a resolution of approximately 70 nm [21]. Figure 16 shows a diagram of the experimental setup used at PETRA III. The PETRA III measurements were limited by the relatively low brightness of the X-rays at 60 keV. PETRA IV will have about 1000 times higher brightness at 60 keV, resulting in a resolution improvement by up to a factor of 10 (depending on the sample object) [22]. The brightness at the FCC-ee in this photon energy range would be at least another two orders of magnitude higher than at PETRA IV (see Fig. 11), rendering both the FCC-ee booster and the HE linac nearly ideal drivers for such a facility. At FCC-ee, a resolution of a few nm can be expected. Additionally, the relatively low radiation dose required for SCI is an advantage, as higher photon energy reduces the absorption cross section faster than the Compton scattering cross section.

**Fig. 17:**
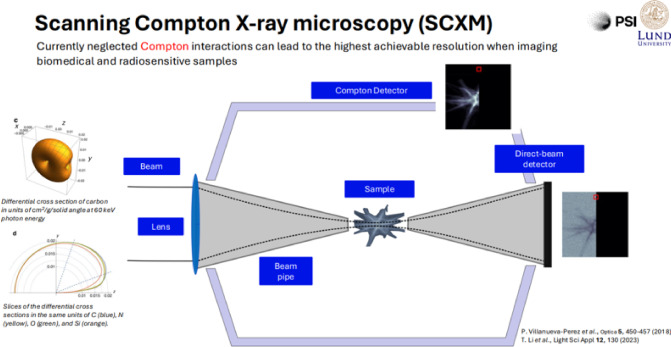
Concept of Scanning Compton X-ray Imaging, taken from Ref. [20]

The exceptional peak brilliance and coherent fraction at FCC-ee will also enable high-energy, time-resolved ptychographic imaging, facilitating the examination of larger samples, heavier materials, and in-situ/operando conditions. Additionally, the outstanding average brilliance will advance dynamic imaging beyond current capabilities, ultimately establishing Scanning Compton X-ray Microscopy (SCXM) as a valuable tool, see Fig. 17.

**Fig. 18:**
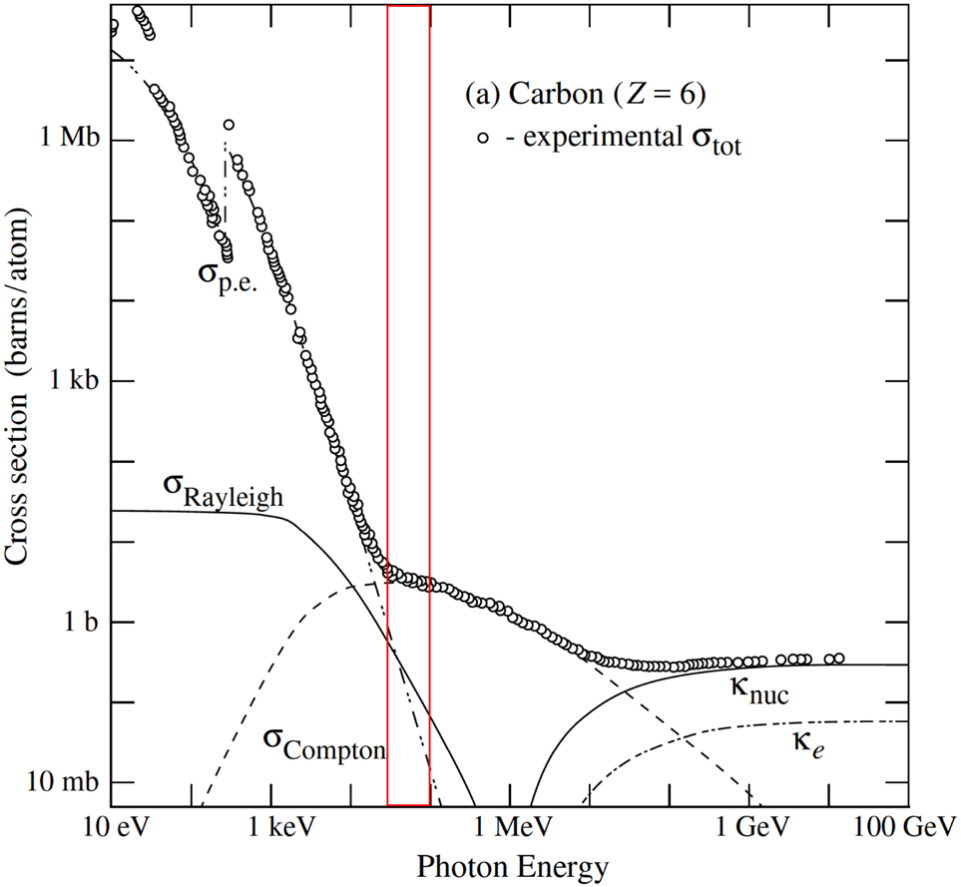
Total photon cross section for $^{12}$C, from [23]. The red box indicates the photon energy available at high brilliance at an FCC-ee photon facility

Figure 18 impressively illustrates how, above 30 keV X-ray energy, the Compton window in $^{12}$C opens up, extending to about 10 MeV when pair production in the nuclear field starts to dominate. This is particularly relevant for imaging radiation-sensitive biological materials. For heavy nuclei like Pb, the window narrows at higher energies, but even in such extreme cases, there remains an energy region around 1 MeV where Compton scattering is the dominant process.

The FCC-ee injector linac and the FCC-ee booster at 20 GeV injection energy can both produce radiation with a spectrum reaching 100 keV and beyond. Such photon beams will allow the exploration of new frontiers in various scientific areas [24] like

- Applied Materials and Industrial Applications
- Structural Dynamics in Disordered Materials
- Dynamics of Functional Materials
- High Pressure, Planetary Science, Warm Dense Matter, Relativistic Laser Plasma, Strong Field Science

Such a facility would directly address technical limitations arising from a lack of diagnostics with sufficient bulk sensitivity, along with the required spatial and temporal resolution.

An MEC (Matter under Extreme Conditions) experimental area would address and extend studies of high-pressure effects with applications to planetary science and geology, electron dynamics and dense-matter properties.

Methods and instrumentation may take advantage of the following features:

- Larger penetration depth for bulk sensitivity, and access to complex sample environments
- Larger momentum transfer at moderate scattering angles for improved accuracy in modelling, for inverse analysis schemes, and for crystallography of small unit cell materials
- Access to core-level spectroscopy of heavier elements and nuclear resonances
- Reduced radiation damage for high repetition rate tracking of induced (pumped) dynamics
- Imaging stochastic phenomena in heterogeneous samples
- New techniques (e.g. Compton scattering)

A photon source at up-to 100 keV X-ray energy and beyond could also open a new approach to strong-field science by reducing the electron-beam energy necessary to reach the required electric fields to more manageable proportions. There is a significant interest in pushing the EuXFEL towards harder X-rays, which is indicative of the excitement a hard X-ray FEL at the FCC injector linac or high-energy photons from the FCC-ee booster ring could generate.

### Compton scattering imaging with photons above 100 keV

High-resolution and magnetic Compton scattering using high-energy X-rays from a synchrotron is an advanced form of inelastic X-ray scattering. This technique involves energy transfers exceeding 10 keV and momentum transfers sufficient to differentiate between core and valence electrons. Consequently, it enables the measurement and visualisation of electron momentum distribution in materials [25].

Compton scattering at synchrotron radiation sources provides unique insights into the Fermi surface of materials that are inaccessible by other techniques, such as ones based on the de Haas–van Alphen effect, e.g. Refs. [Phys Rev 144, 39], [26]. It offers bulk sensitivity and does not necessarily require good electrical conductivity. Developing a bent crystal optics is essential for achieving good energy resolution, and access to circularly polarised light is also necessary. Circularly polarised radiation allows distinguishing different contributions to electronic orbital occupancy in alloys, a method known as magnetic Compton scattering.

Compton scattering requires X-ray energies above 50–100 keV. As a non-resonant technique, it allows access to the ground state electronic properties of correlated electron systems and is applicable to metals, semiconductors, and superconductors. At high energies, exceeding 100 keV, X-rays can penetrate, for example, a working electrical coin battery while in operation, and the resulting position–time intensity map allows unveiling lithium migration and structural changes due to electrode volume expansion [27]. In magnetic materials, spin-dependent electron distributions can be mapped. This non-destructive and bulk-sensitive method renders it a unique spectroscopic tool for condensed matter physics, solid-state chemistry, and material science.

### “Gold-plated” photon applications with photon energies at 5–20 MeV

Photons produced by the FCC-ee booster at 45.6 GeV, or 182.5 GeV, would allow a study of *pygmy resonances*, at photon energies between 5 and 10 MeV, which is considered a “gold-plated” application.

**Fig. 19:**
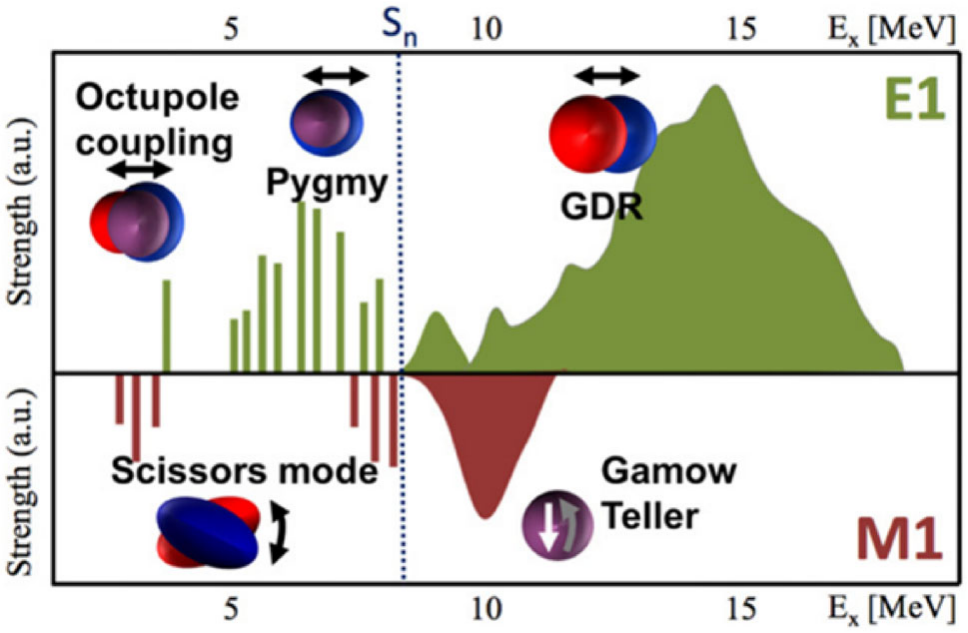
Diagram of the landscape of the response of nuclei to photon absorption [29]

Pygmy Dipole Resonances (PDR) [28, 29] are low-energy electric dipole (E1) excitations observed in neutron-rich nuclei, appearing below the neutron separation energy, as is illustrated in Fig. 19. They represent a small fraction of the total dipole strength, in contrast to the much stronger Giant Dipole Resonance (GDR). PDRs are characterised by the oscillation of the neutron-rich outer “skin” against the proton–neutron core, with contributions from both isoscalar and isovector components. This unique structure allows them to be studied using various experimental probes. Their significance extends beyond nuclear structure studies, offering insights into neutron behaviour at the nuclear surface and the nature of nuclear forces. In astrophysics, PDRs play a key role in modelling neutron-capture rates in the r-process, which is responsible for the formation of heavy elements in the universe.

The FCC-ee light source would enable the world’s first PDR measurements, based on photon absorption. Also the energy of the giant dipole resonances, around 10–20 MeV, would be within reach, The booster could be run for photon production, in-between cycles during the higher energy running modes of the FCC-ee collider. Alternatively, this photon energy might also be achieved, and more easily so, in steady state, from an undulator in the collider itself when running at $\textrm{t}\bar{\textrm{t}}$ energies.

### Photons beyond 10 GeV: laser Compton scattering in the collider

**Fig. 20:**
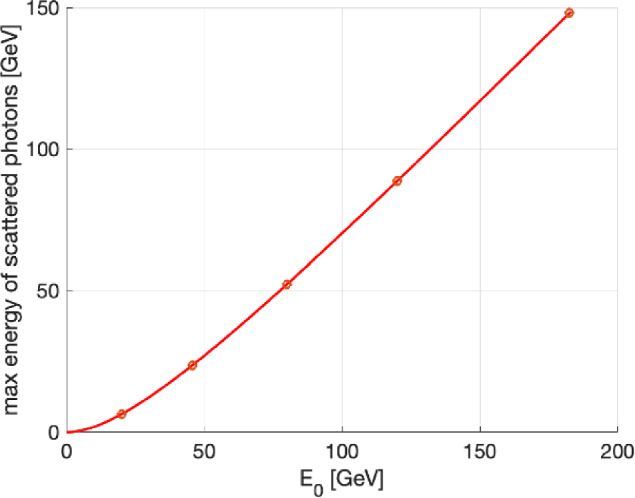
Energy of scattered photons as a function particle energy, considering a Ti:Sa laser with $\lambda \approx 800$ nm

Compton Back Scattering (CBS), also called Inverse Compton Scattering (ICS), is a process in which a photon collides with a charged particle, such as an electron or positron, and is scattered into a different direction with a changed wavelength or energy. During this process, energy is exchanged between the photon and the charged particle, resulting in modifications to their respective energy states. The photon energy range attainable at the FCC is illustrated in Fig. 20.

In the FCC-ee, CBS (or ICS) is utilised in several key processes, including beam diagnostics via a Compton polarimeter, beam intensity control [30], and as a potential gamma-ray source at the booster. Among these applications, beam intensity control and the potential gamma-ray source at the booster are of particular interest in the context of other scientific opportunities at the FCC-ee, due to the produced photons, which can be utilised in various scientific experiments and applications. The novel domain of Full Inverse Compton Scattering with Unruh photons is discussed in Sect. 4.4, and various applications of CBS at the FCC-ee to strong-field quantum electrodynamics (QED) and high-energy physics and are explored in Sects. 4.2 and 4.2.1, respectively.

**Fig. 21:**
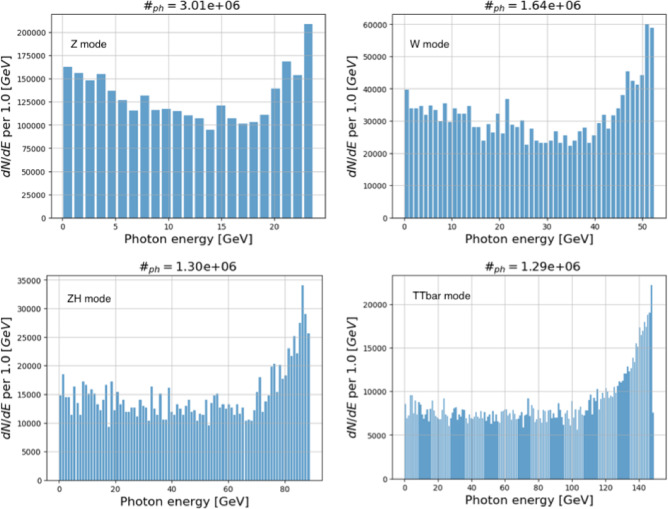
Simulated energy spectrum of scattered photons for different FCC-ee operation modes (top left: Z pole; top right: WW threshold; bottom left: ZH production; bottom right: above $\textrm{t}\bar{\textrm{t}}$ pair production threshold), considering electron collisions with a 50 $\mu$J Ti:Sa laser pulse (laser wavelength around 800 nm)

Beam intensity control via CBS is developed to equalise the charge of the beams, thereby eliminating effects such as beam-beam flip-flop instabilities. This is achieved through the high recoil experienced by particles with high energy during CBS interactions, using a 50 $\mu$J Ti:Sa laser pulse (laser wavelength around 800 nm). As a by-product, high-energy photons are produced. The expected photon flux is approximately $10^{10}$ photons per second, with photon energies ranging from 24 GeV in the Z operation mode to 150 GeV in $\textrm{t}\bar{\textrm{t}}$ operation mode. The spectra of these photons are presented in Fig. 21.

In contrast to CBS used for beam intensity control, where the number of scattered photons depends on the collider’s operating mode and beam quality, an alternative proposal explores expanding the application of CBS by adding an additional interaction point in the booster to serve as a light source.

This proposal involves the installation of a Fabry-Perot resonator, enabling laser photons to collide with beams in the booster at a high repetition rate of approximately 37 MHz. Such a high repetition rate could achieve a photon flux of up to $10^{11}$ photons per second by interacting with only $10^4$ particles in a bunch populated by $10^{11}$ particles. This scheme minimises the adverse effects of scattering on the quality of particle bunches in the booster, ensuring a stable and predictable photon flux.

These high-energy photons, particularly those with energies up-to 150 GeV, hold significant potential for both particle physics and astrophysics. In particle physics, they enable detailed studies of fundamental interactions, heavy particles, and physics beyond the Standard Model. In astrophysics, they enhance our understanding of cosmic phenomena, including gamma-ray bursts and cosmic rays. More details and other applications are discussed in the following section.

### Science with photons at 10 s of GeV

A Compton backscattering source based on the FCC-ee booster, FCC-CPS, could produce significantly larger photon flux, and at much higher photon energy, than ELI-NP.

As is illustrated in Table 9, the photon energies at FCC-CBS are 1000 times higher, the photon flux exceed ELI-NP’s also by at least a factor 1000. To achieve this rate, for FCC-ee, the laser beam recirculator system of ELI-NP would need to either be modified or be replaced by an optical cavity, suitable for cw operation.

**Table 9:** Comparison of ELI-NP and FCC-ee Compton Backscattering Source (FCC-ee-CBS), assuming Yb:YAG laser (2.3 eV)

Facility	ELI-NP	FCC-ee-CBS-20	FCC-ee-CBS-45	FCC-ee-CBS-120
Beam energy [GeV]	0.72	20	45.6	120
Average beam current [A]	$0.8\times 10^{-6}$	0.015	0.015	0.005
Beam size at laser CP [mm]	$\sim 0.5$	$\sim 0.5$	$\sim 0.5$	$\sim 0.5$
Compton *x* parameter	0.025	0.7	1.6	4.2
Max photon energy [GeV]	0.02	8.3	28	97
Photon flux [1/s]	$10^{9}$	$\sim 10^{12}$	$\sim 10^{12}$	$\sim 10^{12}$

Photons at energies up to 100 GeV could be used for nuclear physics, hadron physics, and QCD explorations. For example, these photons could serve to “see” the QCD confinement mechanisms at work, through the production of exotic mesons (glueballs, etc., ...); and the exchange of, free or bound, partonic systems [31]. At lower energies, SPring-8 operates a facility generating GeV $\gamma$-rays, via inverse Compton scattering. Photons in an energy range of 1.5–2.4 GeV are used to study the sub-nucleonic structure of nucleon via the productions of the $\phi$ and K$^+$ mesons [32]. The exclusive photoproduction of a photon-meson pair is a promising channel to study quark generalised parton distributions (GPDs) [33]. In addition to probing quark GPDs, this process allows extracting chiral-odd GPDs at the leading twist, by choosing the outgoing meson to be a transversely polarised $\rho$-meson [33].

Presently most advanced in this domain are the facilities at JLAB. The real photon beam in JLAB Hall D ($\sim$10 GeV) is being used for spectroscopy in search of exotic mesons (hybrid states) and near-threshold charm (J/$\psi$) production to study the gluon field contribution to the proton mass, so-called “trace anomaly”, which has been a hot topic in recent years. The JLab upgrade to 22 GeV would yield a real photon beam at $\sim$20 GeV; the pertinent physics case was highlighted in the “JLab22” White Paper [34].

The photon energy of 40–120 GeV range could cover, in addition to having much wider phase space for charmonium, the beauty quarkonium and near-threshold Upsilon productions (cleaner than the J/$\psi$ case and one of the main goals of the Electron-Ion Collider physics [35]). Experiments with such high-energy photon beams would make an important impact on the study of the gluon field contributions to the proton mass. In addition, circularly polarised photon beams would enable measuring the polarised gluon distribution, testing the convergence of the polarised sum rule (GDH sum rule), and helping constrain the high energy behaviour (the Regge theory parameters) [35].

Another application of high-energy Compton backscattering could be demonstrating the direct scattering of light off light, after indirect observations in ultra-peripheral heavy-ion collisions at the LHC [36]. Colliding (counter-propagating) photons in the energy region just below the e$^+$e$^-$ production threshold ($\sim 500$  keV) enables a study of the elastic photon-photon collisions (box diagram physics). The small cross section for this process calls for high-finesse Fabry-Perot cavities to enhance the flux. So, one option could be scattering 40–60 GeV photons off $\sim 1$ eV photons from a laser, stored in an optical cavity. More intriguingly, scattering 63 GeV photons off 63 GeV photons would allow producing Higgs bosons, $\gamma \gamma \rightarrow \textrm{H}$. The theoretical total cross section for light-off-light scattering from 1 MeV to 1 TeV can be found in the literature [37]. The total cross section decreases from about 1 $\mu$barn at a few MeV to $\sim$10 fb at 100 GeV, followed by a peak of the cross section corresponding to $\gamma \gamma \rightarrow$ H Higgs production of $\sim$200 fb around 125 GeV [38].

The ThomX facility at IJCLab could serve as a test bed for CBS beam dynamics in a ring and explore three different regimes: (1) ring dominated with laser as a perturbation, (2) laser ring regime (Thom-X design), and (3) nonlinear laser-interaction with higher harmonics.

### Photons from coherent bremsstrahlung in oriented crystals

High energy and high intensity photon beams are playing an important role in the investigation of matter. For these purposes, dedicated photon-source lines with high intensity and energy are constructed. The generation of high energy photon beams in the GeV region is complicated since photons cannot be accelerated in the same way as charged particles. Instead, we need to produce them at the desired energies: GeV region in our case.

One effective method for producing high energy photon beams is the direct interaction of charged particles with target materials, which gives rise, e.g. to bremsstrahlung of electrons in materials. The energy spectrum of such photons extends from low energies up to almost the initial electron energy. Thus, any part of the energy spectrum of the photons can be selected as a desired photon beam.

The produced photon beams are characterised by several parameters, such as the photon yield, spectral distribution, beam intensity, angular distribution and beam polarisation. It is necessary to choose, from these parameters, the optimal photon beam for a particular experiment.

The bremsstrahlung radiation of electrons in amorphous media has several disadvantages as a photon source. One of the weaknesses of this method is the low intensity of the produced photons at high energies. The generated photon beam is not polarised and the angular distribution is rather large, $\sim 1/\gamma$, where $\gamma$ is the Lorentz factor.

The nature of the radiation is dramatically changed when the charged particles traverse a crystalline media at relatively small angles with the crystallographic axes or planes. In case of a periodic medium the radiation cross section is not just the sum of cross sections from separate atoms. An electron impinging on a crystal will interact coherently with atoms in the aligned crystal axes or planes. If the Laue condition is satisfied, coherent radiation phenomena occur [39]. The essential characteristics of the coherent radiation are quasi-monochromatic spectrum, high intensity, a small polar angle distribution and high linear polarisation degree of radiation in the coherent maximum.

The classical theory of Coherent Bremsstrahlung (CB) is discussed in Ref. [39]. The classical results of CB of relativistic electrons in a crystal were obtained on the basis of the first Born approximation of QED. The validity conditions of the first Born approximation are quickly broken with increasing particle energy (tens and hundreds of GeV) and with decreasing angles of incidence relative to the crystal axes or plane. The particle radiation in a crystal for a broad range of energies and angles can be treated in the semiclassical approximation [40, 41]. The formulas of CB are presented in a convenient form for performing calculations in Ref. [42].

**Fig. 22:**
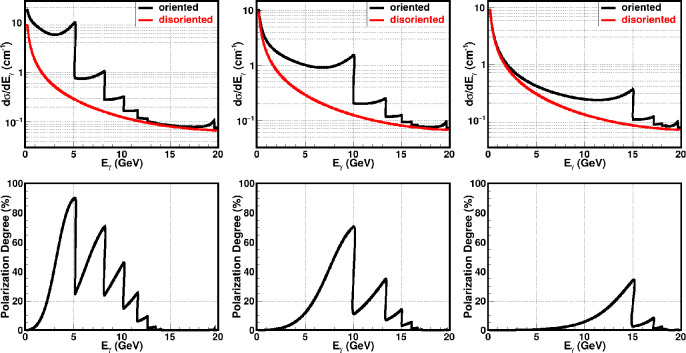
Upper plots: Calculated coherent (black) and incoherent (red) radiation cross sections for different $\psi _0$ angles. Bottom plots: Calculated polarisation degree of coherent radiation in diamond crystal for the following $\psi _0$ angles: $\psi _0 = 0.23\,$mrad, $\psi _0 = 0.67\,$mrad, $\psi _0 = 2.0\,$mrad

Differential cross section of coherent radiation in a crystal is composed of two terms [39] coherent and incoherent bremsstrahlung:

8 $$\begin{aligned} d \sigma = d \sigma _{inc} + d \sigma _{coh} \end{aligned}$$

The first term corresponds to the incoherent cross section (including radiation in the field of the atomic nucleus and electrons) from the interaction with *N* independent atoms. The second one corresponds to the coherent radiation cross section. Following Ref. [42], let us denote ($E_0, \textbf{P}_0$), ($E_1, \textbf{P}_1$) and ($E_{\gamma }, \textbf{P}_{\gamma }$) as the energy and momentum of incoming, outgoing electrons and emitted photon, respectively. The initial electron beam orientation with respect to the crystal axes is defined by two angles in the following manner. The three chosen orthogonal axes of the cubic crystal are $[\mathbf {b_1 \, b_2 \, b_3}]$. The orientation of the primary electron beam is defined by the angle $\theta _0$ between the initial electron momentum $\textbf{P}_0$ and the crystal axis $\textbf{b}_3$ and by the angle $\psi _0$ between the electron momentum and the crystal plane $(\mathbf {b_1 \, b_3})$. Let $\theta$ and $\varphi$ be the polar and azimuthal angles of the emitted photon with respect to the initial direction of motion of the electron.

Suppose an electron with energy $E_0 = 20$ GeV is incident at $\theta _0 = 40~$mrad with respect to the <001> axis of a diamond single crystal. The CB radiation cross sections are given, in Fig. 22, for three different $\psi _0$ angles from the (110) plane: $\psi _0 = 0.23\,$mrad, $0.67\,$mrad, $2.0\,$mrad. One can see that it is possible to vary the coherent peaks by changing the angle $\psi _0$ with respect to the (110) plane. The plot illustrates the enhanced radiation spectra of CB radiation compared with the incoherent radiation.

The produced photons by CB are linearly polarised even though the initial electron beam is unpolarised. The calculated linear polarisation degrees of the produced photons for different $\psi _0$ angles are presented in Fig. 22 (bottom plots). The maximum peak intensity and maximum polarisation occur at the same photon energy. This energy can be selected by choosing the orientation of the crystal lattice planes with respect to the incoming electron beam. The polarisation of the produced photons can be up to 90 % depending on the maximum intensity peak position. The coherent processes are diminishing with increasing angle of incidence of the primary particle, as can be seen in the bottom pictures of Fig. 22.

The production of circularly polarised photon beams by unpolarised electron beams seemed to be difficult, if not impossible. It was shown in Ref [43] that the bremsstrahlung photons emitted from longitudinally polarised electrons can be circularly polarised due to the helicity conservation.

**Fig. 23:**
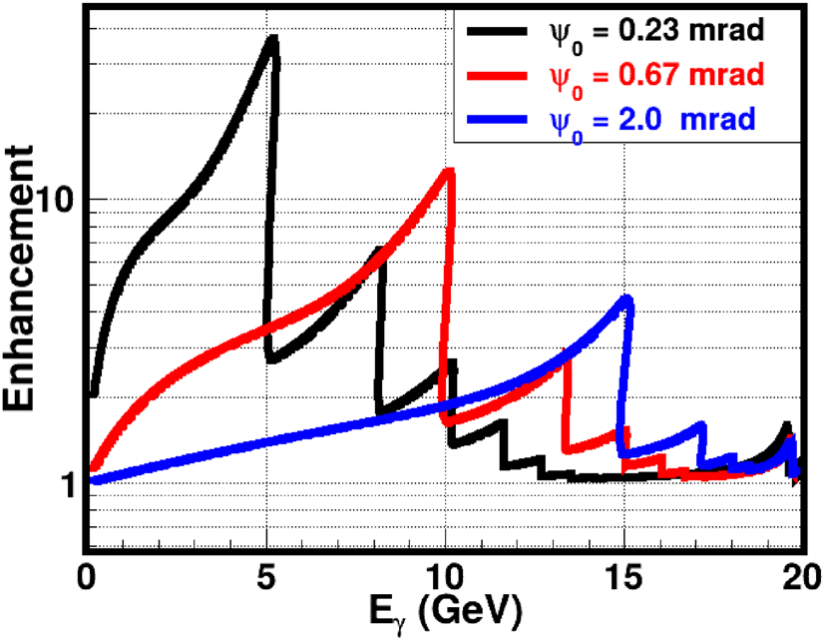
Enhancement of coherent radiation in diamond crystal for different $\psi _0$ angles

The increase in the CB radiation intensity spectrum is usually reported with respect to the incoherent bremsstrahlung spectrum. This ratio, called the enhancement, is shown in Fig. 23 for three different $\psi _0$ angles.

**Fig. 24:**
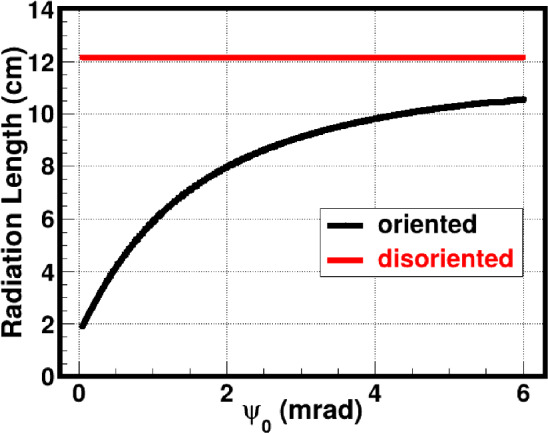
Radiation length of oriented and disoriented diamond crystal as a function of the $\psi _0$ angle

The important quantity characterising the shower development in matter is the radiation length of the charged particles. In amorphous media, this length only weakly depends on the particle energy, so that the radiation length is practically constant for a given amorphous material. In case of a crystal, however, the radiation length strongly depends on the crystal type, its orientation, the particle energy, and the particle polarisation. The dependence of the radiation length on the angle of incidence is shown in Fig. 24. For an oriented crystal the radiation length is much shorter than for an amorphous medium. With increasing incident angle the radiation length is increasing; it asymptotically approaches the amorphous value.

**Fig. 25:**
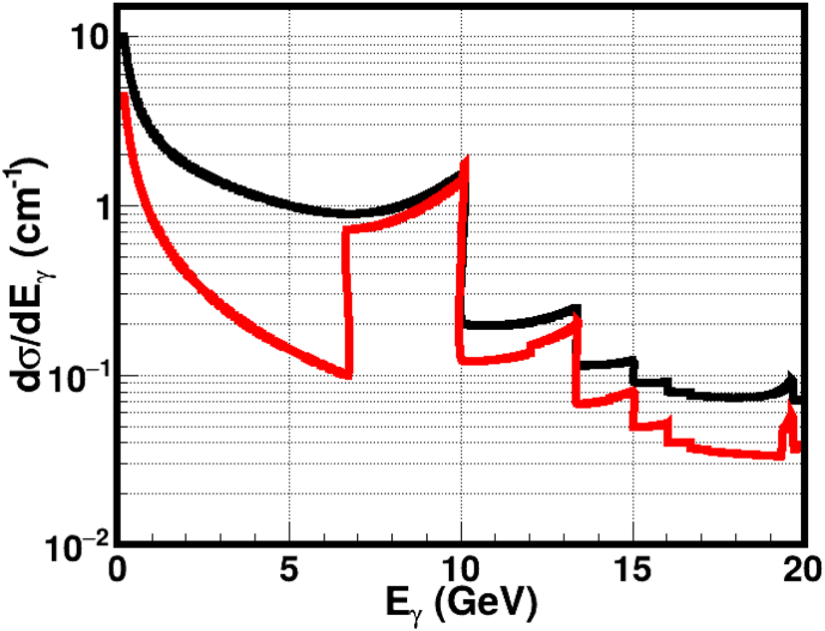
Distribution of produced photons by CB without collimation (black) and when collimated at an angle of $25\,\mu$rad (red)

As was mentioned above, the emission angle of incoherent bremsstrahlung photons is confined to $\sim 1/\gamma$. For example, with 20 GeV primary electrons, the RMS photon emission angle is $\sim 25\,\mu$rad. In case of CB, there is a strong correlation between the energy of the coherent peaks and the angle of photon emission with respect to the incident electron direction. By collimating the photon beam, one can eliminate photons beyond a certain angle, and narrow the width of the peaks in energy, as is illustrated in Fig. 25 for a collimation angle of 25 $\mu$rad.

A Monte Carlo simulation code has been developed and tested for studying the passage of charged particle beams and radiation through the crystalline matter at energies extending from tens of MeV up to hundreds of GeV based on the formulae in [41, 42]. This Monte Carlo code was successfully used for the prediction of the results of CERN-NA59 experiment [44, 45].

**Fig. 26:**
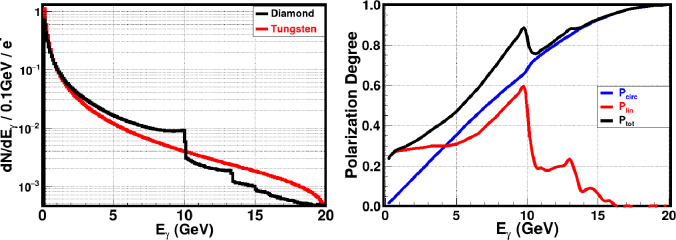
Left plot: Monte Carlo simulation results for the produced photons spectra in case of coherent bremsstralung (black) in an oriented diamond crystal and incoherent (red) radiation in tungsten amorphous materials. Right plot: Expected linear (red, $P_{\textrm{lin}}$), circular (blue, $P_{\textrm{circ}}$) and total (black) polarisation degree of coherent radiation in the diamond crystal. The total polarisation is defined as $P_{\textrm{tot}} = (P_{\textrm{lin}}^2 + P_{\textrm{circ}}^2)^{1/2}$

A series of Monte Carlo simulations has been devoted to the passage of longitudinally polarised electrons through amorphous tungsten and an oriented diamond crystal, using the simulation technique proposed in Ref. [42]. In these simulations, the initial electron beam parameters are chosen to be close to the FCC-ee electron beam parameters. The electron beam energy is taken to be 20 GeV with 0.1% spread. The electron beam emittance was chosen to be 5 nm. More precisely, the RMS angular spread of the electron beam in both *x* and *y* directions is 5 $\mu$rad and the spatial RMS size is 1 mm. For the purpose of illustration, we assume that the initial electron beam is 100% longitudinally polarised. The thickness of both targets was chosen to be one radiation length ($1 X_0$). The corresponding thickness of the amorphous tungsten is 3.5 mm. In case of the oriented diamond crystal, we consider an electron beam incident at $\theta _0 = 40$ mrad from the <001> axis of diamond single crystal and $\psi _0 = 0.67$mrad from the <110> plane. Under these conditions, the thickness of the diamond target, equivalent to one radiation length, is equal to 4.82 cm, according to Fig. 24.

The emitted photon spectra in amorphous tungsten and oriented diamond crystal are shown in Fig. 26 (left). A large enhancement of CB photons can be seen around the coherent peak between 4 and 10 GeV. The circular, linear and total polarisation degrees of the produced photons are shown in the right plot of Fig. 26. The advantage of the CB over the incoherent bremsstrahlung in amorphous materials can be seen. The results confirm that by replacing the amorphous targets by crystals, one can increase the photon beam intensity in the high energy range. The produced photons have a high degree of linear polarisation near the coherent peak (red colour). The produced photons also have a high degree of circular polarisation if the initial electron beam is longitudinally polarised (blue). It is possible to shift the energy range of the coherent peak by changing the orientation angles of the crystal. The use of oriented crystals as radiators provides the possibility of producing high energy photon beamlines with high intensity, tunability, monochromaticity and polarisation.

The circularly polarised intense photon beams produced by CB in oriented crystals can also be used to generate polarised positrons. Longitudinally polarised positrons are created as a result of the interaction of the circularly polarised photons with an amorphous or crystalline target. In addition, replacing an amorphous positron target by an oriented crystal enables enhanced, coherent electron-positron pair production.

### Crystalline undulators: a gamma-ray source for the FCC-ee linac positron beam

#### Overview of light sources and undulator radiation

The development of light sources (LS) capable of operating at sub-Ångstrom wavelengths remains one of the foremost challenges in modern physics. High-brilliance, tunable LSs are indispensable for a broad range of applications, including particle physics, photo-nuclear research, nuclear structure studies, solid-state physics and medical imaging [46]. Current LS, such as X-ray Free Electron Lasers (XFELs), face significant challenges in reaching wavelengths below the Ångstrom scale due to the demanding advancements required in both magnet and accelerator technologies. While synchrotron sources are capable of reaching shorter wavelengths by using proper insertion devices, they suffer from a significant reduction in intensity as the wavelength decreases [46]. An innovative approach to overcome these limitations involves the generation of gamma-rays through inverse Compton scattering (ICS). The ICS radiation can be very hard (up to hundreds of MeV) and intense [47]. However, this method requires sophisticated particle accelerators and advanced laser systems, making it resource-intensive and hard to implement.

**Fig. 27:**
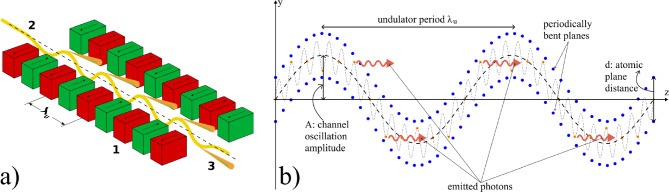
**a** Magnetic undulator, **b** layout of a crystalline undulator, adapted from [48]

A promising alternative is provided by crystalline undulators (CUs). This approach utilises the unique properties of crystals to confine electrons or positrons through a phenomenon known as channeling [49]. When charged particles traverse a crystal at an angle smaller than the Lindhard critical angle,

$$\theta _c = \sqrt{\dfrac{2U_0}{E}},$$

where *E* is the particle energy and $U_0$ is the amplitude of the average potential energy, they undergo coherent interactions with the crystal atoms. This results in an oscillatory motion, either near or away from atomic strings forming the crystal planes, depending on the particle’s charge [49]. The efficiency of this process is significantly higher for positron beams, as positrons oscillate between crystal planes and interact less with atomic nuclei and surrounding electron clouds. This oscillatory motion is intrinsically linked to the emission of electromagnetic radiation known as Channeling Radiation (ChR). In the energy range of few tens of GeV for the drive beam, the ChR is typically softer but considerably more intense than both incoherent and coherent bremsstrahlung radiation. For example, at 20 GeV, the ChR spectrum for electrons and positrons ($e^{\pm }$) generally falls within the hundreds of MeV range, depending on the specific crystal parameters.

A crystalline undulator (CU), is a crystal to which a periodic (ideally sinusoidal) deformation is imparted to generate intense monochromatic electromagnetic radiation [50]. First conceptualised in 1980 [51], the idea has been further developed over the past decades, especially by the MBN group [48, 52–54]. The CU mechanism relies on the strong crystalline interplanar electrostatic field ($\sim 10^9$ V/cm) to replace the role of magnets in driving particle oscillation, as depicted in Fig. 27. Compared to magnetic undulators, where the period of oscillations $\lambda$ is limited to about 1 cm up to now, CUs can achieve sub-mm periods, enabling the generation of undulator radiation (UR) composed of harder photons. This radiation is inherently more monochromatic and, as shown below, can be significantly more intense than ChR. Moreover, theoretical studies suggest that under specific conditions – such as an appropriately modulated incoming positron beam – a crystalline undulator could operate in a stimulated emission regime (lasing effect), leading to an enhanced coherence gain in the emitted photons [46, 50].

The energy of the $\gamma$-rays emitted by an undulator is given by:

9 $$\begin{aligned} E_\gamma = \frac{4 \gamma ^2 \pi \hbar c}{1 + \gamma ^2 \vartheta ^2 + \frac{K^2}{2}} \cdot \frac{1}{\lambda _u}, \end{aligned}$$

where $\gamma$ is the Lorentz factor of the beam impinging on the CU, $\vartheta$ is the emission polar angle, and *K* is the undulator strength parameter. For magnetic undulators the parameter *K* is given by

10 $$\begin{aligned} K = \frac{e B \lambda }{2 \pi m_e c}, \end{aligned}$$

where *B* is the peak magnetic field.

By contrast, for CUs one has

11 $$\begin{aligned} K = \sqrt{K_u^2 + K_{ch}^2}, \end{aligned}$$

where

12 $$\begin{aligned} K_u = \frac{2 \pi \gamma A}{\lambda }, \end{aligned}$$

with *A* being the amplitude of the sinusoidal deformation of the crystal, and $K_{ch}$ is the contribution due to channeling oscillations. The latter can be estimated by averaging the channeling oscillation amplitude for different particles and inserting this average value in a formula similar to Eq. (12). For positrons, under the approximation of a harmonic potential, this results in [50]

13 $$\begin{aligned} \langle K_{ch}^2 \rangle = \frac{2 \gamma U_0}{3mc^2}\; . \end{aligned}$$

A CU with a period $\lambda$ on the order of $10^1$ – $10^2 \mu$m can generate MeV photons using a GeV electron beam. In a crystalline undulator, a clear separation between the energy peaks of channeling radiation and the **crystalline undulator radiation** (CUR) can be achieved if the undulator period $\lambda _u$ is significantly larger than the channeling oscillation period $\lambda _{ch}$. This separation can be controlled during the fabrication process. Furthermore, to maximise the intensity of the CUR peak while suppressing ChR, the oscillation amplitude must satisfy the condition $A \gg d_{pl}$, where $d_{pl}$ is the interplanar distance. This optimisation strategy is commonly referred to as the **Large Amplitude Large Period** (LALP) regime [50]. By carefully selecting the parameters of the periodic deformation, it is possible to produce an intense spectral peak over a broad energy range. Additionally, a CU can work with both bunched and continuous beams, offering versatility for different experimental setups.

So far several techniques have been developed to produce crystalline undulators, including the grooving method [55], Pulsed Laser Melting (PLM) [56], ion implantation [57], Si-Ge alternating concentration [58], and acoustically driven deformation [59]. Our approach is based on low-pressure chemical vapor deposition (LPCVD). By depositing an alternating pattern of silicon nitride on a silicon wafer at high temperatures ($\sim 800^\circ$C), a controlled thermal stress is induced during cooling due to the mismatch in thermal expansion coefficients between the film and the substrate [60]. This stress results in the desired periodic deformation, allowing us to fabricate crystalline undulator prototypes. The proposed technique enables the efficient production of multiple samples with a wide range of oscillation parameters. Due to the compact size of CUs, they can be mounted on a mechanical exchange device, allowing for easy variation of the emitted CUR spectrum.

#### Simulations and future prospects

To assess the feasibility of a gamma-ray source based on a CU driven by the positron beam at the exit of the FCC-ee injector, we performed a set of Monte Carlo simulations using the Geant4 [61–63] toolkit embedding the G4ChannelingFastSimModel class [64, 65]. The latter enables the user to consider the coherent interactions of charged particles in crystals and to simulate the related radiation emission processes through the Baier-Katkov method [66, 67]. The features of the positron beam used in our study were taken from Ref. [68] and are reported in Table 10.

**Table 10:** Parameters of the positron beam used in our feasibility study for a gamma-ray source based on a CU fed by the FCC-ee injector

Beam energy	20	GeV
Energy spread	0.5	%
Maximum bunch charge	4	nC
Number of bunches per pulse	4	
Repetition rate	100	Hz
Emittance (x,y)	0.5, 0.05	nm rad
Beam r.m.s. size (x,y)	0.5, 0.05	mm
Beam r.m.s. divergence (x,y)	1, 1	$\mu$rad
Bunch length	2	mm

In our study, we considered a Si crystal and undulation parameters for (110) planes that can be achieved with our manufacturing technique. Namely we considered undulator periods $\lambda _u$ varying from 100 $\mu$m to 1 mm and oscillation amplitudes *A* ranging from 0.2 to 2 nm. The resulting radiation featured a first harmonic spanning from about 2 MeV to about 22 MeV. In particular, here we report the results for a CU with $\lambda _u$ = 200 $\mu$m, *A* = 1.344 nm (7 times the interplanar distance) and a thickness of 3 mm (15 periods). A collimation angle of 26 and 13 $\mu$rad, corresponding to $1/\gamma$ and $1/(2\gamma$), was applied to attenuate the radiation spectrum components outside of the main peak.

**Fig. 28:**
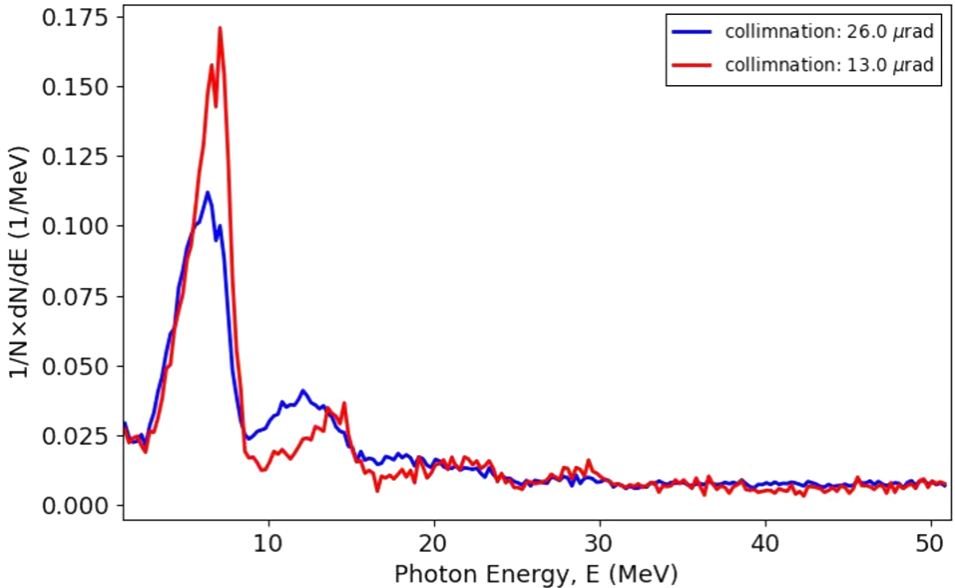
Simulated radiation spectrum for a crystalline undulator with selected parameters and 2 different collimation angles

As shown in Fig. 28, the radiation spectrum with $1/\gamma$ (blue curve, 26 $\mu$rad) collimation features a prominent peak at 6.5 MeV due to undulator radiation accompanied by a 2nd harmonic. The peak brilliance of the first harmonic turns out to be about 2.1$\times 10^{21}$ photons/ (s mm$^2$ mrad$^2$ 0.1%BW), which is in line with previous numerical simulations for other CUs [50, 69–71].

In order to make higher order harmonics apparent, the collimation angle must be decreased, for example to 13 $\mu$rad ($1/(2\gamma )$), as shown by the red curve in Fig. 28. Channeling radiation contributes at energies above 50 MeV, reaching a broad peak at about 130 MeV (not shown in Fig. 28), which is about 7 times less intense than the first harmonic of CUR.

These results highlight the potential of crystalline undulators as compact and efficient gamma-ray sources. Their ability to generate high-brilliance radiation in an energy range not presently available at other LS makes them promising candidates for applications in particle physics, nuclear structure studies, and medical science.

### Coherent bremsstrahlung source based on collisions with short bunch ERL beam

The combination of high energies, high intensities, and very low emittances of electron and positron beams at the FCC-ee offers an extraordinary possibility of making a unique $\gamma$-source of very high brilliance. This involves using a novel technique [72], which in addition allows one to operate such a source concurrently with the nominal $e^+e^-$ collisions at the FCC-ee.

High energy bremsstrahlung (a.k.a. the radiative Bhabha scattering in $e^+e^-$ collisions) takes place at extremely large distances, both in lateral and longitudinal directions, with respect to the collision axis [73]. Its longitudinal coherence length is $l_{coh}=4\hbar c\gamma ^2/E_\gamma$, where $\gamma$ denotes the Lorentz factor of beam particles and $E_\gamma$ the bremsstrahlung photon energy. For high-energy beams $l_{coh}$ reaches macroscopic values and when it is larger than the bunch length $\sigma _z$ of the opposite beam, the bremsstrahlung process becomes coherent, that is, the radiating electrons^1^ interact with the entire bunch of the opposite beam and the bremsstrahlung rate becomes proportional to the total bunch charge squared [74]. Moreover, for flat beams, if the beams get separated vertically, the coherent bremsstrahlung is even amplified for separations as large as 20 vertical bunch widths and more! It should be noted that, to first approximation, the properties of the coherent bremsstrahlung do not depend on the energy of the ‘target bunches’ but only on their size and total charge.

**Fig. 29:**
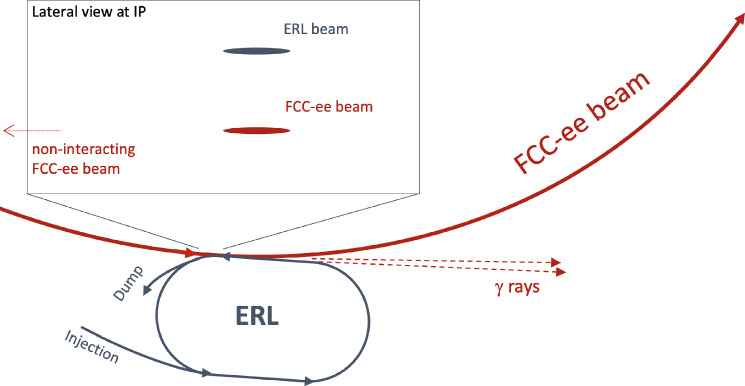
Schematic layout of the gamma-source at the FCC-ee

Based on these properties, a novel way is proposed for making a very bright $\gamma$-source at the FCC-ee, using one of the interaction regions (like the Point PL) where the high-energy beams do not collide and where one of them is then put in interaction with an opposite Low Energy Electron Beam (LEEB) which is largely separated vertically, as is illustrated in Fig. 29. To get a sufficiently low LEEB emittance its energy cannot not be too low and a 5 GeV energy is expected to be enough. The LEEB bunch length should be small to get a large maximal energy of radiated photons. Since the LEEB current should be relatively large, an ERL is proposed for the LEEB acceleration. In the following, a one-pass ERL is assumed, like in the bERLinPro project (but with more accelerating cavities), considering an RMS bunch length of 0.1 mm. The ERL bunch spacing matches the one of FCC-ee for a given beam energy. The maximal ERL beam power is about 30 MW, so that the maximal power consumption of such a facility should be around 1 MW even for the required highest LEEB currents.

For flat beams and head-on collisions, the coherent bremsstrahlung is governed by the parameter $\eta =r_e N_e/\sigma _{hor}$, where $r_e$ is the classical electron radius, $N_e$ is the LEEB bunch population and $\sigma _{hor}$ is the bunch horizontal size. The rate of coherent radiation scales with $\eta ^2$ but only if it is significantly smaller than 1. In the following, $\eta =0.5$ is assumed to obtain a maximal photon yield for such a coherent regime. The flux $\Phi$ of radiated photons per a 0.1%BW is independent of the photon energy for $E_\gamma$ significantly lower than the critical energy $E_{\textrm{crit}}=4\hbar c\gamma ^2/((1+\eta ^2)\sigma _z)$ [75], while for the photon energy $E_\gamma =E_{\textrm{crit}}$ it drops to 10% and very sharply beyond that energy; for $E_\gamma =3E_{\textrm{crit}}$ it is already down to $10^{-5}$ [74]. The parameter $\eta$ effectively provides the ratio of the angular deflection of a radiating electron in the field of the LEEB bunch to the average photon emission angle (equal to $1/\gamma$) – therefore, for $\eta =0.5$ such a deflection is negligible. The radiated power is more than an order of magnitude smaller than the beamstrahlung power expected in the high-energy $e^+e^-$ interaction regions [76], and the coherent bremsstrahlung has a softer spectrum. Therefore, the concurrent operation of such a gamma-source should not significantly change the high-energy beam parameters at the FCC-ee.

For $E_\gamma \ll E_{crit}$, the flux is given by the formula: $\Phi \simeq 5~10^{-4}\alpha \hspace{1.00006pt}\eta ^2N_bf\simeq 3.6~10^{-6}\eta ^2I_b/q_e$, where $\alpha$ denotes the fine structure constant, $N_b$ the bunch population, $I_b$ the FCC beam current, *f* the bunch crossing rate, and $q_e$ the elementary charge. The highest average brilliance $B=\Phi /(4\pi ^2\varepsilon _y\varepsilon _x)$, where $\varepsilon _y$ and $\varepsilon _x$ are the high-energy beam emittances, can be obtained assuming the collision optics for the high-energy beam, as in this case its angular divergences are significantly larger than the photon emission angle – this is assumed, along with a vertical beam separation of about 20 $\sigma _{y}$, for the results reported in Table 11, using the beam parameters from Table 1. The total flux of radiated photons above 0.1 MeV is extremely high, above $\mathrm 10^{16}~photons/s$ and close to $\mathrm 10^{15}~photons/s$ for the 45.6 and 120 GeV beams, respectively. The radiated photons are not coherent but are vertically polarised at about 60% [74].

**Table 11:** $\Phi , B$ and the peak brilliance for $E_\gamma \ll E_{crit}$ and two FCC-ee beam energies (and the LEEB bunch charges $Q_b$)

$E_{\textrm{beam}}$ [GeV]	$E_{\textrm{crit}}$	Flux $\Phi ~[10^{12}/$	Brilliance $[10^{20}/$	Peak $B~[10^{23}/$
($Q_b$ [nC])	[MeV]	$(\textrm{s}\hspace{1.99997pt}0.1\%\textrm{BW})]$	$(\mathrm{s\hspace{1.00006pt}mm}^2{\hspace{1.00006pt}\mathrm mrad}^2\mathrm{\hspace{1.00006pt}0.1\%\textrm{BW}})]$	$(\mathrm{s\hspace{1.00006pt}mm}^2{\hspace{1.00006pt}\mathrm mrad}^2\mathrm{\hspace{1.00006pt}0.1\%\textrm{BW}})]$
45.6 (0.15)	50	6.6	1.0	1.0
120 (0.2)	350	0.14	0.053	3.9

If the LEEB has 1 mm long bunches, the critical energies are 10 times smaller, whereas the ’asymptotic’ fluxes and brilliances stay the same as in Table 11. If instead of the collision optics assumed above some other optics is used, the fluxes stay the same, but the brilliance can be significantly lower.

In addition, one can envisage a stand-alone operation of the gamma-source with much larger bunch charges of LEEB when $\eta \gg 1$. In this case, the source would operate in the beamstrahlung regime, for which yet much higher photon fluxes and brilliances can be achieved, comparable to those in the high-energy $e^+e^-$ interaction regions, but with a vertical photon polarisation of 75%.

In summary, the proposed facility would produce, concurrently with the nominal operation of FCC-ee, the polarised gamma beams of very high brilliance and flat energy spectra for photon energies from 0.1 to 500 MeV.

### The FCC-ee and the gamma factory photon science

Multiple research opportunities, which can be addressed with high-intensity, MeV-range photon beams as research tools, have been identified and evaluated in the ongoing Gamma Factory (GF) studies. They cover many branches of physics: atomic physics [77–79], nuclear physics[80–82], particle physics[83–87], fundamental physics[88, 89], accelerator physics [90, 91], and applied physics [92].

Instead of using electron beams, the GF relies on highly relativistic atomic beams, stored in the existing LHC rings, as the photon-source driver beams. In the GF scheme [93, 94] photons are produced by exciting the atomic degrees of freedom of the stored particles by laser photons to produce polarised, or twisted photon beams.

**Fig. 30:**
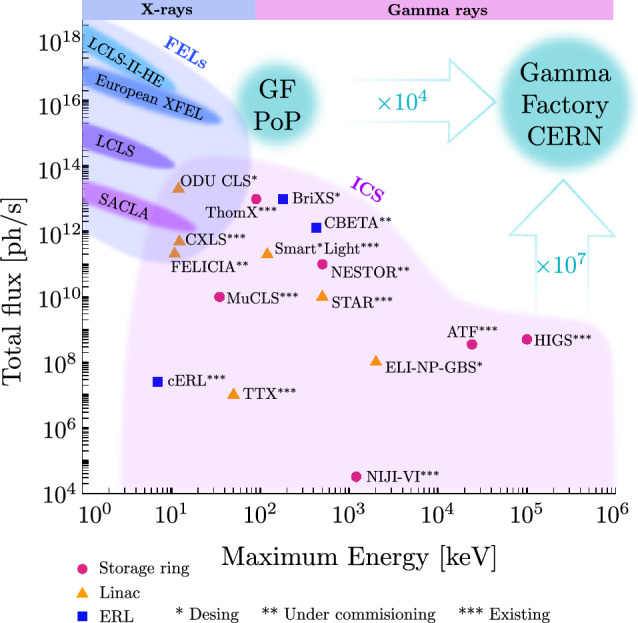
Fluxes and the energy range of the Gamma Factory photon beams

The intensity of the GF photon beams, as is illustrated in Fig. 30, can be higher than those of the present and the planned, future electron-beam-driven sources, by many orders of magnitude, albeit only in a restricted energy domain from 100 keV to 400 MeV. The FCC-ee photon beams can significantly extend this range towards lower and higher photon energies. In addition, the FCC-ee photon science applications could also cover certain fractions of the GF photon physics programme, which do not require extremely high photon fluxes.

## HEP applications

The applications presented in this chapter belong to the Physics Beyond Colliders (PBC) activity at CERN. These include uses of 20–46 GeV positrons, laser-Compton scattering, high-energy photons, and beam-crystal interactions, available from, or at, different branches of the FCC-ee accelerator complex.

### Dark matter searches

Here, we consider the unique high-intensity positron source from the FCC-ee booster, which will operate at 20 GeV and could potentially reach up to 46 GeV.

If a slow extraction of 20–46 GeV positrons from the FCC-ee booster can be realised, an experiment similar to NA64 could be conducted to explore new regions of the Light Dark Matter (LDM) parameter space. This would extend the sensitivity for the canonical dark photon model down to coupling values as low as $\alpha _D = 0.5$.

A key advantage of using positrons is the ability to exploit the resonant annihilation channel, which has been demonstrated by NA64 [95]. This process requires at least an order of magnitude ($\sim 10$ times) fewer statistics compared to the $A'$-strahlung production from electrons. This can be understood by comparing the cross sections for these processes given by:

14 $$\begin{aligned} A'\text {-Strahlung:} \quad&e^{\pm }Z \rightarrow e^{\pm }Z A' \quad \sigma _{\text {BRE}} \propto \varepsilon ^2 \alpha _{\text {EM}}^3 Z^2\; , \end{aligned}$$

15 $$\begin{aligned} \text {Resonant annihilation:} \quad&e^+ e^- \rightarrow A' \quad \sigma _{\text {RES}} \propto \varepsilon ^2 \alpha _{\text {EM}} Z\; . \end{aligned}$$

where $\varepsilon$ is the kinetic mixing strength of the standard model photon with the dark photon. The enhancement in sensitivity occurs in the mass range defined by:

16 $$\begin{aligned} \sqrt{2 m_e E_{\text {cut}}}< m_{A'} < \sqrt{2 m_e E_{\text {beam}}}. \end{aligned}$$

where $E_{\text {cut}}$ represents the missing-energy threshold while $E_{\text {beam}}$ the nominal beam energy For instance, with 20 GeV positrons, this corresponds to a mass range of:

17 $$\begin{aligned} 101 \text { MeV}< m_{A'}< 143 \text { MeV}, \quad 33 \text { MeV}< m_X < 47 \text { MeV}. \end{aligned}$$

**Fig. 31:**
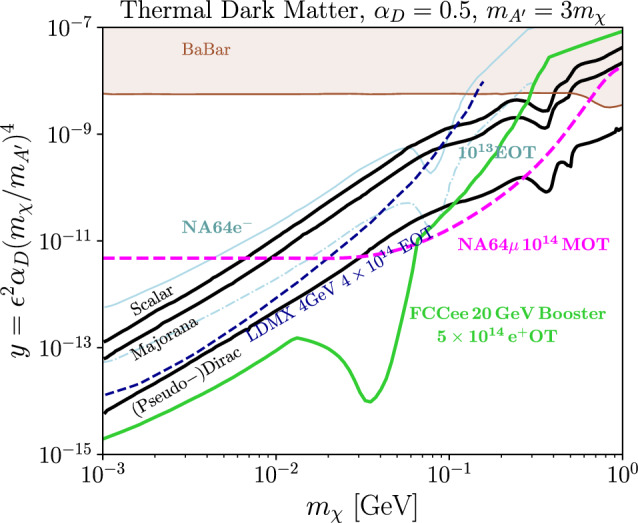
Projected sensitivity for a search of dark photons with the FCC-ee positron injector

The projected sensitivity for such an experiment is illustrated in Fig. 31. With an accumulated statistics of $4\times 10^{14}$ positrons on target, achievable within a 160-day data-taking period, significant improvements in the search for Light Dark Matter can be realised.

### Strong-field QED with electron-laser interactions

The use of Compton scattering off FCC-ee beams using conventional lasers for high-energy photon generation, beam intensity control or collimation, was discussed in Sect. 3.6. Section 4.4 describes the novel regime of full inverse Compton scattering with Unruh photons. Here and in the following subsection, we consider two other specific physics applications of laser-beam Compton scattering.

Strong-field quantum electrodynamics [96–100] describes the behaviour of charged particles and photons in strong electromagnetic fields, where nonlinear effects become significant. These effects emerge when field strengths approach the QED field strength scale, which is sometimes also called the critical field or the Schwinger limit $E_{\textrm{qed}} = m_e^2 c^3/(e \hbar ) \approx 1.32 \times 10^{18}~\mathrm {V/m}$, where $m_e$ is the electron mass, *c* the speed of light in vacuum, *e* the elementary charge, and $\hbar$ the reduced Planck constant. Processes like nonlinear Compton scattering and nonlinear Breit–Wheeler pair production, as well as the effect of vacuum birefringence, can be observed. With advancements in high-intensity laser technology, strong-field QED is now experimentally accessible. The FCC-ee with a beam energy up to about 183 GeV will provide a unique opportunity to probe this largely unexplored QED regime.

The strong-field QED phase space can be defined via two parameters. One is the classical nonlinearity parameter $\xi = e E \lambda _c / (\hbar \omega )$ also called the intensity parameter (although proportional to the square root of the laser intensity), $\lambda _c = \hbar / m_e c$ is the Compton wavelength and $\omega$ the angular frequency of the laser photons. The other one is the quantum nonlinearity parameter, also called the strong-field parameter, $\chi = e\hbar [-(F_{\mu \nu }p^{\nu })^2]^{1/2}/m^{3}c^{4}$, which in the plane-wave approximation takes the simple form $\chi = \gamma \, E / E_{\textrm{qed}}$, where $\gamma$ is the Lorentz boost of the electron and *E* the electric field strength of the laser pulse. In the context of electron-laser collisions it is convenient to also define the linear quantum parameter $\eta = \chi / \xi$ which is proportional to the momentum of the electron.

Strong-field QED is interesting because it is largely unexplored territory. Only in the last decade have lasers become powerful enough to access the strong-field regime of $\chi \gtrsim O(1)$. It is also interesting because it can be used to test non-perturbative predictions of quantum field theory. The intensity parameter, $\xi$, acts as the charge-field coupling. When $\xi \ll 1$, the interaction is perturbative and only the leading-order interaction with the electromagnetic background (e.g. the laser field) is necessary for an accurate prediction of experiments. However, when $\xi \not \ll 1$ (which can nowadays be achieved routinely in the lab), all orders of interaction between the charge and the electromagnetic background must be taken into account and the prediction for experiment is a test of *non-perturbativity at small coupling*. In addition, there are predictions for some processes that display a non-analytic dependency on the fine-structure constant $\alpha$, such as nonlinear Breit–Wheeler pair-creation in the tunneling regime [101, 102]. In this region, the process has a similar dependency on $\alpha$ as does Schwinger pair creation in a homogeneous electromagnetic field.

**Fig. 32:**
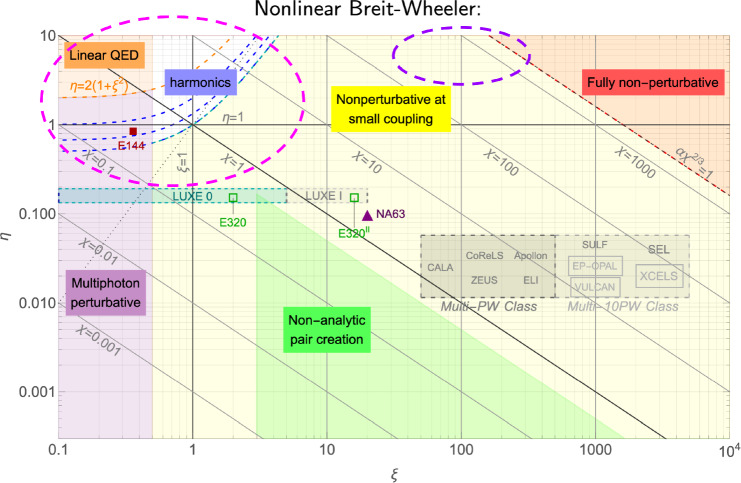
Phase space of the nonlinear Breit–Wheeler effect, adapted from [99], defined by the classical nonlinearity parameter $\xi$ and the linear quantum parameter $\eta$. The different regimes are indicated by the coloured areas. Two interesting regimes that can be tested at the FCC-ee are indicated by the pink and purple dashed lines

The two cases that seem most interesting for the timescale and parameters of a future Higgs factory are both indicated in Fig. 32: harmonic structure of the Breit–Wheeler process and the transition into the fully non-perturbative regime of QED. Following a conjecture made by Ritus and Narozhny [103], the scaling of higher (dressed) orders of loop diagrams in a constant crossed field is $\sim \alpha \chi ^{2/3}$ and not just $\sim \alpha$. Therefore, at $\chi \sim \alpha ^{-3/2} \sim O(10^{3})$, the perturbative expansion in the *radiated* field which forms the basis of the Furry picture of strong-field QED, should break down. Since there has been no method developed to calculate QED in this regime due to it being non-perturbative in both charge-field coupling and radiated field, experimental input, as might be achieved with the FCC-ee, is vital to make progress. To reach such high values of $\chi$ requires a high-power laser system of the 10 PW class and beyond, which are available already now (e.g. ELI-NP, Apollon, CoReLS) and should be much more common at the time needed at FCC-ee. A further requirement is to extract bunches from the booster, which can be achieved when the booster is idle from the collider program. The extracted electron bunch would then be collided with a laser pulse and the particles from the relevant processes measured (photons from nonlinear Compton, electrons/positrons from nonlinear Breit–Wheeler pair production, etc.).

#### New physics searches with photons

The high-energy photons produced via the Compton process in the electron-laser interaction points described above or in Sect. 4.2, or at other places in the collider, can be used in fixed-target new-physics searches. It has been shown that, at least in certain cases, using photons in a fixed-target search significantly reduces background events compared to electrons [104].

**Fig. 33:**
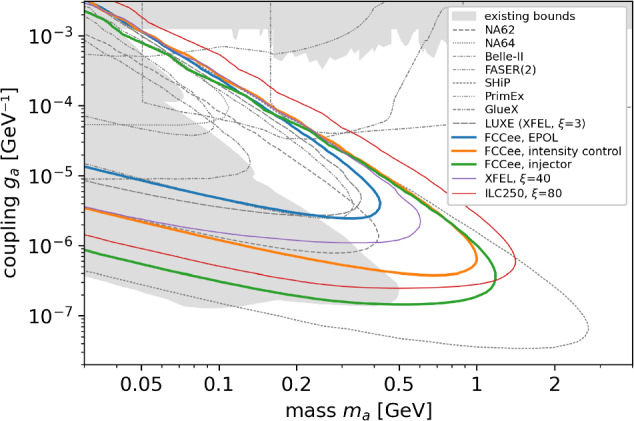
Phase space of the coupling constant of axion-like particles to photons $g_a$ and their mass $m_a$. It shows existing constraints (gray shaded), projections of existing and planned experiments (dashed/dotted), projections for future infrastructures (solid colours) for one year of runtime, i.e. $10^7$ s

To investigate the physics reach of the FCC-ee, we performed simulations for three scenarios: using photons produced by the EPOL polarisation measurement [105] and by the intensity control [106], as well as using a dedicated PBC facility at the injector complex, as summarised in Table 3. For the latter, the laser of the Compton source was optimised to produce the largest number of photons with energy above 1 GeV. The simulation assumed the production of axion-like particles from the primary photons via the Primakoff process [107–109] in a 2 m-long tungsten target and the detection of two photons from the decay of the axion-like particle in a 10 m long decay volume. We also assumed a detector radius of 5 m and a background-free environment. The results of the simulations are shown in Fig. 33, and details of the study can be found in Ref. [110].

Comparing the projected phase-space coverage of axion-like particles coupling to photons of various possible Compton sources at the FCC-ee with the newest SHiP projection [111] shows that FCC-ee can not explore new phase-space in such a scenario. However, the approach remains highly complementary since this method leverages photon interactions via the Primakoff production mechanism directly. This provides a unique and independent method to investigate the ALP-photon interaction. Since the parameter space for the possible existence of ALPs is still unknown, employing a variety of search approaches is crucial to enhance the chances of discovery.

### Strong field QED and positron/electron spin dynamics in crystals

A high-energy, low-emittance, high-intensity positron beam available at FCC-ee (up to 183 GeV) would enable, for the first time, the observation of spin dynamics effects in strong-field QED in oriented crystals.

When the velocity of a particle is nearly parallel to a crystal axis or plane, successive collisions with atoms in the same plane or row are correlated. This allows the replacement of the screened Coulomb potential of each individual atom with an average continuous potential of the entire plane or string, which corresponds to a strong electric field $E \approx 10^{10} - 10^{12}$ V/cm [49].

In the high-energy regime of interest, the particle’s wavelength of transverse motion is much smaller than the spacing between crystallographic planes. As a result, particle motion is quasi-classical. If the incidence angle $\psi$ of the charged particle with respect to the crystal planes or axes is smaller than the critical angle defined by Lindhard, $\theta _c = \sqrt{2U_0/\epsilon }$, where $\epsilon$ is the particle energy and $U_0$ the amplitude of the average potential energy, this particle can be trapped in an oscillatory motion within the corresponding potential well–this phenomenon is known as channeling.

In the particle’s rest frame, the average electric field it experiences is amplified by a factor of $\gamma$ as a result of Lorentz contraction. At sufficiently high energies, this transformed field can approach the Schwinger critical field of quantum electrodynamics (QED), given by $E_0 = m^2 c^3/(e\hbar ) = 1.32 \times 10^{16}$ V/cm [40, 112, 113]. Close and above this threshold, electrodynamics becomes nonlinear, as observed in extreme astrophysical environments such as pulsar atmospheres. This strong-field limit can be reached “easily” in crystals if the energy of an $e^+/ e^-$ beam attains tens or hundreds of GeV, for which the parameter $\chi = \gamma E / E_0$ approaches unity. In this regime, quantum synchrotron radiation dominates, leading to intense emission of hard photons by $e^+/ e^-$, along with enhanced pair production from high-energy photons. These effects have been studied for decades at the CERN SPS extracted beamlines, where secondary beams of $e^+/ e^-$ up to 200 GeV are available, using both straight and bent crystals [112, 114, 115]. This includes investigations of quantum and classical radiation reaction effects and trident production by CERN SPS NA63 [116, 117].

The availability of high-energy, high-quality positron / electron beams (intense primary beams with low divergence) of up to 183 GeV energy at the future FCC-ee accelerator complex would allow for much higher statistics and improved experimental performance, which is crucial for strong-field QED studies.

**Fig. 34:**
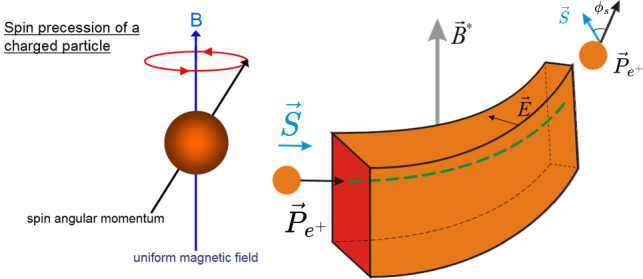
Spin precession of a channeled positron in a bent crystal. Adapted from Refs. [118, 119]

More intriguingly, the higher positron beam quality and statistics would enable us to investigate **unexplored strong field effects on positron / electron spin dynamics**, exploiting channeling in bent crystals. Indeed, in a bent crystal, a particle follows a curved trajectory under the influence of the electric field *E* induced by the crystallographic plane. In the particle’s instantaneous rest frame, relativistic transformations generate a magnetic field $B^*$ that interacts with the particle’s magnetic moment, causing spin rotation (Fig. 34) [118].

At the highly ultra-relativistic energies the spin rotation angle is mainly determined by the anomalous magnetic moment $\mu '=(g-2)/2$. The existence of spin rotation for high-energy particles moving in the channeling regime of bent crystals was first demonstrated in [120] and is currently under investigation at CERN by the TWOCRYST/ALADDIN collaborations based on the LHC [121], which aim at measuring the magnetic dipole moment (MDM) and search for electric dipole moment (EDM) effects in short-lived charmed baryons, with potential sensitvity to new physics.

**Fig. 35:**
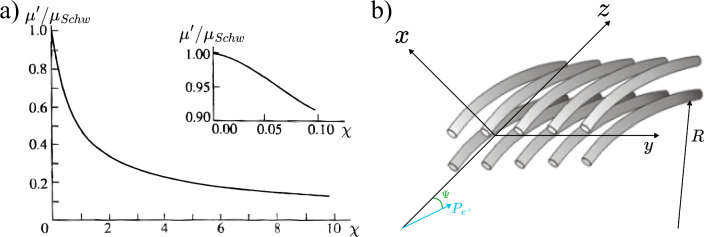
**a** Anomalous magnetic moment $\mu$’ of ultrarelativistic e$^{\pm }$ that move in a uniform electric field E as a function of $\chi = \gamma E/E_0$. The magnetic moment is measured in units of Schwinger value $\mu _{\textrm{Schw}} =(\alpha /2\pi )\mu _B$, where $\alpha = 1/137$ and $\mu _B$ is the Bohr magneton. Adapted from [124]. **b** The geometry with bent crystallographic axes and planes defined by the bending radius R, proposed for the experiment on positron’s anomalous magnetic moment modification; $\psi$ is the angle with crystal axes when the positron moves along crystal planes. Adapted from [125]

At FCC-ee, spin rotation by channeling in bent crystals would provide a **unique opportunity to study strong-field QED spin dynamics effects**, such as the considerable reduction of the anomalous magnetic moment $\mu '$ in strong field at a high particle energy, when the parameter $\chi$ is comparable and higher than unity [122, 123]. This effect arises because virtual particles no longer propagate freely but instead experience modified propagation in strong electric/magnetic fields, leading to the suppression of loop corrections. Therefore, measuring the spin precession angle for positrons in a bent crystal at FCC-ee energies would provide a **direct test of strong-field QED**. Figure 35 illustrates the predicted decrease of the anomalous magnetic moment of an electron/positron as a function of the parameter $\chi$.

*How is it possible to measure the* $\mu$’ *modification in the strong crystal field?* When a positron undergoes planar channeling in a bent crystal, it moves through regions of preferred directions of the quasi-uniform planar electric field, creating a preferred direction that drives spin precession [118]. If, at the same time, it is oriented close to a crystal axis in the so-called “String-Of-Strings” (SOS) configuration (Fig. 35 b)), the electric field is even stronger than in the planar case, leading to an effective electric field $E^*$ (equivalent to a magnetic field $B^*$) that exceeds the Schwinger limit. For instance, in a cooled germanium crystal (100 K), the effective field $E^*$ surpasses the Schwinger limit at approximately 50 GeV ($\chi = 1$).

*Why positrons and not electrons, since the value of* $\mu$’ *is the same?* That is because positively channeled particles move far from planes in a region of lower nuclear density, therefore are less subject to incoherent scattering with nuclei that causes dechanneling. In other words, positrons offer a more stable planar channeling motion. The angular value of spin rotation, $\phi _{s}$ is related to $\mu '$ through the following formula,

$$\begin{aligned} \phi _{s} = \phi _{s} (\phi ) = \left( \dfrac{\mu '}{\mu _{B}} \dfrac{\gamma ^{2}-1}{\gamma }+ \dfrac{\gamma -1}{\gamma }\right) \phi \approx \gamma (\mu '/ \mu _{B})\phi \end{aligned}$$

where $\phi$ is the bending angle and $\mu _B$ the Bohr magneton.

**Fig. 36:**
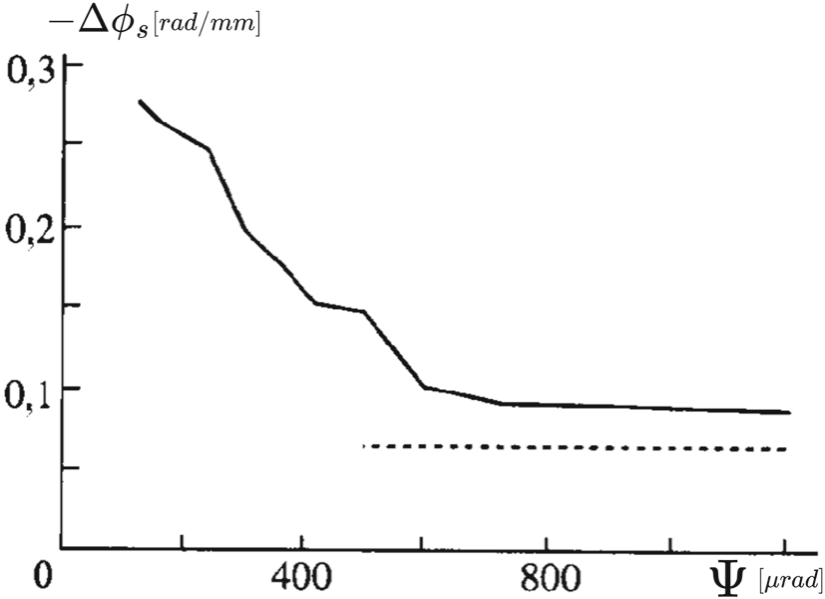
Reduction of e$^+$ spin rotation angle associated with a decrease in $\mu$’ as a function of the angle of positron incidence on the <110> axes of Ge. Positrons move in channeling in the field of (100) planes; $T= 77$ K, $E_{e^+} = 90$ GeV, $R_{\textrm{bending}} = 20$ cm and crystal length $L = 1$ mm. Adapted from [124]

At $\mu ' = \mu _{Schw}= \alpha /(2\pi )\, \mu _B$, i.e. in the absence of magnetic moment modification, the spin precession angle is approximately $\phi _s \approx 1~\mathrm {rad/mm}$ for 90 GeV channeled positrons in a crystal with a bending radius of 20 cm. Figure 36 shows the reduction of $\phi _s$ induced by the modification of the magnetic moment in a cooled germanium crystal (at 77 K), plotted as a function of the incident angle $\psi$ with respect to the crystal axes [124]. During their motion, positrons emit intense radiation due to the interaction with the strong electromagnetic field in the crystal. As a result, both the quantum parameter $\chi$ and the effective magnetic moment $\mu '$ vary along the trajectory. Therefore, high-statistics simulations or measurements are essential to isolate the subset of channeled positrons that experience minimal energy loss throughout their passage in the crystal. This ensures that their $\chi$ parameter remains nearly constant over the full spin precession length.

**Fig. 37:**
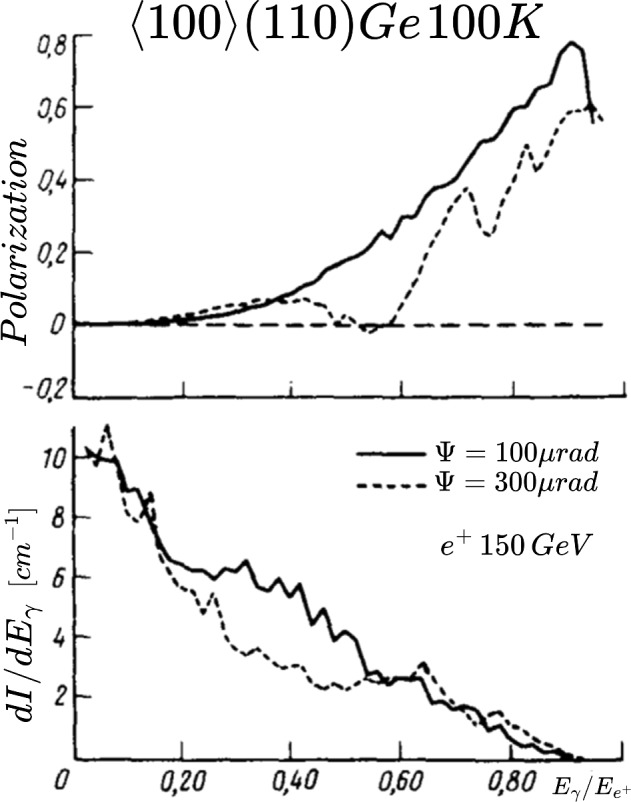
Transverse polarisation (up) acquired by 150 GeV positrons in a cooled (100 K) Ge crystal in planar channeling in the fields of (110) planes, at different orientation, $\psi$, with the <100> axes depending on the radiative losses $I\equiv \Delta E/ds$ (bottom). The larger are the radiative losses, the larger is the acquired degree of polarisation. Adapted from [125]

Bent crystals do not only allow measuring drastic magnetic moment decreases of the positron (electron) [122, 124], but also observing radiative self-polarisation [125–127]. In synchrotrons, electrons become polarised due to **Sokolov-Ternov self-polarisation**. This phenomenon occurs because electrons emit synchrotron radiation while circulating in a magnetic field, and this radiation is slightly spin-dependent. Over time, this causes a net polarisation of the electron beam. The polarisation build-up time is inversely proportional to the field strength cube and takes macroscopic time in synchrotrons, while in the strong crystal field positrons become highly polarised over a length of less than a millimeter after emitting a few or even one hard photon, demonstrating thus a **quantum spin-flip** effect. Figure 37 illustrates the transverse polarisation, acquired by 150 GeV positrons in cooled Ge. One can notice that the effect is considerable and can be used both for production and measurement of positron polarisation.

Finally, bent crystals also allow observing circularly polarised gamma-radiation by positrons, polarised electron-positron pair production by gamma-quanta and positron spin rotation in the circularly polarised crystal field harmonics [128–132].

### Full Inverse Compton scattering and Unruh photons

Compton back scattering off FCC-ee beams using conventional laser is discussed in Sect. 3.6. One interesting and still unexplored regime in the photon-electron Compton interaction is the Full Inverse Compton Scattering (FICS) [133, 134], occurring between electron beams of any energy and photons of energy around 255.5 keV, that is $0.5m_ec^2$.

**Fig. 38:**
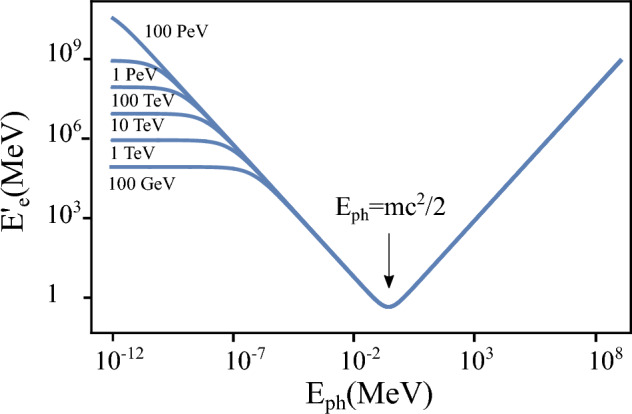
Full inverse Compton scattering: energy of the scattered electron as a function of the initial photon energy, for different initial electron energies

In this regime, the entire kinetic energy of an electron can be transmitted to a photon, since the electron is taken down to rest in the laboratory reference frame after collision with the photon. Figure 38 shows the total electron energy $E'_e$ (in MeV) after the scattering versus the energy of the incoming photon $E_{ph}$ (in MeV) for several values of the electron initial energy $E_e=\gamma m_ec^2$, starting from 100 GeV going up to 100 PeV. Whatever their initial energy, the electrons are stopped by photons with energy close to 255 keV. The minimum, centered at the value $E'_e=m_ec^2$, is very shallow, indicating that the requirement on the mono-chromaticity of the incident photon beam is not prohibitive. In this regime, the Compton recoil factor *X*^2^ is about 2$\gamma$, and the energy of the emitted photons approaches $\gamma m_ec^2$. In particular, photons fully back-scattered after colliding with the electrons carry an energy equal to $T_e+0.5m_ec^2$, where $T_e$ is the initial kinetic energy of the electron, that is $T_e=(\gamma -1) m_ec^2$. The basic difference with standard Inverse Compton scattering discussed in Section 3.7 (ICS), is the value of the recoil parameter *X*, that in ICS can be larger than 1 but which is always much smaller than $\gamma$, while in FICS it is equal to $2\gamma$. As a result, in ICS the transfer of energy from the electron to the photon can reach the full transfer only asympotically when $\gamma$ tends to infinity, while in FICS it is achieved exactly for any value of $\gamma$ if $E_{ph}=0.5m_ec^2$. In Fig. 38, the curve representing the envelope of all lines, each corresponding to a specific value of the initial electron energy $E_e$, is given by the expression $E'_e=E_{ph}+({m_e}^2c^4)/(4E_{ph})$, which is valid whenever both $\gamma$ and *X* are much larger than 1. Individual lines corresponding to specific values of $E_e$ are departing from the common envelope when the value of the recoil parameter *X* is getting close to 1 or smaller than 1, that is when the interaction occurs in the Thomson (classical) limit with negligible recoil, represented by the plateaus shown on the left side of the plots.

The rate of the process is weighted by the Klein-Nishina cross section and presents a concentration of events around the back-scattered direction.

**Fig. 39:**
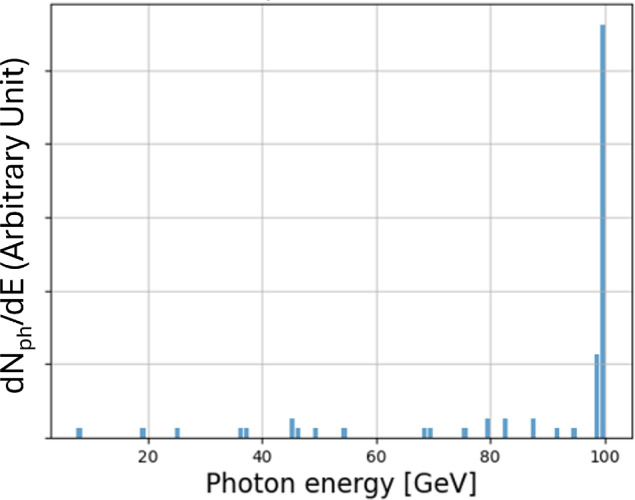
Full inverse Compton scattering: photon energy spectrum for initial electron energy of 100 GeV and initial photon energy of 255.5 keV

**Fig. 40:**
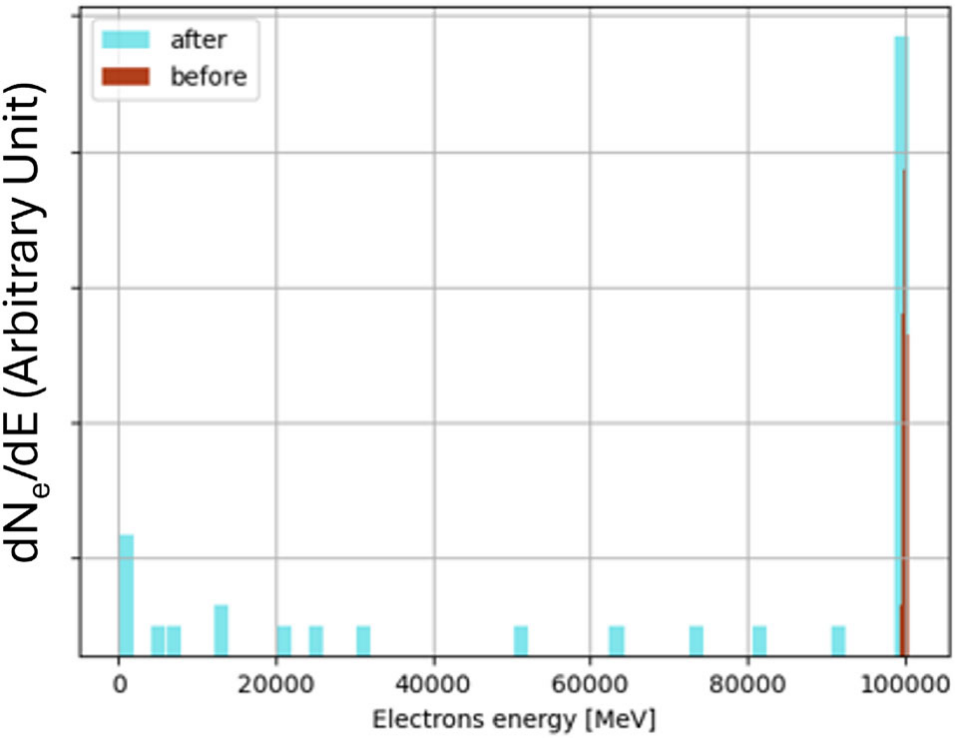
Full inverse Compton scattering: electron energy spectrum after collision, for initial electron energy of 100 GeV and initial photon energy of 255.5 keV

Figures 39 and 40 show the energy spectrum of the scattered photons and the electrons in a situation similar to the FCC-ee’s, simulated by CAIN [135]. Electrons of 100 GeV are colliding with 255.5 keV photons.

Figures 39 and 40 present the spectral energy distributions of an arbitrary number of events generated under the FICS condition, as simulated by the code CAIN [135]. The simulations consider collisions of 100 GeV electrons with 255.5 keV photons to illustrate the characteristics of the scattered photon beam which the FICS interactions at the FCC-ee could produce, assuming a primary X-ray beam with a photon energy of $m_e c^2/2$. The bunch of emitted photons at nearly the same energy as the primary electrons can be observed on the right of Fig. 39, while the corresponding few electrons remaining with only their mass energy (i.e. zero kinetic energy) appear on the left side of Fig. 40.

The total number of events actually generated by a single FCC-ee bunch collision with a 255.5 keV X-ray pulse will depend on the actual layout of the interaction and on the intensities of both electron and photon beams. The modelling of possible experiments at the FCC aimed at investigating the physics of such a process is under study. Furthermore, FICS corresponds to the fastest deceleration known to date resulting in Unruh radiation at a temperature of 100 MK. Figure 15 of Ref. [134] features the possible acceleration values attained in FICS condition with electron energies up to tens of GeV. FICS can offer the opportunity to test maximal acceleration theories and their predicted modifications to relativity due to the existence of a maximal acceleration. In the domain of astrophysics, FICS could be a mechanism to explain the propagation of high energy photons in the Universe.

## Positron applications

### Overview of slow positron beam facilities

Positrons, the antimatter counterparts of electrons, are produced and utilised through various sources, each serving distinct scientific and industrial purposes. These sources can be broadly categorised into radionuclide-based, reactor-based, and accelerator-based systems, distributed globally and offering a wide range of beam intensities and energies.

Radionuclide-based sources, such as those used in Positron Emission Tomography (PET), are instrumental in medical imaging and clinical diagnostics. These sources rely on low-activity isotopes that emit few positrons during radioactive decay, providing a weak source suitable for non-invasive imaging techniques. PET remains a cornerstone in medical research, aiding in cancer detection and neurological studies.

Reactor-based positron sources are prominent in academic and industrial research due to their higher beam intensities. The Neutron Induced Positron Source Munich (NEPOMUC), located at the FRM-II research reactor in Garching, Germany, exemplifies such facilities. NEPOMUC generates highest-intensity beams by utilising moderated positrons derived from neutron interactions. Similar setups exist worldwide, including the Institut Laue-Langevin (ILL) in France, Kyoto University Research Reactor (KUR) in Japan, POSH at Delft University of Technology in the Netherlands, and PULSTAR at North Carolina State University in the USA. These reactors provide invaluable tools for studying material defects, surface phenomena, and other physical properties, but are bound to the operation of the nuclear reactor and the availability of fissionable source material.

Accelerator-based positron sources represent the forefront of high-energy physics, with applications ranging from fundamental particle studies to advanced material science. Many of these sources operate at repetition rates of 10–100 kHz, producing moderated positron beams within energy ranges of 0.05–20 keV. Key materials used for positron moderation, i.e. to slow them down from several MeV to keV or less, include tungsten, due to its robustness and efficiency, and newer materials such as 4H/6H silicon carbide (SiC), which offer enhanced performance. Such sources are crucial for experimental setups requiring precise beam control, including low-energy positron implantation and positronium-based research.

The global distribution of these facilities enables collaborative research and technological advancements. Efforts towards achieving Bose-Einstein condensates (BECs) of positronium, a bound state of an electron and a positron, highlight the interplay between positron source technologies and cutting-edge physics. Similarly, positron sources contribute to testing fundamental symmetries, precision quantum electrodynamics (QED) studies, and searches for physics beyond the Standard Model (BSM), including investigations into fifth forces and axion-like particles.

In addition to fundamental research, positron beams are invaluable in material science. Non-destructive probing techniques enabled by positron annihilation spectroscopy provide insights into nanostructures and defect distributions, with applications spanning semiconductors, polymers, and advanced alloys. Emerging positron sources promise next-generation capabilities, offering higher intensity, lower energy, and greater beam density, which can significantly enhance data quality and measurement efficiency.

Overall, the continuous development and optimisation of positron sources are vital for unlocking new scientific possibilities. By improving beam intensity, moderation techniques, and source reliability, these systems will further expand their applications, fostering global collaboration in both academia and industry.

### New experiments with dense positronium clouds

The access to a high number of positrons, as would be provided by the FCC-ee injector complex, can be used to advance positronium physics in different areas. Of particular interest are the applications where high positronium density is required and in case the investigated process has a low cross section. To reach high atomic density, a high number of positrons can be combined with laser cooling techniques [136, 137]. Here we review a few experiments proposed in the literature which would greatly benefit from access to high density positronium clouds.

**Fig. 41:**
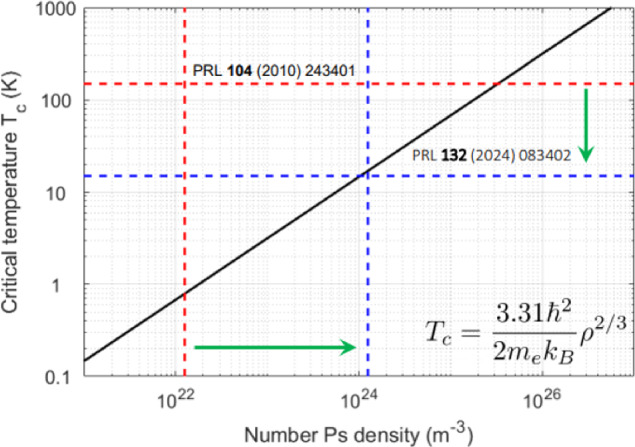
Critical temperature versus Ps density: Present and future efforts towards the first Bose-Einstein condensation of Ps (image courtesy of Ruggero Caravita)

As previously mentioned, reaching Bose-Einstein condensation (BEC) of positronium is a long-standing goal of antimatter research which can only be achieved with high enough atomic density and low enough temperature, as is illustrated in Fig. 41. One interesting prospect of a positronium BEC is the possibility to form a Gamma-ray amplification via stimulated annihilation radiation (GRASAR) [138] by coherence transfer between the BEC and the GRASAR emission. Because of the sub-nanosecond annihilation lifetime of para-positronium, a Ps BEC will most likely be formed in the ortho-positronium state, whose lifetime is three orders of magnitude longer. To initiate the two-photon annihilation, a radiation at 0.2 THz can be used to drive the triplet to singlet state transition. Also, the BEC should be formed in an elongated shape so that a macroscopic population of spontaneously emitted annihilation photons at 511 keV get amplified into end-fire modes. Similar tracks are being explored in $\mathrm {^{135m}Cs}$ BEC [139].

A high density positronium source is also necessary to demonstrate the muon anti-muon pair creation from positronium in a linearly polarised laser field [140]. In this scheme, positronium atoms are submitted to the strong oscillating electric field of a femtosecond laser pulse. The electron and positron are accelerated in a $\simeq 10^{22}\mathrm {W cm^{-2}}$ laser field. When the electric field changes sign, the two particles are headed back towards each other with a center of mass energy larger than the $\simeq$200 MeV threshold required to form a muon anti-muon pair. Laser systems able to produce intensity above $10^{23}\mathrm {W\,cm^{-2}}$ are now available [141, 142]. They include very strong focusing of light on areas with a typical size of the order of $\mu$m. In these conditions, laser-driven re-collision processes in Ps can only be observed if a sufficient number of atoms are found in the interaction region defined by the laser focus, that is in Ps clouds of high enough density.

In addition, high density clouds of Ps could help produce and study positron and positronium binding ordinary matter species, for example via charge exchange between anions and Ps [143]. These exotic matter–antimatter systems have long been predicted to exist but even their existence is very difficult to prove. Pulsed production of a large number of these atoms with high density Ps clouds would open up new possibilities in terms of spectroscopy of these systems, for example, using techniques inspired from ultrafast physics phenomena [144]. In this case, the low event probability as well as the small interaction area between the laser and the species to be probed (defined by the laser focus waist in many experiments) requires a high number of positronium and preferably a high density of Ps.

### Material sciences with high-brightness, low energy positron beam

**Fig. 42:**
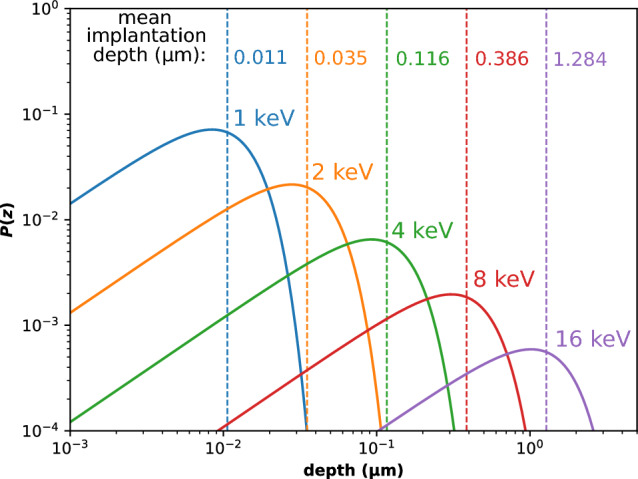
Examples of Makhovian positron implantation profiles *P*(*z*) calculated for diamond: As the implantation energy increases, positrons penetrate more deeply into the material, enabling depth-resolved measurements

**Fig. 43:**
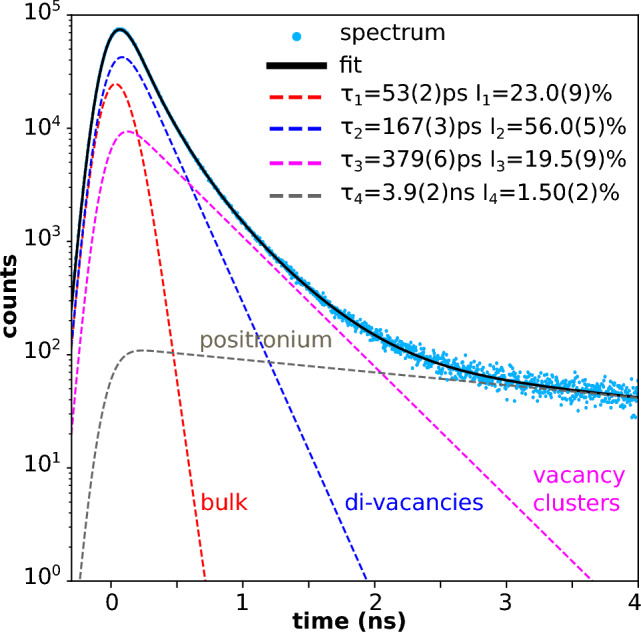
Example of a positron lifetime spectrum in diamond: The measured data can be decomposed into distinct lifetime components ($\tau _i$), each corresponding to a different annihilation process within the material. These lifetimes provide valuable insights into open-volume defects in the crystal. The associated intensities ($I_i$) reflect the relative occurrence of these annihilation events. This spectrum and the corresponding research can be found in Ref. [163]

Positron annihilation spectroscopy (PAS) techniques are well-established, non-destructive methods in modern material science. The use of an intense, mono-energetic positron beam of variable energy significantly enhances both the versatility and quality of the acquired spectra. By adjusting the kinetic energy of the positron beam, positrons can be implanted at different depths below the surface, enabling depth-resolved measurements. The implantation depth is described by Makhovian profiles [145], which depend, among other factors, on the material density. Figure 42 illustrates examples of Makhov profiles in diamond for mono-energetic positron beams at various energies. In principle, energy variation allows most positron spectroscopy techniques to acquire spectra as a function of depth. This capability is particularly advantageous for analysing thin films and layered structures. The following selection provides an overview of the most important techniques in this field:

- *Positron Annihilation Lifetime Spectroscopy (PALS)*: PALS is a powerful technique for identifying vacancy-like defects, such as single vacancies or vacancy clusters, in crystalline materials. Additionally, it enables the characterisation of nanoscale pores ranging from 0.1 to 3 nm in size in non-conductive materials like polymers. The applications of PALS span various fields, including defect characterisation in semiconductors [146, 147], perovskites [148, 149], and quantum materials [150]. Furthermore, it is widely used in the analysis of radiation-damaged materials for fusion research [151, 152], as well as in the study of free volume elements in metal-organic frameworks [153] and thin-film membranes for water filtration [154, 155]. An example of a positron lifetime spectrum and its decomposition into several lifetime components is shown in Fig. 43.
- *Doppler Broadening Spectroscopy (DBS)*: DBS analyses the momentum distribution of electrons in a material by measuring the Doppler broadening of the 511 keV gamma line. This technique provides insights into defect densities and the local chemical environment. Applications include, e.g. the study of precipitates in metals [156] or the influence of defects in superconductor materials [157].
- *Angular Correlation of the Annihilation Radiation (ACAR)*: ACAR enables the determination of Fermi surfaces by measuring the angular deviation of annihilation gamma rays. This technique provides valuable insights into the electronic properties of complex materials, such as electron–electron interactions in ferromagnetic systems [158] or momentum densities in metals [159].
- *Positron-induced Auger Electron Spectroscopy (PAES)*: PAES is a highly element-specific technique for analysing material surfaces. It enables the investigation of only a few monolayers while offering nearly background-free spectral acquisition. This makes it particularly useful for applications such as the study of catalytic materials [160, 161].
- *Total-reflection high-energy positron diffraction (TRHEPD)*: TRHEPD is a scattering technique in which positrons interact with material surfaces under total reflection conditions. Due to their shallow penetration depth, limited to a few atomic layers, this method provides precise structural information about monolayers such as graphene or surface reconstructions of semiconductors [162].

#### NEPOMUC: A Benchmark for Intense Positron Beams

The NEutron induced POsitron source MUniCh (NEPOMUC) has set a global standard for positron beam intensity, achieving beam currents exceeding $10^9$ positrons per second [164, 165]. Its capabilities underscore the profound scientific advantages of intense positron sources: reduced acquisition times, enhanced sensitivity to low-abundance defects, and the ability to perform statistically robust studies on subtle phenomena. Currently, five experiments at NEPOMUC are dedicated to material science investigations:

- *The Coincidence Doppler-Broadening Spectrometer CDBS*, which enables Doppler-broadening spectroscopy with a lateral resolution of several tens of micrometers [166].
- *The pulsed low-energy positron system PLEPS*, designed for depth-resolved lifetime spectroscopy [167].
- *The Scanning Positron Microscope SPM*, which utilises a pulsed positron microbeam to perform three-dimensional defect microscopy with micrometer resolution [168–170].
- *The Surface Spectrometer SuSpect*, used for positron-induced Auger electron spectroscopy [171].
- *The positron scattering experiment TRHEPT*, dedicated to surface investigations [172].

Additionally, NEPOMUC hosts an experiment focused on fundamental research into electron-positron pair plasmas [173, 174]. However, current limitations, particularly regarding beam brightness and energy resolution, and simply the reduced up-time of the reactor source, remain critical for material science and fundamental research.

#### The Promise of FCC-ee Positron Beams

The FCC-ee, envisioned as a next-generation particle collider, has the potential to revolutionise positron beam science. By exploiting the high-luminosity environment and advanced beam dynamics of the FCC-ee, a positron source with superior brightness, monochromaticity, and energy tunability could be developed. Such a source would address current limitations, including:

- *Energy Resolution*: Improved energy spread control would enhance beam pulsing crucial for experiments like the SPM or PLEPS. Furthermore, it will improve surface sensitive techniques like PAES or TRHEPT.
- *Beam Brightness*: Increasing the beam brightness would enhance the focusing capabilities of positron beams, thereby improving the lateral resolution of experiments such as the SPM or the CDB Spectrometer.
- *Reduced Measurement Times*: Higher intensities could shorten acquisition times, making positron techniques more accessible for routine industrial applications.

Despite these promising prospects, several challenges must be addressed. Designing a positron source at the FCC-ee with sufficient intensity and focusability will require innovative beamline engineering and advanced moderation techniques. Additionally, ensuring compatibility with existing positron annihilation spectroscopy systems will be critical for seamless integration into the scientific community.

A high-brightness, low-energy positron beam at FCC-ee could overcome many limitations of current sources, unlocking new capabilities for material sciences. Building on the legacy of intense positron beams and leveraging state-of-the-art advancements, this next-generation source has the potential to drive breakthroughs in defect engineering, quantum material studies, and nanoscale analysis. By addressing existing technological hurdles, the FCC-ee positron beam could become a cornerstone for future innovations in material science.

## True Muonium production

True Muonium (TM) is the yet un-observed, purely leptonic bound state of a muon and an anti-muon, $(\mu ^{-}\mu ^{+})$. As an extremely compact and heavy bound-state QED system, with a Bohr radius of $a_0^{TM}=2/\alpha m_\mu \simeq 512$ fm, it is not only a very interesting object to test bound-state QED but also an ideal candidate to search for New Physics in the muonic sector (see, e.g. Ref. [175, 176]).

It was proposed that TM could be produced at the SPS H4 beam line with 43.7 GeV positrons, which corresponds to the threshold for di-muon production in a fixed target configuration [177]. The cross section is given by [178]:

18 $$\begin{aligned} \begin{aligned} \sigma ^{e^{+}e^{-}\rightarrow (\mu ^{+}\mu ^{-})}_{(n)}(E_{+})&= \frac{3\pi \alpha }{2}\frac{\delta E_{n}}{\Delta E_{+}}\times \sigma ^{e^{+}e^{-}\rightarrow \mu ^{+}\mu ^{-}}(E_{+})\\&\simeq \frac{\delta E_{n}}{\Delta E_{+}} \times 9.11 \cdot 10^{-32}\,\textrm{cm}^{2} \end{aligned} \end{aligned}$$

where $\delta E_{n} = \alpha ^2 m_{\mu }/(4n^2)$ is the effective energy window to produce the bound states and $\Delta E$ is the beam energy resolution.

The hadronic contamination at H4 was studied in the context of NA64 running in positron mode [179]. The maximal beam intensity at H4 is $\sim 1\times 10^7$ e$^+$/spill (this result in about $3\times 10^{10}$ e+/day) while the energy spread is estimated to be around 1%. Recently, a study for the target optimisation (40 low-Z Lithium foils) was conducted showing that in principle a discovery of TM at the 5 sigma level could be achieved in 3 months of data taking [180].

The FCC-ee injector and booster complex would increase, by many orders of magnitude, the number of positrons available. Between regular FCC-ee filling cycles, in most of the FCC-ee modes, the booster is idle as indicated in Fig. 5. During this time, it could be run at 43.7 GeV for the TM production. At this energy, roughly 1 s is required for a booster ring cycle. The rest of the time can be used to accumulate positrons, at a rate of about 10$^{13}$ e$^+$/s. In WW mode, the booster is available for 1.78 s per cycle of 5.5 s, translating into an average rate of $1.4 \times 10^{12}$ e$^+$/s. In the ZH and $\textrm{t}\bar{\textrm{t}}$ modes, the booster is available for 5.6 s and 5.4 s per cycle of 10 s, resulting in average rates of $4.6 \times 10^{12}$ e$^+$/s and $4.4 \times 10^{12}$ e$^+$/s, respectively, for true muonium production.

The beam will have an energy spread of only 0.1% as indicated in Table 3, which is much better than at H4. Assuming $\Delta E \sim 44$ MeV, one can expect to produce around 1 TM atom per $4.4 \times 10^{13}$ positrons. Ideally, as for the search of DM matter described in Sect. 4.1, the beam should be slowly extracted to minimise the combinatorial background.

A preliminary setup to show the feasibility of the technique consists of a target 1 dissociation length thick, followed by a 0.5 m vacuum tube ending with a set of 4 tracking detectors. The decay products of the TM decay are then separated by a 0.2 T/m magnetic field to reconstruct the invariant mass of the particle to efficiently suppress the background. Since TM is produced at threshold, the pairs produced by Bhabha scattering can mimic the signature. However, those can be rejected using a precise reconstruction of the vertex position, expected to be $\sim$10 cm downstream of the target.

**Fig. 44:**
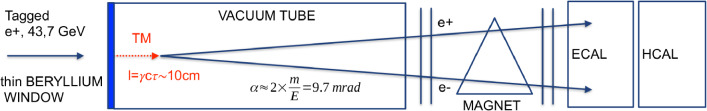
Simplified layout of the experimental set-up at the H4 beamline. A similar set-up could be considered for 43.7 GeV positrons from the FCC-ee booster

The setup is further equipped with an Electromagnetic calorimeter (ECAL) and 4 Hadronic calorimeter modules (HCAL) placed after the spectrometer for background rejection and further signal characterisation. A simplified sketch of the setup is depicted in Fig. 44. A detailed MC simulation predicts that a background rejection at a level of 10$^{12}$ positrons on target is possible, which is at the same level as the expected rate of TM.

With the above estimated rates of $10^{3-4}$ TM/day, the properties of the bound state could be studied. The decay time can be examined from the reconstruction of the decay vertex. Furthermore, there might be the possibility to measure the hyperfine structure of the atomic energy levels and the Lamb shift. This would additionally require a high-power laser system. More detailed studies are required to assess the feasibility.

## Multipurpose applications of the e$^-$/e$^+$ beams and beamstrahlung photons

In addition to the specific physics cases highlighted above, the unique characteristics of the FCC-ee machine provide opportunities to leverage either beamstrahlung radiation or the unique high-energy injectors for a variety of physics applications. The following sections provide a more detailed exploration of these possibilities.

### Beamstrahlung radiation properties

At the interaction point (IP) of a particle collider, during each bunch crossing, the charged particles of a beam experience the electromagnetic (EM) field generated by the opposing bunch. They receive a strong beam-beam kick and their trajectories are bent, resulting in the emission of photons in a manner akin to synchrotron radiation. This process is called beamstrahlung. Like all the bremsstrahlung phenomena, beamstrahlung is particularly relevant for leptons and, therefore, for lepton colliders. In this case, beamstrahlung radiation can be described by the dimensionless Lorentz invariant parameter $\Upsilon$. This parameter changes during collisions, but for Gaussian beams its average value can be estimated by [181]

19 $$\begin{aligned} \Upsilon \approx \frac{5}{6}\frac{\gamma r_e^2 N_e}{\alpha \sigma _z(\sigma _x+\sigma _y)}~, \end{aligned}$$

where $\gamma$ is the relativistic Lorentz factor, $r_e$ is the classical electron radius, $N_e$ is the bunch population, $\alpha$ is the fine structure constant and $\sigma _{x,y,z}$ refers to bunch dimensions.

Since at the FCC-ee $\Upsilon \ll 1$ holds, the average number of photons $<n_\gamma>$ emitted per particle and their average energy $<E_\gamma>$ can be evaluated by

20 $$\begin{aligned} <n_\gamma >&\approx 2.54\frac{\alpha ^2\sigma _z}{r_e\gamma } \frac{ \Upsilon }{\sqrt{1+\Upsilon ^{\frac{2}{3}}}}~, \end{aligned}$$

21 $$\begin{aligned} <E_\gamma >&\approx E \times 0.462\Upsilon ~, \end{aligned}$$

where *E* is the electron or positron energy before radiating. Given the high bunch population and small transverse beam dimensions foreseen at FCC-ee, an extremely intense beamstrahlung radiation is expected.

As simulated with GuineaPig++ in another study [182], each colliding beam is expected to radiate $\sim 370$ kW and $\sim 77$ kW of beamstrahlung radiation at Z pole and $\textrm{t}\bar{\textrm{t}}$ operation, respectively, in case of no longitudinal shift and no transverse offset between the colliding bunches.

**Fig. 45:**
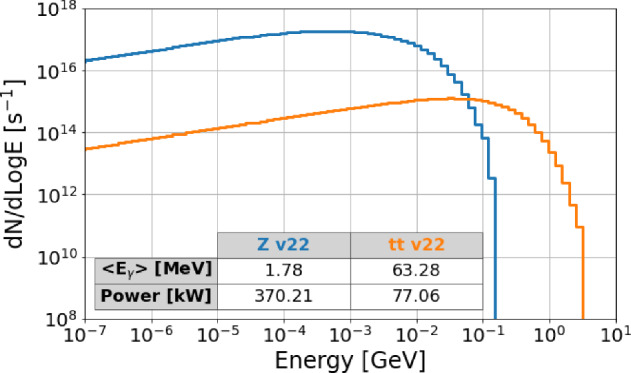
Beamstrahlung photon spectra for the two operation modes Z pole and $\textrm{t}\bar{\textrm{t}}$ of FCC-ee, as simulated with GuineaPig++ [182] for the FCC-ee optics version no. 22

Figure 45 reports the spectra of beamstrahlung photons for the aforementioned operation modes. They exhibit the typical shape of synchrotron radiation spectra, with single-photon energies extending up to $\sim 100$ MeV at the Z pole and up to a few GeV at the $\textrm{t}\bar{\textrm{t}}$ threshold.

**Fig. 46:**
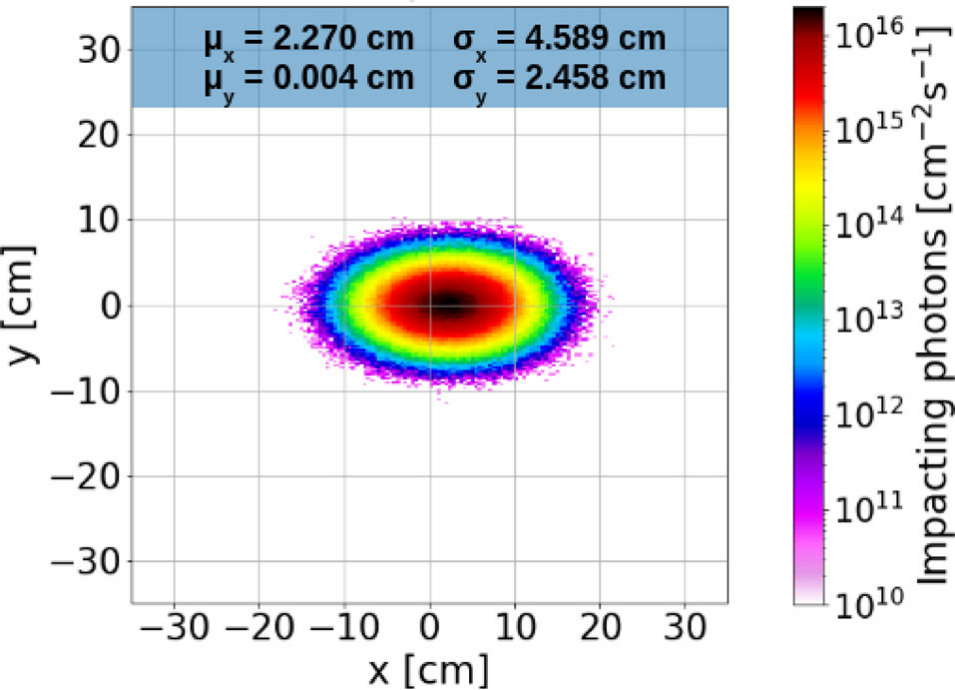
Beamstrahlung beam spot size at 500 m downstream of the IP for the Z pole operation mode

**Fig. 47:**
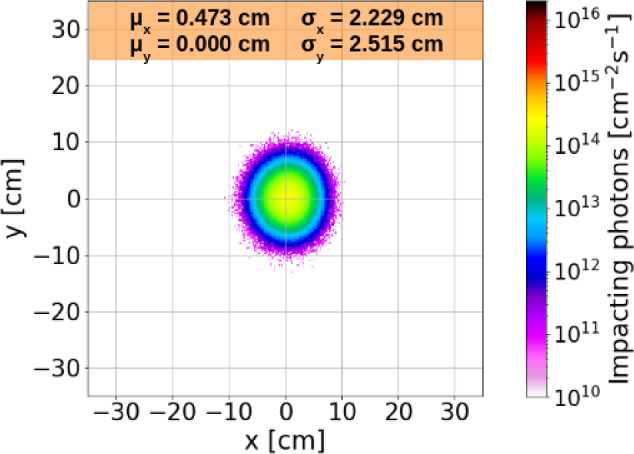
Beamstrahlung beam spot size at 500 m downstream of the IP for the $\textrm{t}\bar{\textrm{t}}$ operation mode

Beamstrahlung photons are emitted in a very narrow cone around the outgoing beams, with an opening angle inversely proportional to the beam energy. As a result, two intense photon beams exit each IP and—given the high intensities foreseen for FCC-ee—must be safely disposed in dedicated absorbers. In order to have enough separation from both the booster and the collider ring to integrate the shielded photon absorbers in the tunnel, beamstrahlung dumps will be located about 500 m downstream of the IP. This also avoids undesired background to the experiment detectors and implies a larger photon beam spot size. Figures 46 and 47 present the transverse spatial distributions of beamstrahlung photons at 500 m from the IP, i.e. where they would impact the dump. The difference in the spot sizes observed between the two operational modes is partly attributed to beam–beam effects in the horizontal crossing scheme at the interaction point. In particular, the different magnetic rigidities result in a broader horizontal photon distribution at the lowest beam energy (Z pole).

The design of such a dump is quite challenging, since its core must withstand high power densities and its shielding must contain most of the radiation showers developing from the interaction of the impinging photons, which produce EM cascades and induce photo-nuclear reactions. The Monte Carlo code FLUKA has been used to study the power deposition from beamstrahlung in a conceptual core, as well as the radiation levels in its proximity [183].

The radiation environment is dominated by EM showers with the additional contribution of MeV-scale neutrons from photonuclear reactions. Currently, the preferred dump design utilises circulating pure liquid lead, chosen for its ability to serve as a relatively compact absorber with a material highly effective at absorbing photons. However, a more conventional gas-cooled graphite absorber is also considered, as a back-up option.

**Fig. 48:**
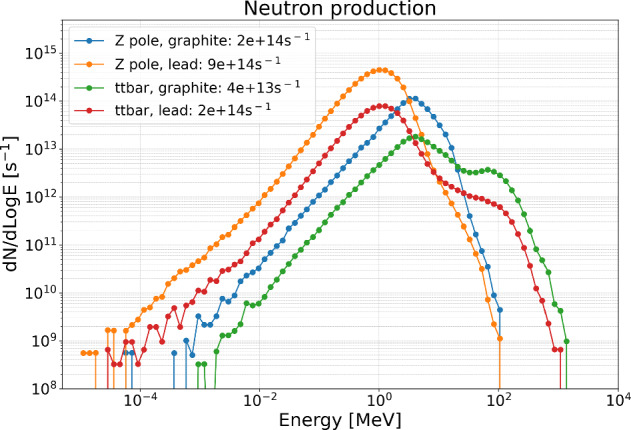
Spectra of neutrons produced inside the beamstrahlung dump, shown in lethargy representation, as simulated with the code FLUKA [184, 185] for two alternative dump materials and two different operation modes (see legend)

The impact of high energy and high intensity photon beams with the lead absorber generates a large amount of secondary particles, including neutrons, which could be used for other applications. The neutron production rate inside the beamstrahlung dump has been simulated with the code FLUKA [184, 185] for the two different conceptual designs, a 20-cm-long liquid lead pool and a 3-m-long graphite dump (Fig. 48). Even though the neutron production is significant, we caution that these spectra present the neutrons at production, which are not necessarily directly available for experimental purposes.

**Fig. 49:**
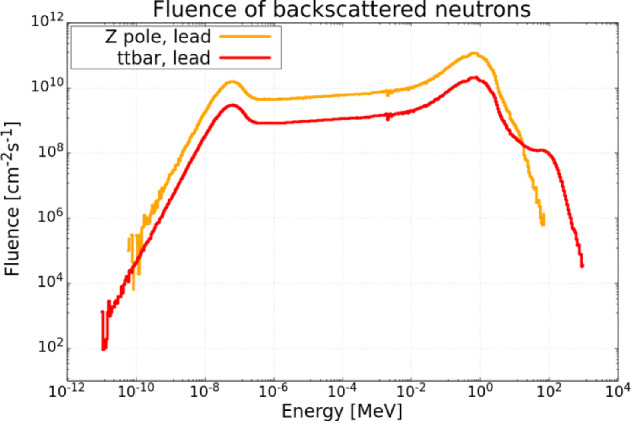
Volumetric density of neutron tracks per unit time, scored with FLUKA [184, 185] in a vacuum volume placed 20 cm in front of the liquid lead dump, and given in lethargy representation, for both Z pole and $\textrm{t}\bar{\textrm{t}}$ operation modes

To gain further insight, Fig. 49 displays the simulated fluence of neutrons in front of the dump, where two windows should be located to isolate the vacuum of the photon extraction line and the high temperature dump environment. This location can be a potential position for installing an experiment. Another possible location could be laterally displaced from the absorber itself.

With these photons being essentially available at no additional cost, there is a unique opportunity to harness their potential in innovative ways. Beamstrahlung photons consistently generate significant neutron fields across various scenarios, offering dual prospects: contributions to radionuclide production and the potential exploitation of neutron sources. To fully capitalise on this potential, key aspects to address include assessing neutron flux at realistic experimental positions, estimating background noise from electromagnetic showers, and managing mechanical integration constraints.

### Preliminary assessment of the radionuclide production possibilities at the FCC-ee

With its unique beamstrahlung radiation, the Future Circular electron-positron Collider (FCC-ee) offers a unique opportunity to explore radionuclide production. We have started to explore the potential for radionuclide production, using the photons and neutrons generated by the interaction of the photon beams with the dump absorbers, and explored possible applications and opportunities in nuclear physics and applied sciences, including the medical field.

**Fig. 50:**
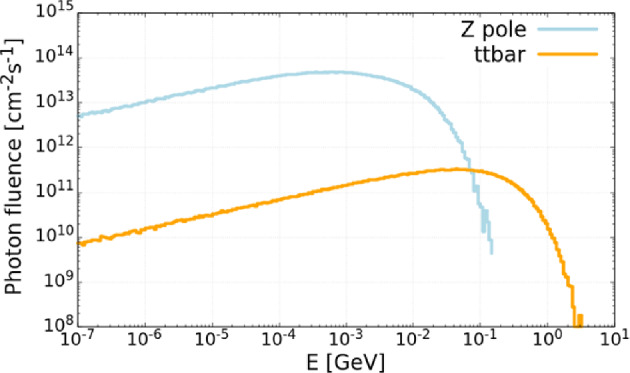
Volumetric density of photon tracks per unit time, scored with FLUKA in a vacuum volume placed 20 cm in front of the liquid lead dump, and given in lethargy representation, for both Z pole and $\textrm{t}\bar{\textrm{t}}$ operation modes. These photon fluences are the ones considered for the assessment of the radionuclide production possibilities

Figure 45 presents the energy spectra of beamstrahlung photons in the Z and $\textrm{t}\bar{\textrm{t}}$ modes of operation, as obtained from samples simulated with the code GUINEA-PIG++. Figure 50 displays the fluence of the same beamstrahlung photons as in Fig. 45 before any interaction with matter takes place, as evaluated with FLUKA in a vacuum volume located approximately 20 cm upstream of the dump (about 499.8 m downstream of the IP). The fluence is defined as the volumetric density of photon tracks inside a defined volume in units of cm/cm$^3$ = cm$^{-2}$ per unit time. All fluences are displayed in lethargy units, i.e. as $dN/d\log E = (dN/dE) \, E$, with $d \log E$ dimensionless.

**Fig. 51:**
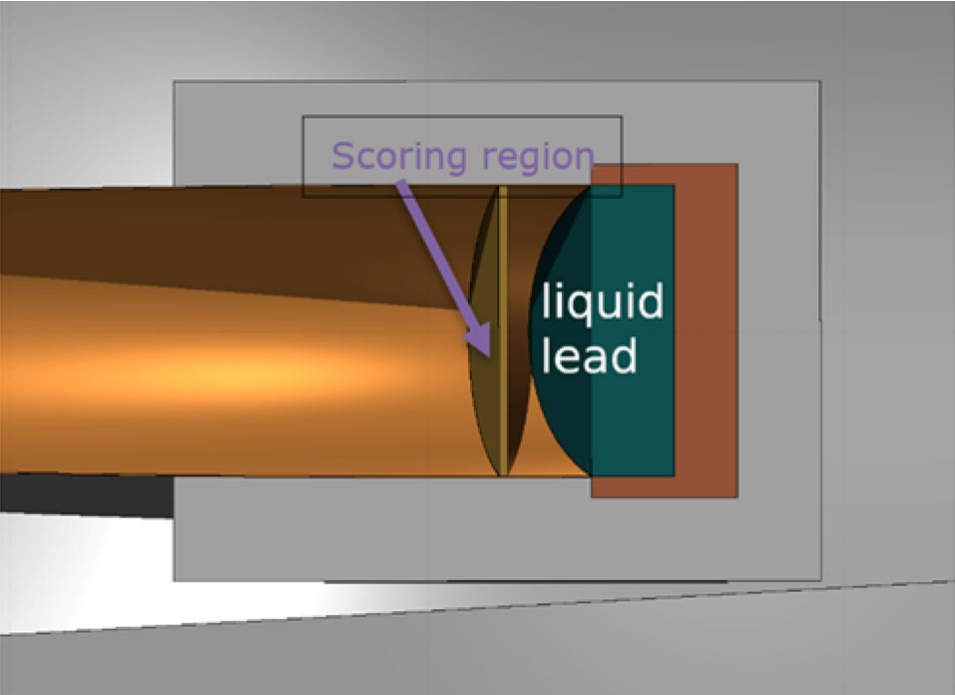
Scoring region in the vicinity of the beam dump used to assess the photon and neutron fluences for possible radionuclide production

In order to retrieve the photon (Fig. 50) and neutron fluences (Fig. 49) in the vicinity of the beam dumps, a scoring region was defined (Fig. 51), for the two operation modes on the Z pole and at the $\textrm{t}\bar{\textrm{t}}$ threshold. We assume that material specimens could be placed in the vicinity of the dump. Photonuclear reactions would be the main production channel, but the contribution from the neutrons back-scattered from the dump (Fig. 49) shall also be considered.

Each of the two operational modes may be optimally suited for producing different types of radionuclide, depending on the respective photon energy spectra and the associated production cross sections, and will yield complementary data.

For a preliminary assessment of the radionuclide production in this hitherto uncharted territory, the FLUKA code [184, 185] was used to produce the fluence spectra. Subsequently, we used the ActiWiz software [186], for calculating the radionuclide inventories based on those fluence spectra. A large set of data were obtained for 85 chemical elements. These production data can be compared with other parasitic production possibilities at CERN hadron facilities such as those available at ISOLDE and CHARM in the East area.

The aim of this preliminary study was to explore the potential of irradiation at FCC-ee for the nuclear physics community, but also for research and development in medical applications, by keeping in mind that this possibility would not substitute routine production. Photonuclear reactions are used and/or being explored in the medical field since decades (see references [187–189]) for the production of a variety of radionuclides such as Cu-67, Mo-99, Sn-117m etc.

In particular, for FCC-ee, the production of three specific radionuclides of medical interest has been studied: Ra-225/Ac-225, Mo-99/Tc-99m, Ti-44/Sc-44g.

Ra-225 (T1/2 = 15 days) acts as a generator of Ac-225, a radionuclide of high interest for Targeted Alpha Therapy (TAT) with a half-life of 9.9 days. The calculations show that production using Th instead of U will be more efficient both for production with hadrons and for production via photonuclear reactions. The $\textrm{t}\bar{\textrm{t}}$ running will be the preferred operation mode for Ra-225 production at FCC-ee, with a yield of $9\times 10^4$ nuclides/g/s. It should be highlighted that the yields achievable are FCC-ee are almost a factor 10 higher than those that can be reached in the parasitic irradiation station ISIS at ISOLDE (see Table 12).

**Table 12:** Ra-225 yield from Th and U in nuclides/g/s for different parasitic production modes

Mode	FCC ttbar	FCC Zpole	ISIS station 1 uA	CHARM grid	CHARM wall
Target	Nuclides/g/s	Nuclides/g/s	Nuclides/g/s	Nuclides/g/s	Nuclides/g/s
Th-232	$8.9\times 10^4$ ±0.6%	$7.7\times 10^3$ ±7.3%	$1.9\times 10^4$ ±1.1%	$1.5\times 10$ ±3.7%	$<<$ 1
U-238	$2.0\times 10^4$ ±2.8%	3.6 ±26%	$8.2\times 10^2$ ±4.3%	$<<$ 1	$<<$ 1

Ti-44 (T1/2 = 60 years) decays into Sc-44g (T1/2 = 4 h), the latter being of high interest for medical diagnosis but not sufficiently studied due to its difficult supply. Ti-44 acts as generator of Sc-44g that could be “milked” as secular equilibrium is reached after 24 h. The production of Ti-44 has been studied from different target elements (Ti, Cr, Fe, Mn, Ni, Sc, V) at the different facilities (FCC-ee, parasitic irradiation at ISOLDE/ISIS, and CHARM). The yields in nuclide/g/s show that in all cases production on Ti-nat should be favored. For Ti-44, the production via the Z pole operation mode at FCC-ee ($8\times 10^{6}$ nuclides/g/s) is 10 times higher than the one obtained at $\textrm{t}\bar{\textrm{t}}$ (hence, opposite to the Ra-225 case), due to its augmented cross section at the lower energies. The yields at FCC-ee are 10 to 100 times higher than at ISIS (see Table 13). Ti-44 generators with clinical amounts of activity (100 MBq or higher) still need to be explored [190]. By irradiating a 10 g target at the FCC-ee Z pole during one year, 10 MBq/g of Ti-44 could be generated. This would allow a constant feeding of 100 MBq of Sc-44g per 24 h for many years. Long time irradiation at FCC-ee would yield activities that are extremely difficult to achieve with a typical cyclotron or even at the CERN hadron facilities.

**Table 13:** Ti-44/Sc-44 production yield in Bq/g from natural titanium and for the different modes excluding the negligible amount from CHARM irradiations—all data are given after one day of cooling time

Mode	FCC ttbar	FCC Zpole	ISIS station 1 uA
Irradiation parameters	1 year irradiation [Bq/g]	1 year irr. [Bq/g]	2 weeks irr. [Bq/g]
Ti-44 activity	$1.0\times 10^4$ ±0.2%	$9.4\times 10^6$ ±0.1%	63 ±0.7%
Sc-44g activity	$1.9\times 10^6$ ±0.1%	$9.1\times 10^6$ ±0.1%	$3.5\times 10^5$ ±0.7%
Sc-44m activity	$1.7\times 10^6$ ±0.1%	$8.4\times 10^6$ ±0.1%	$3.2\times 10^5$ ±0.7%

Mo-99 (T1/2 = 66 h) is a well-known medical generator of Tc-99m, currently routinely used for cancer diagnosis purposes in the hospital. It is being produced world-wide in nuclear reactors. The typical Tc-99m injected dose is between 500 MBq and 1 GBq, to be compared with the weekly Mo-99 production in a reactor, that can reach a few TBq to 100 TBq to be dispatched to medical centres as generator of Mo-99/Tc-99m. However, the production at reactors is subject to shutdown and extended maintenance periods. Therefore, alternative options are being looked at and are of high interest for continued secure supply of this radionuclide to hospitals.

The production yield of Mo-99 on natural Mo via the Mo-100(g,n) reaction has been investigated. The predicted FCC-ee production yields are superior to those at hadron facilities (even compared with the ISOLDE in-target) with the Z pole operation mode being favorable ($6\times 10^{8}$  nuclides/g/s). After two weeks of irradiation and one day of cooling time, 435 MBq/g could be achieved at FCC Z pole. We remark that, due to the short half-life of Mo-99, one year of irradiation would yield the same activity after the end of the irradiation period (Table 14). It should be noted that this production is not only coming from photons (85 percent), since the neutron contribution is non-negligible (about 15 percent). Even if the attainable activity is interesting, it is still far from what is currently being achieved in reactors and what is needed for clinical applications.

**Table 14:** Mo-99 activity estimate in Bq/g from natural Mo and for the different modes excluding the negligible amount from CHARM irradiation—all data are given after one day of cooling time

Mode	FCC ttbar	FCC Zpole	FCC ttbar	FCC Zpole	ISIS station 1 uA
Irr. param.	2 weeks irr. [Bq/g]	2 weeks irr. [Bq/g]	1 year irr. [Bq/g]	1 year irr. [Bq/g]	2 weeks irr. [Bq/g]
Mo-99 act.	$1.97\times 10^7$	$4.35\times 10^8$	$2.02\times 10^7$	$4.50\times 10^8$	$3.07\times 10^5$

Summarising this section, in terms of science with radioisotopes, the production of radionuclides using beamstrahlung from FCC-ee presents some potential opportunities that warrant further study. For several medically relevant isotopes, although yield rates might be lower than with dedicated hadron-induced reactions,^3^ e.g. at ISOLDE, the possibility of a much longer irradiation time could produce activities for long-lived generator isotopes that exceed those attainable at medical cyclotrons or reactors. Potentially, there are other specific isotopes with half-lives longer than a few days that could be of interest to other scientific areas; for example, to generate sufficient quantities for experiments elsewhere, mirroring the studies performed at n_TOF using isotopes generated at ISOLDE to study nuclides important for the astrophysical s-process. To properly assess these opportunities, a wider study of isotope production using FCC-ee beamstrahlung would be required. In addition to irradiation opportunities, there might be examples of shorter-lived isotopes of interest to study online for nuclear physics or related sciences. While the level of resources needed for the implementation of a full-scale online system (as at the current ISOLDE Facility) might be difficult to envision in view of the relatively short duration of FCC-ee operation, special short-lived systems with extraction by fast transport tools such as pneumatic rabbits or similar methods are conceivable. The opportunities for direct use of beamstrahlung radiation for photonuclear reaction studies would also be worth consideration.

### A neutron source at FCC-ee

The use of neutron beam facilities concern several research fields including material science, solid-state physics, and nuclear physics.

For the study of neutron-induced nuclear reactions, accelerator-based neutron sources play an important role [191]. In particular, pulsed white neutrons sources are a versatile tool to study a wide range of energies for applications in nuclear technologies, in astrophysics, and in basic nuclear science [192]. For this class of neutron sources, which are different from the user-facility spallation neutron sources, the neutrons are produced by nuclear reactions of short pulses of protons or electrons incident on a heavy target.

To reduce the time spread inherent in the source-moderator ensemble, which deteriorates the achievable resolution in neutron energy, the charged-particle beams typically have pulse lengths on the order of several nanoseconds. To cover a large energy region, a hydrogen-rich moderator with a thickness in the order of centimeters is placed close to the production target in order to partially slow down the neutrons.

**Fig. 52:**
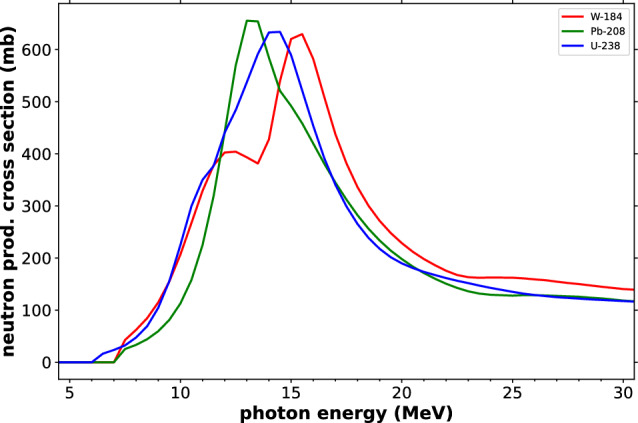
The total neutron production cross section versus photon energy for Pb, W and U, calculated with the nuclear reaction code TALYS [193]

Electron-beam-based sources produce neutrons in two steps. First, the electrons create bremsstrahlung in the target. This process is approximately proportional to $Z^2$, so favoring heavy mass target nuclei. The photons induce ($\gamma$,n), and if applicable ($\gamma$,f) reactions, i.e. reactions that either produce a neutron (n) or result in nuclear fission (f), respectively. Around energies of about 10 to 20 MeV the cross sections for photo-nuclear reactions increase considerably due to the giant electric dipole resonance, a collective excitation mode of the nucleus, as was illustrated in Fig. 19. This cross section is roughly proportional to *NZ*/*A*; so, it again is more advantageous for heavy-mass nuclei. The cross section for gamma-induced neutron production, calculated with the nuclear reaction code TALYS [193], is shown in Fig. 52 for uranium, tungsten and lead.

**Fig. 53:**
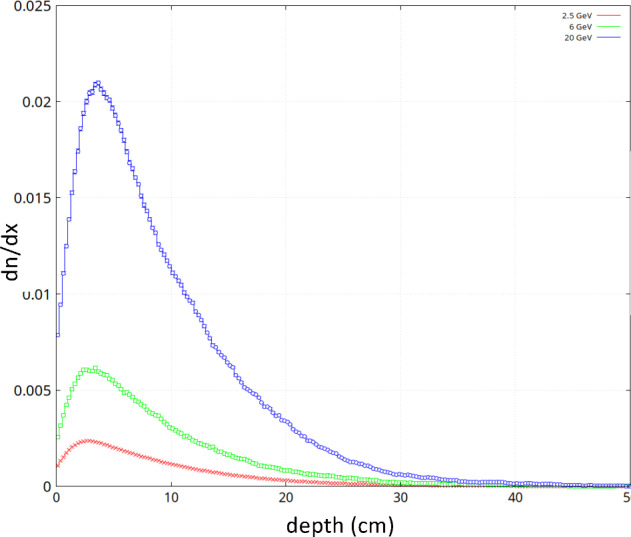
The neutron production as a function of depth in a tungsten target of 50 cm length and 20 cm radius for electron energies of 2.5, 6 and 20 GeV (data courtesy of V. Vlachoudis) [194]

Simulations, performed with the code FLUKA [184, 185], for a simple geometry, consisting of a cylinder of 20 cm radius and 50 cm length, considered three materials (uranium, tungsten, and lead), which were irradiated by a pencil electron beam with different electron energies (2.5, 6 and 20 GeV). The results reveal several important features [194]. The aforementioned geometry was chosen since the neutron production stays mostly within the target, as can be seen in Fig. 53, where the neutron production is shown as a function of depth in a tungsten target. Similar figures were obtained for uranium and lead.

**Fig. 54:**
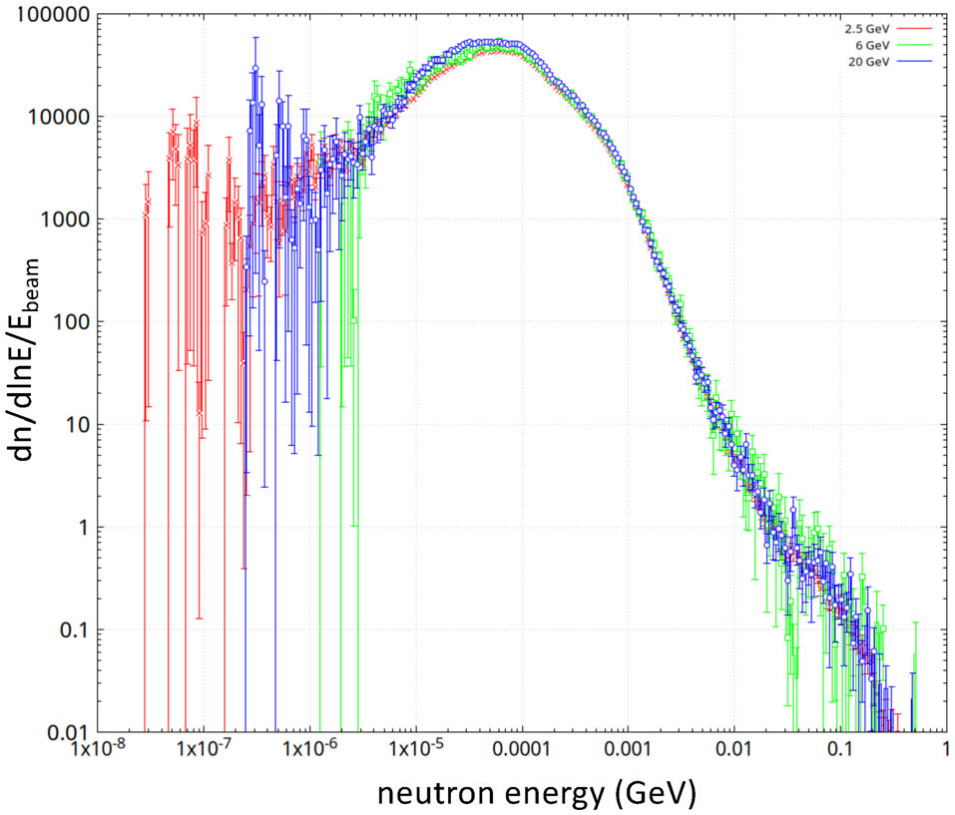
Neutron energy distribution normalised to the electron beam energy, in the tungsten target for three electron-beam energies (data courtesy of V. Vlachoudis) [194]

**Fig. 55:**
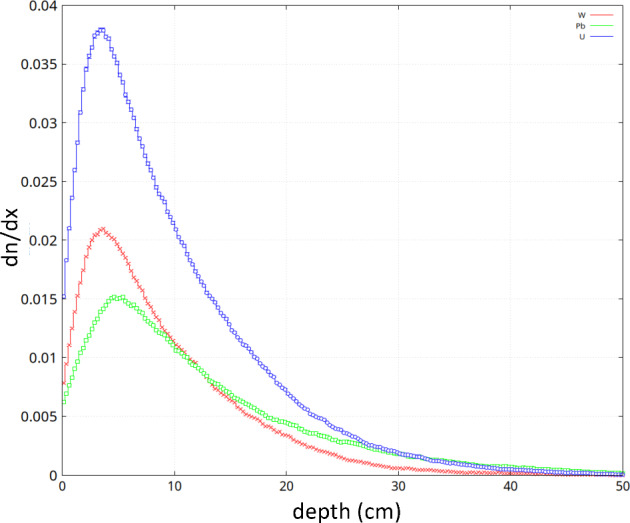
Neutron production as a function of depth for electrons of 20 GeV in the targets uranium, tungsten and lead (data courtesy of V. Vlachoudis) [194]

From the simulations at different electron energies, it is clear that the neutron production yield scales roughly linearly with the electron energy, as is illustrated in Fig. 54, displaying the neutron energy distribution normalised to the electron beam energy. The neutron production for 20 GeV electrons in the target volume for the three materials is shown in Fig. 55. From this figure we can extract the neutron production for the above geometry, which is approximately 0.05 per electron per GeV for uranium, and 0.025 neutrons per electron per GeV for both tungsten and lead.

As highlighted in the recent NuPECC Long Range Plan [195], four neutron time-of-flight facilities are currently operating in Europe, of which only two have a moderator allowing to cover a large range in energy down to thermal energies. Because of the presence of slow neutrons from the moderator with long times of flight, the repetition rate of the accelerator’s charge-particle pulses needs to be low enough to avoid overlapping neutron pulses. The facility GELINA at EC-JRC-Geel [196] uses an electron beam and a uranium target, while the n_TOF facility at CERN [197], operational since 25 years, makes use of the Proton Synchrotron (PS) beam. The very high instantaneous intensity of the n_TOF neutron source per proton pulse, together with its two flight paths of 20 and 185 m, and an irradiation station close to the target, makes it an excellent facility for top-level research on neutron-nucleus interactions.

The concept of the FCC-ee potentially provides an interesting opportunity for the construction of a complementary neutron source. A pulsed electron beam can be used for enabling neutron time-of-flight experiments, in the field of nuclear physics. Coupled to a moderator, the neutron energy range will cover a wide spectrum extending down to thermal neutrons. Such a facility would extend the physics possibilities into areas beyond what is presently offered by the existing n_TOF facility at CERN. To further optimise its physics potential, the instantaneous pulse intensity of the electron linac should be maximised.

## Conclusions and outlook

The Future Circular Collider (FCC)-integrated programme represents a transformative initiative in particle physics and accelerator technology. Expected to operate from the mid-to-late 2040s through the early 2060s, the FCC-ee will deliver unprecedented luminosities to four experiments, while maintaining a focus on sustainability and energy efficiency. Its key features include top-up injection from a full-energy booster, the world’s most intense positron source, and high-energy injector linacs. These elements not only ensure optimal performance for collider operations, but, at the same time, the **unprecedented parameters of the FCC-ee accelerator complex also open up tantalising new frontiers in fundamental and applied physics**.

Beyond its *core collider function*, the FCC-ee offers **unique opportunities across various disciplines**. Its high beam energies (20 – 183 GeV), low-emittance beams, and intense positron production enable applications ranging from true muonium production and Bose-Einstein condensates of positronium to high-brightness X-ray science and Compton imaging. Additionally, the facility’s high-power beamstrahlung photons (up to 0.5 MW per beam per collision point) present novel possibilities for neutron generation, radioactive isotope production and other advanced research applications.

As both a next-generation collider and a **versatile scientific platform**, the FCC-ee stands **at the forefront of innovation**. Its capabilities do not only promise groundbreaking discoveries in particle physics, but also significant contributions to adjacent fields, reinforcing its role as a **cornerstone of future scientific exploration**.

## Data Availability

Most data and pictures presented were taken from the cited references. Data sets generated during the current study, e.g. SPECTRA simulation results, are available from the authors on reasonable request. The manuscript has associated data in a data repository.

## Footnotes

1 Here, “electrons” means either electrons or positrons.

2 The Compton recoil factor *X* is defined as $X=4 E_eE_{ph}/({m_e}^2c^4)$ with $E_e$ and $E_{ph}$ denoting the initial electron and photon energies.

3 Typical cross sections are 10–1000 times larger for hadronic reactions than for photonuclear ones, depending on energy.
